# Factors affecting the uptake of preventive chemotherapy treatment for schistosomiasis in Sub-Saharan Africa: A systematic review

**DOI:** 10.1371/journal.pntd.0009017

**Published:** 2021-01-19

**Authors:** Carlos A. Torres-Vitolas, Neerav Dhanani, Fiona M. Fleming

**Affiliations:** 1 SCI Foundation, London, United Kingdom; 2 School of Public Health, Imperial College, London, United Kingdom; University of Texas Health Science Center, UNITED STATES

## Abstract

**Background:**

Schistosomiasis affects nearly 220 million people worldwide, mainly in Sub-Saharan Africa (SSA). Preventive chemotherapy (PC) treatment, through regular mass-drug administration (MDA) of Praziquantel tablets remains the control measure of choice by Ministries of Health. Current guidelines recommend that 75% of school-aged children receive treatment. Many programmes, however, struggle to achieve this target. Given the risk of high reinfection rates, attaining sustained high levels of treatment coverage is essential. This study provides a comprehensive review of the barriers and facilitators operating at different levels of analysis, from the individual to the policy level, conditioning the uptake of PC for schistosomiasis in SSA.

**Methodology/Principal findings:**

A systematic literature search was conducted in several databases for publications released between January 2002 and 2019 that examined factors conditioning the uptake of Praziquantel in the context of MDA campaigns in SSA. A total of 2,258 unique abstracts were identified, of which 65 were selected for full text review and 30 met all eligibility criteria. Joanna Briggs Institute’s Critical Appraisal and the Mixed-Methods Assessment tools were used to assess the strength of the evidence. This review was registered with PROSPERO (CRD42017058525).

A meta-synthesis approach was used. Results indicated publication bias, with the literature focusing on East African rural settings and evidence at the individual and programmatic levels. The main influencing factors identified included material wellbeing, drug properties, knowledge and attitudes towards schistosomiasis and MDAs, fears of side effects, gender values, community and health systems support, alongside programme design features, like training, sensitisation, and provision of incentives for drug-distributors. The effect of these factors on determining Praziquantel uptake were explored in detail.

**Conclusions/Significance:**

Multiple determinants of treatment uptake were found in each level of analysis examined. Some of them interact with each other, thus affecting outcomes directly and indirectly. The promotion of context-based transdisciplinary research on the complex dynamics of treatment uptake is not only desirable, but essential, to design effective strategies to attain high levels of treatment coverage.

## Author summary

Schistosomiasis is a parasitic infection that affects nearly 220 million people worldwide. Long-term effects include anaemia, growth stunting, bladder cancer and infertility. Currently, the main approach to schistosomiasis control involves mass preventive chemotherapy treatment. Current guidelines recommend treating 75% of school-aged children but many programmes struggle to achieve this target. This study conducted a comprehensive review of factors conditioning the uptake of treatment in Sub-Saharan Africa across different levels of analysis: individual, interpersonal, organisational, community and policy. This examination showed, first, that the literature suffers of publication bias, with most studies based in rural East African sites and focusing in discussing individual- and policy-level factors. It indicated as well that people’s livelihoods, food security, and school-enrolment, alongside knowledge and attitudinal factors influenced treatment uptake at the individual level. Various contextual factors concerning interpersonal relations, organisations’ resources, and prevalent socio-cultural features (e.g., gender) further shaped people’s responses to MDA campaigns. Finally, it was observed that the effectiveness of programme-level decisions on sensitisation, training, and drug-delivery strategies were constantly re-shaped by mediating factors operating at lower levels of analysis. We conclude that the promotion of context-based transdisciplinary research is essential to design effective strategies to promote sustainable high levels of treatment coverage.

## Introduction

Schistosomiasis is a neglected tropical disease (NTD) that affects nearly 220 million people worldwide [1]. It consists of a parasitic infection caused by *Schistosoma* trematodes, transmitted through contact with water contaminated by infected faeces or urine, where snail intermediate hosts are present. Depending on the species, the urogenital or gastrointestinal tracts can be affected. The disease mostly leads to disability rather than death [2]. Long-term infection is associated with anaemia, growth stunting, impairment of cognitive development and work capacity and, in later life, bladder cancer and infertility [2,3]. Schistosomiasis is considered a ‘disease of poverty’, being endemic in tropical and subtropical regions lacking adequate water or sanitation infrastructure. Over 90% of all *Schistosoma* infections are located in sub-Saharan Africa (SSA) [1].

Drawing on its first roadmap to overcome schistosomiasis globally, agreed in 2012 [4], the World Health Organisation (WHO) is currently revising its goals for the 2021–30 period. Its proposals include eliminating schistosomiasis as a public health problem by 2030, defined as achieving a ≤1% prevalence of heavy-intensity infections [5]. The following strategies have been proposed to control schistosomiasis’ endemicity: (i) improving water and sanitation infrastructure, (ii) enhancing health and hygiene education, (iii) controlling snail populations with molluscicides, and (iv) implementing preventive chemotherapy (PC) interventions through regular mass-drug administration (MDA) of Praziquantel (PZQ) tablets [6]. To date, MDA campaigns have been prioritised since they can rapidly reduce disease prevalence in a cost-effective manner [7–9].

Current WHO’s guidelines recommend that MDAs should treat 75% of school-aged children (SAC) [10]. Modelling studies, however, suggest that whilst these targets may be adequate in moderate and low transmission settings, higher levels of therapeutic coverage are required in high-transmission locations (>50% prevalence) [11,12]. Moreover, there is agreement that prolonged high coverage rates are necessary to control the disease, given that few untreated individuals can yield a large number of eggs in the environment, potentially generating a rebound in disease prevalence despite MDA activities [13,14]. Crucially, official reports indicate that, despite recent progress, Africa is yet to achieve recommended targets. As of 2019, around 61.8 million SAC and 11.2 million adults received PC against schistosomiasis in the region, representing 57.1% and 11.9% of those deemed to require treatment for each age group [15].

Identifying the barriers and facilitators that affect programmes’ capacity to achieve recommended targets constitutes a challenge given MDAs’ complexity. PC interventions involve various components (e.g., sensitisation, drug-procurement, distribution and reporting) as well as organisations (e.g., Ministries of Health, Education, and organised communities), whose interactions cut across different levels of influence (national to local), so that their impacts on final outcomes are non-linear. A wide range of factors might, therefore, be influential to outcomes. At the programmatic level MDAs can adopt different operational strategies concerning drug-delivery platforms (school- or community-based), target populations (SAC or SAC alongside adults), and frequency (annually or biennially), depending on endemicity and countries’ policies [10]. These decisions can generate multiple implementation challenges, including intersectoral coordination, training, supervision, and staff incentives issues [16–18]. The effectiveness of such decisions, in addition, are further conditioned on the acceptance and support of target groups and their communities. Socio-cultural considerations such as rumours of deaths, fears of side effects, or traditional beliefs have been found to condition acceptance of PC [19–21].

Examinations of the aforementioned dynamics remains underdeveloped in the NTDs literature. Past reviews have mainly explored programmatic issues rather than socio-cultural factors. Recent reviews by Corley [18], Krentel [17], and MacFarlane and colleagues [22], for instance, focused on staff considerations. They discussed, respectively, the roles that nurses and community health workers play during MDAs, the factors motivating drug-distributors to support MDA activities, and the state of the policy guidelines and institutional support shaping drug-distributors’ work. All of them, with different emphases, highlighted the value of integrating front-line workers into public health systems. Another review by Burnim’s et al. [21] concerned with the effectiveness of school-based against community-based drug-distribution platforms, concluding that neither approach alone is likely to reach the 75% target consistently.

Concerning socio-cultural issues, in turn, substantive knowledge gaps remain. A 2018 study led by Sacolo examined publications describing knowledge, attitudes and practices about schistosomiasis in SSA [23], reporting that residents customarily lack adequate knowledge about the mechanisms of transmission and prevention of the disease and that misconceptions about schistosomiasis and MDAs are widespread. Since this review was not framed in the context of MDAs, however, it is not possible to ascertain a direct connection between those factors and treatment coverage. Burnim and colleagues [21], in turn, identified some barriers to treatment uptake, such as fear of side effects, socioeconomic conditions, and lack of incentives for drug-distributors. Nevertheless, since this work was mainly concerned with assessing the effectiveness of drug-delivery platforms, it did not explore such issues in-depth. Moreover, neither of these reviews analytically distinguished between different types of knowledge and attitudes (e.g., perceptions about the disease’s seriousness, treatment’s health benefits, or distributor’s competence) that could elucidate their specific relevance, nor were they concerned with establishing how individuals’ characteristics could be linked to higher-level conditioning factors (e.g., community or policy issues).

The objective of this review is to identify the factors that condition the uptake of PC treatment for schistosomiasis in SSA. To this effect, this study produced a narrative synthesis of qualitative and quantitative evidence from SSA concerning barriers and facilitators operating at different levels of analysis, from the individual to the policy level. The analytical framework that guided this synthesis was the socioecological model of health behaviour [24,25]. This approach postulates that interventions’ outcomes are the result of the interaction between factors of different nature (social, physical or cultural) that operate at multiple levels of influence. It customarily distinguishes between the following ones: (i) intrapersonal (individuals’ attitudes, beliefs and socio-demographic characteristics); (ii) interpersonal (relationship- and group-based interactions); (iii) organisational (formal and informal rules and ethos used by organisations responsible for the intervention); (iv) community (forms association, governance, shared values, and environmental factors); and (v) policy (laws and policies, regional or national, that formally direct an intervention). Evidence-based lessons will be drawn to inform future initiatives aimed at achieving high and sustainable levels of therapeutic coverage. Particular attention will be paid to the WHO’s ‘leaving no one behind’ agenda, which demands that NTD control programmes reach marginalised populations, such as women and the extreme poor [26].

## Methods

### Eligibility

This review included any peer-reviewed journal articles published between January 2002 and January 2019 that empirically examined factors conditioning the uptake of Praziquantel for schistosomiasis in SSA, in the context of MDA campaigns. Year 2002 was chosen as the starting date to coincide with the first guidelines for helminth control in school-age children released by the WHO and the advent of national-scale control programmes for schistosomiasis [27]. The study’s **regional scope was limited to SSA since both schistosomiasis infections and PC control strategies are concentrated in the region** [1]. Opinion and review pieces were excluded.

Qualitative, quantitative, and mixed-method studies were considered acceptable given the study’s aim to identify barriers and facilitators in a comprehensive manner. No restrictions were applied regarding drug-distribution platforms or target populations. Selected examinations of MDAs could include school-based treatment (SBT), community-wide treatment (CWT), or alternative forms of distribution aimed at either SAC (5 to 14 years of age) or SAC and adults (15+ years of age).

Two sets of outcome measures were considered acceptable. For quantitative analyses these included observed or self-reported treatment coverage (percentage of the eligible populations that received treatment) or treatment compliance estimates (percentage of eligible populations who were offered praziquantel and swallowed them) [28]. For qualitative assessments, acceptable measures included observed or self-reported expressions of acceptance, trust or resistance towards MDAs. To be included in the final review, publications had to provide descriptions, explanatory models, or interpretations of conditioning factors’ effects on outcome measures.

A version of this study’s protocol was registered at the International Prospective Register of Systematic Reviews (PROSPERO) (No: CRD42017058525).

### Search strategy

A comprehensive literature search was conducted in July 2017 and replicated in January 2019 to update the list of articles for screening. The search strategy was developed by two team members (CTV and FMF), based the Problem/Population, Intervention, Comparison, and Outcome (PICO) framework [29], albeit discarding the ‘comparison’ criterion due to the limited use of control groups in the literature (Table 1). An initial version of the search string was trialled during April 2017 to ensure its appropriateness.

**Table 1:** Search terms.

Dimension	Terms	Connector
Problem	schistosom* OR bilharzia OR “snail fever” OR helminth*	AND
Population	Angola OR Benin OR Botswana OR "Burkina Faso" OR Burundi OR Cameroon OR "Cape Verde" OR "Central African Republic" OR Chad OR Congo OR "d'Ivoire" OR "Ivory Coast" OR Djibouti OR "Equatorial Guinea" OR Eritrea OR Ethiopia OR Gabon OR Gambia OR Ghana OR Guinea OR "Guinea Bissau" OR Kenya OR Lesotho OR Liberia OR Madagascar OR Malawi OR Mali OR Mauritania OR Mauritius OR Mozambique OR Namibia OR Niger OR Nigeria OR Rwanda OR "Sao Tome" OR "São Tomé" OR Senegal OR "Sierra Leone" OR Somalia OR "South Africa" OR Sudan OR Swaziland OR Tanzania OR Togo OR Uganda OR Zambia OR Zimbabwe	AND
Intervention	Praziquantel OR treatment* OR intervention* OR antihelminth* OR “preventive chemotherapy” OR “control program” OR “control programme” OR “drug-distribution” OR “drug-administration” OR “drug-delivery” OR MDA$	AND
Outcome	access* OR coverage OR uptake OR compliance OR adherence OR participati* OR accept* OR satisfaction OR response* OR resist* OR rejecti* OR avoid* OR trust OR mistrust	-

**Fig 1:**
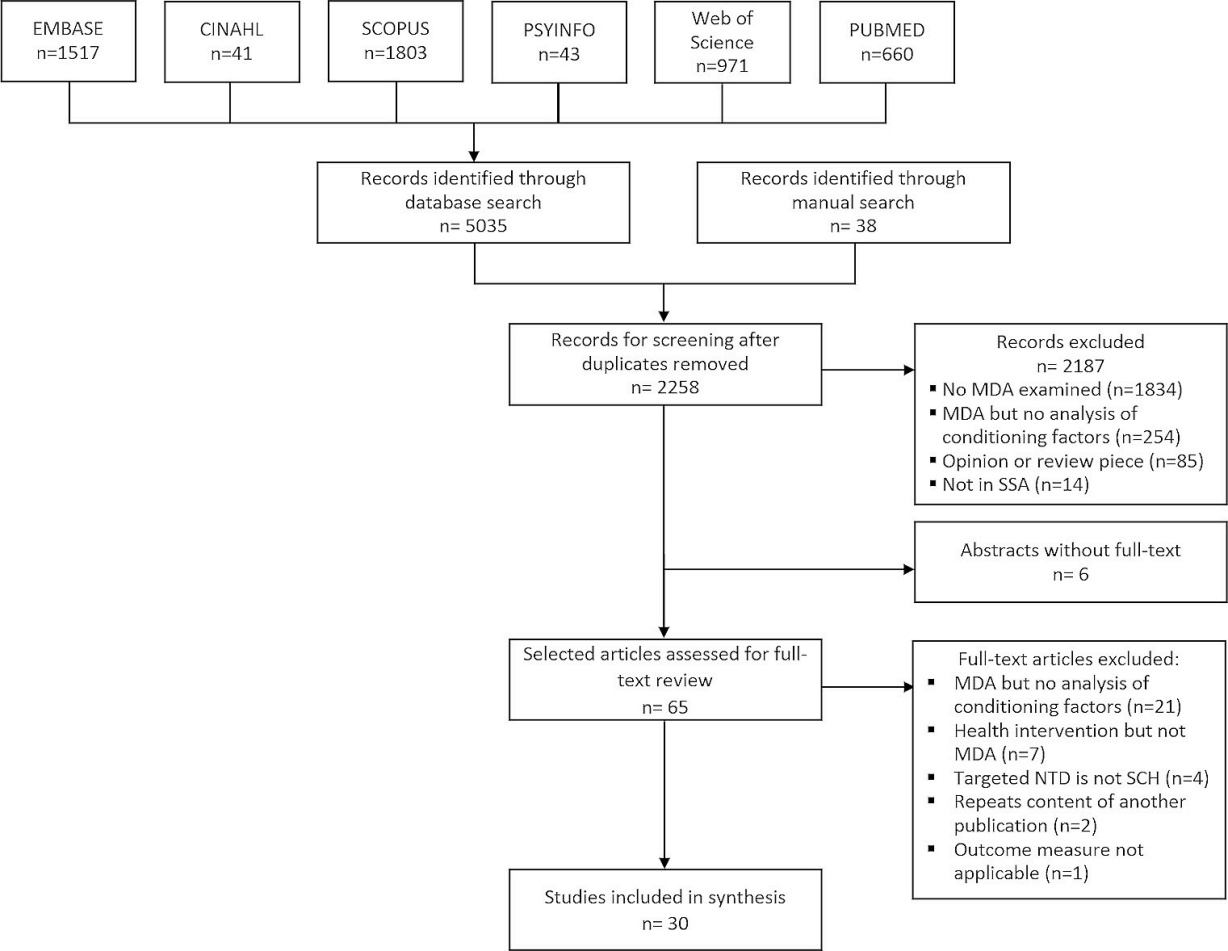
PRISMA flow diagram.

Bibliographic databases EMBASE, CINAHL, SCOPUS, PsyInfo, Web of Science, and PubMed / MEDLINE were searched. To ensure completeness, three external academics (See acknowledgements) were consulted to recommend any studies considered valuable to this review. Additionally, a manual search of publications was conducted by examining the full list of references used in recent systematic reviews relevant to the fields of MDAs and schistosomiasis [17,21–23,28,30,31]. A total of 2,258 unique abstracts were obtained for abstract screening, of which 65 were selected for full text review. The lead author (CTV) worked in pairs with a co-author (ND) and two assistants (See acknowledgments) to complete the full text review and decide on the publications’ final inclusion. Thirty articles met all eligibility criteria (Fig 1). EPPI-Reviewer v.4 was used throughout this process [32].

### Quality assessment

Adapted versions of the Joanna Briggs Institute’s Critical Appraisal tools were used to assess studies’ quality according to their design: qualitative or quantitative (cross-sectional, cohort, or randomized controlled trials) [33]. For mixed-methods studies, the Mixed-Methods Assessment tool [34] was integrated into the Joanna Briggs’ instruments to examine their overall design (See S1 Table). The lead author (CTV) paired with a co-author (ND) and two study assistants (See acknowledgements) to agree on the final score for each selected publication. No articles were discarded based on final scores since descriptive data in studies of ‘low’ methodological quality may still render valuable insights for a narrative review [29]. The variety of study designs, in addition, may have rendered this type of exclusion criterion exceedingly complex and open to challenges. Quality assessments served instead to indicate the strength of the evidence.

### Data extraction and synthesis

This study adopted a meta-synthesis approach [35] to systematically aggregate evidence into a narrative synthesis. Data extraction was conducted in two stages. First, descriptive information on MDAs and methods were pulled from each paper to provide contextual information. Forms included information about country and setting, target populations, distribution strategy, drugs, study design, data-collection tools and sample size, as well as coverage results (Table 2). Second, an open coding exercise was conducted to label summary findings from qualitative studies. Codes were reviewed and grouped by the lead author after comparing and juxtaposing them to determine similarities and differences in meaning. Resulting codes were grouped into sub-themes and framed within the overall five levels of analysis of socio-ecological model of health behaviour. Results from the qualitative synthesis were merged with quantitative results following a thematic approach. Review meetings were held with the entire team to confirm final structure. The list of categories and sub-themes are reported in Table 3. (Full data-extraction: S2 Table).

**Table 2:** Summary of MDA interventions and evaluation methods in selected publications.

Study	Country	Target groups	Drugs	Type of MDA	Study design	Setting	Length of study	Methods and Sample size	Coverage (drug, source)	Quality Appraisal^(a)^
Adeneye et al., 2007 [36]	Nigeria	SAC	PZQ	SBT	Qualitative	Rural (2 villages)	9 months	-Focus group discussions (FGD) with parents (n-FGDs = 16)^(b)^ -FGD with children (age: 5–12) (n-FGDs = 16)^(b)^ -FGD with adolescents (age: 13–19) (n-FGDs = 16)^(b)^ -Interviews with community leaders (n = 8)	-SAC: 28.5% (PZQ, registers)	6/10
		SAC	PZQ	CWT		Rural (2 villages)			-SAC: 72.2% (PZQ, registers)	
		SAC	PZQ	Primary health care (PHC)		Rural (2 villages)			-SAC: 44.3% (PZQ, registers)	
Adriko et al., 2018 [37]	Uganda	SAC Adults	PZQ	SBT CWT	Quantitative: Cross-sectional	Rural (2 villages)	1 month	-Household census (n-households = 681, n-individuals = 3,208).	-SAC: 70.7% (95% CI: 67.6% - 73.6%) (PZQ, surveys) -SAC and adults: 46.5% (95%CI: 44.5% - 48.5%) (PZQ, surveys)	7/9
Bogus et al., 2016 [38]	Liberia	SAC Adults^(c)^	PZQ, Albendazole (ALB), Ivermectin (IVM)	CWT	Quantitative: Cross-sectional	Rural (32 villages)	1 month	-Opinion survey with village leaders (n = 140).	-N.A. (MDA interrupted due to Ebola epidemic).	2/9
Bukindu, Morona and Mazigo, 2016 [39]	Tanzania	SAC	PZQ, ALB	SBT	Quantitative: Cross-sectional	Rural (5 schools)	1 month	-Survey with primary schoolchildren, grades 3 to 6 (age: 8–18) (n = 625).	-SAC: 95.6%, 95%CI (92.8%-98.5%) (PZQ and ALB, survey)	6/9
Chami et al., 2016 [40]	Uganda	SAC Adults	PZQ, ALB, IVM	CWT	Quantitative Cross-sectional	Rural (17 villages)	4 months	-Household surveys (n-households = 510; n-individuals = 935)	-SAC and adults: 52.6% (PZQ, drug receipts)^(d)^	8/9
Chami et al., 2017 [41]	Uganda	SAC Adults	PZQ, ALB, IVM	CWT	Quantitative: Cross-sectional^(e)^	Rural (17 villages)	1 month	-Household census (n-households = 3491, n-individuals = 16,357). -FGD with community drug-distributors (CDDs) (n-FGDs = 6, n-distributors = 34)	-SAC and adults: 38.2% (PZQ, census)^(d)^	9/9
Coulibaly et al., 2018 [42]	Cote d’Ivoire	SAC Adult	PZQ	CWT	Quantitative Cross-sectional	Rural (2 villages)	1 month	-Surveys with SAC and adults (n = 405)	-SAC and adults: 47.6% (PZQ, registers) -SAC and adults: 34.6% (PZQ, surveys)	4/9
Dabo et al., 2013 [43]	Mali	SAC Adults	PZQ, ALB	CWT	Mixed-methods	Rural (10 villages)	1 year	-Statistical analysis of registers (n-SAC = 3026, n-adults = 4996). -FGD with adults (n-FGDs = 10, n-adults = 100) -Interviews with village leaders^(b)^ -Interviews with CDDs^(b)^ -Interviews with adults (n = 100).	-SAC and adults: 76.7% (PZQ and ALB, registers) -SAC: 78.1% (PZQ and ALB, registers) -Adults: 75.4% (PZQ and ALB, registers)	8/15
Fleming et al., 2009 [16]	Uganda	SAC Adults	PZQ, ALB	SBT CWT	Qualitative	Rural and Urban (20 districts)	3 years	a. 2003–2005 (18 districts) -Interviews with district officials (n-2003 = 74, n-2004 = 53, n-2005 = 28). -Interviews with local leaders(n-2003 = 23, n-2004 = 136, n-2005 = 19) -Interviews with health workers (n-2003 = 13; n^-2004^ = 0, n-2005 = 42) -Interviews with drug-distributors and teachers (n-2003 = 52, n^-2004^ = 184, n^-2005^ = 293) -Interviews with beneficiaries at schools (n^-2003^ unreported, n^-2004^ = 1060, n^-2005^ = 173). -Interviews with beneficiaries in communities (n^-2003^ unreported, n^-2004^ = 883, n^-2005^ = 79). b. 2006 study (2 districts): -FGD with district officials (n-FGDs = 2) ^(b)^ -FGD with local leaders (n-FGDs = 2)^(b)^ -FGD with health workers (n-FGDs = 2)^(b)^ -FGD with CDDs and teachers (n-FGDs = 11, n-teachers = 89) -FGD with beneficiaries at schools (n-FGDs = 7, n-beneficiaries = 64) -FGD with individual beneficiaries at communities (n-FGDs = 30, n-beneficiaries = 320)	-76.6% (Nebbi, PZQ and ALB, registers)^(f)^ -80.4% (Busia, PZQ and ALB, registers)^(f)^	7/10
Hastings, 2016 [44]	Tanzania	SAC	PZQ, ALB	SBT	Qualitative	Urban (1 squatter area) Rural (1 village)	3 years	-Interviews with villagers, health practitioners, drug-distributors, healers and village leaders (n = >200) -Participatory observation. -Documents’ review (health registers).	-N.A. (MDA interrupted due to riots).	7/10
Knopp et al., 2016 [45]	Tanzania	SAC Adults	PZQ, ALB	SBT CWT	Quantitative Cross-sectional^(e)^	Urban and Rural (Pemba: 45 shehias and 45 schools; Unguja: 47 shehias and 48 schools)	3 months	-Surveys with adults in shehias (age: 20–55) (n- Unguja = 2323; n- Pemba = 2231); -Surveys with SAC in schools (age: 9–12) (n- Unguja = 3295; n- Pemba = 5036) -Interviews with community members^(b)^	-SAC: 85.2% (95%CI: 81.8%–88.6%) (Pemba), 86.9% (95%CI: 81.0%–92.9%) (Unguja) (PZQ, registers). -Adults: 60.1% (95%CI: 56.5%–63.7%) (Pemba), 71.2% (95%CI: 66.6%–75.8%) (Unguja) (PZQ, registers).	6/9
Lothe et al., 2018 [46]	South Africa	SAC	PZQ	SBT	Qualitative	Rural (6 schools)	4 months	-FGD with pupils (n-FGDs = 12, n = 75) -Interviews with pupils (n = 12) -Interviews with teachers (n = 6) -Interviews with healers (n = 3) -Interviews with health worker (n = 1)	-SAC: 50–75% in 3 schools (PZQ, registers) -SAC: 10–20% in 3 schools (PZQ, registers)	8/10
Mafe et al., 2005 [47]	Nigeria	SAC	PZQ	PHC	Quantitative Cross-sectional	Rural (2 villages)	2 months	-Surveys with SAC (n = 242)	-SAC: 60.0% SAC (PZQ, survey)^(d)^	5/9
		SAC	PZQ	SBT		Rural (2 villages)			-SAC: 49.2% SAC (PZQ, survey) ^(d)^	
		SAC	PZQ	CWT		Rural (2 villages)			-SAC: 77.2% SAC (PZQ, surveys)^(d)^	
Massa et al., 2009a [48]	Tanzania	SAC	PZQ, ALB	SBT	Qualitative	Rural (5 villages)	1 year	-Two rounds of interviews with village leaders (n = 10) -Two rounds of interviews with teachers (n = 5) -Two rounds of interviews with CDDs (n = 5) -Two rounds of FGDs parents (n-FGDs = 20)^(a)^	-SAC: 80.7% (Round 1), 81.4% (Round 2) (PZQ and ALB, registers)	6/10
		SAC	PZQ, ALB	CWT		Rural (5 villages)			-SAC: 80.2% (Round 1), 81.9% (Round 2) (PZQ and ALB, registers)	
Massa et al., 2009b [49]	Tanzania	SAC	PZQ, ALB	SBT	Quantitative: Cross-sectional	Rural (5 villages)	1 year	-Quantitative review of registers (CWT: n = 4,570; SBT: n = 2,469)	-SAC: 80.7% (Round 1), 81.4% (Round 2) (PZQ and ALB, registers)	6/9
		SAC	PZQ, ALB	CWT		Rural (5 villages)			-SAC: 80.2% (Round 1), 81.9% (Round 2) (PZQ and ALB, registers)	
Muhumuza et al., 2013 [50]	Uganda	SAC	PZQ	SBT	Quantitative: Cross-sectional	Rural (12 schools)	1 month	-Surveys with school-enrolled children, grades 4 to 6 (age: 10–14) (n = 1,010)	-SAC: 28.5% (95% CI: 22.9% - 33.6%) (PZQ, survey)	8/9
Muhumuza et al., 2014 [51]	Uganda	SAC	PZQ	SBT	Quantitative: Randomized Control Trial	Rural (12 schools)	3 months	-Surveys with school-enrolled children, grades 4 to 6 (age: 10–14) (n-treatment = 595, n-control = 689)	-SAC—treatment: 93.9% (95%CI: 91.7% - 95.7%) (PZQ, survey) -SAC—control: 78.7% SAC (95% CI: 75.4%-81.7%) (PZQ, survey)	8/14
Muhumuza et al., 2015a [52]	Uganda	SAC	PZQ	SBT	Quantitative: Cohort study	Rural (12 schools)	1 year	-Surveys with school enrolled children, grades 4 to 6 (age: 10–140). (Year 1, n-treatment = 595, n-control = 689; Year 2, n-treatment = 536; n-control = 536).	-SAC—treatment: 78.0% (95%CI: 74.1–81.6%) (PZQ, survey) -SAC–control: 70.4% (95%CI: 66.7% - 73.9%) (PZQ, survey)	7/9
Muhumuza et al., 2015b [53]	Uganda	SAC	PZQ	SBT	Qualitative	Rural (12 schools)	2 months	-FGD with school-enrolled children, grades 4 to 6 (age: 10–14) (n-FGD = 24)^(b)^ -Interviews with teachers (n = 12) -Interviews with health assistants (n = 2) -Interview with District Vector Control Officer (n = 1).	-SAC: 48.9% (95%CI: 44.4%– 53.4%) (PZQ, survey results reported elsewhere) [54]	7/10
Ndyomugyenyi and Kabatereine, 2003 [55]	Uganda	SAC	PZQ, IVM, Mebendazole	SBT	Mixed-methods	Rural (4 schools)	3 months	-Interviews with teachers (n = 4) -Surveys with SAC (n-SAC = 284)	-SAC 79% (PZQ and MBD, survey)	9/15
				CWT		Rural (55 communities)		-Interviews with community leaders (n = 55) -Interviews with teachers (n = 55) -Surveys with SAC (n = 502) -FGD with key informants (local level decision makers) (n-FGD = 6)^(b)^	-SAC: 85% (PZQ and MBD, survey)	
Odhiambo et al., 2016 [56]	Kenya	SAC	PZQ	SBT (Year 1–2)	Qualitative	Urban (9 health sub-units)	3 years	-Three rounds of FGD with CHWs (n-CHWs = 18)	-Not reported	6/10
		SAC Adults	PZQ	CWT (Year 3)					-Not reported	
Omedo et al., 2012 [57]	Kenya	SAC Adults	PZQ	CWT	Qualitative	Rural (75 villages)	1 month	-FGD with CHWs (n-FGD = 8, n-CHWs = 65)	-Over 75% (no further details reported)	5/10
Omedo et al., 2014 [58]	Kenya	SAC Adults	PZQ	CWT	Qualitative	Rural (75 villages)	1 month	-FGD with CHWs (n-FG = 8, n-CHWs = 53)	-Not reported	5/10
Parker, Allen and Hastings, 2008 [20]	Uganda	SAC Adults	PZQ, ALB	CWT	Qualitative	Urban and Rural (Panyimur town, trading centre, landing sites, Pandara village and one Waseko town)	5 months	-Unstructured Interviews with village elders, healers, health staff, teachers, beneficiaries (n = 300) -Interviews with adults in main treatment area (10% of treated HHs)^(b)^ -Interviews with treated adults in neighbouring villages (Pandara, n = 20; Waseko, n = 58), -Interviews with CDDs in neighbouring villages (n = 4) -Interviews with health practitioners in neighbouring sites (Waseko: n = 4). -FGD with treated adults in neighbouring sites (Waseko, n-adults = 58)	-Adults: 66.8% (PZQ and ALB, registers)	7/10
Parker and Allen, 2011 [19]	Uganda	SAC Adults	PZQ, ALB, IVM	CWT	Qualitative	Urban and Rural (Panyimur: trading centre, 15 villages; Moyo and Adjumani: landing sites, 7 villages; Busia: 14 villages)	a. Panyimur: 3 months b. Moyo and Adjumani: 3 months c. Busia: 2 months	a. Panyimur: -Participatory observation (trading centre) -Interviews with key informants (n = 50) -FGD with key informants (n-FGD = 10)^(b)^ -Semi-structured interviews with adults (n = 595) -Review of records for adults (n = 50) b. Adjumani district: -Semi-structured interviews with adults (n = 103) -Open ended interviews with key informants^(a)^ c. Moyo district (2008) -Semi-structured interviews with adults (n = 72) -Open ended interviews with key informants^(a)^ d. Busia district (2009) -Participatory observation (landing sites) -Open-ended interviews with key informants^(b)^ -Semi-structured interviews: 10% of adults in 14 villages^(b)^	a. Panyimur^(d)^ -Adults–2004: 70% (PZQ, interviews) -Adults–2005: 37% (PZQ interviews) -Adults–2007: 40% (PZQ, interviews) b. Moyo: -Adults-2005: 32% (PZQ interviews) -Adults-2006: 39% (PZQ interviews) -Adults-2007: 41% (PZQ interviews) -Adults-2008: 55% (PZQ, interviews) c. Adjumani: -Adults-2005: 29%: (PZQ, interviews) -Adults-2006: 59% (PZQ, interviews) -Adults-2007: 50% (PZQ, interviews) -Adults-2008: 74% (PZQ, interviews) d. Busia -Adults-2008: 67% (PZQ, interviews) -Adults-2009: 64% (, PZQ, interviews)	7/10
Pearson, 2016 [59]	Uganda	SAC Adults	PZQ, ALB, IVM	CWT	Qualitative^(g)^	Rural (multiple small fishing landing sites)	1 year	-Participant observation -Water-contact observation exercises (n-sites = 10) -Group discussions^(f)^ -Semi-structured interviews^(b)^ -Unstructured interviews with key-informants in landing sites and health services^(b)^ -Parasitological survey with adults (n = 383)	-Adults: 56% (PZQ, survey)	5/10
Randjelovic et al., 2015 [60]	South Africa	SAC	PZQ	SBT	Quantitative: Cross-sectional	Urban and Rural (43 schools)	5 months	-Quantitative review of treatment registers in primary, intermediate and high schools (pupils’ age: 3 to 15) (n = 24,005)	-SAC: 44.3% (PZQ, registers)	5/9
Rilkoff et al., 2013 [61]	Uganda	SAC Adults	PZQ, ALB, IVM, Zithromax	CWT	Qualitative	Rural (8 villages)	3 months	-FGD with community leaders (n-FGD = 8, n-leaders = 30) -FGD with CDDs (n-FGD = 8, n-CDDs = 17) -FGD with adult men (n-FGD = 8, n-men = 68)) -FGD with adult pregnant women (n-FGD = 8, n-women = 91) -FGD with breastfeeding women (n = FGD = 8, n-women = 100) -FGD with adolescent men (n-FGD = 8, n-men = 73) -FGD with adolescent females (n = FGD = 8, n-women = 64). -Interviews with programme supervisors (n = 2) -Participatory observation of MDAs.	-Adults: 82.9% (treatment registers); 42.2% (village household register)^(d)^, ^(h)^	7/10
Sanya et al., 2017 [62]	Uganda	SAC Adults	PZQ, ALB	CWT	Qualitative	Rural (6 villages)	4 months	-Interviews with adults (n = 36) -Interviews with community leaders (n = 12). -FGD with adults (n-FGD = 12, n-adults = 60)	-SAC and adults—intensive intervention group (Mean uptake in 13 villages): 63% (PZQ, registers) -SAC and adults–standard intervention group ((Mean uptake in 13 villages): 56% (PZQ, registers) (estimates reported elsewhere) [63]	5/10
Tuhebwe et al., 2015 [64]	Uganda	SAC Adults	PZQ	CWT	Mixed-methods	Rural (15 villages)	1 month	-Surveys with adults (n = 615) -Interviews with key informants (n = 5)	-Adults: 44.7% (95% CI: 40.8%– 48.7%) (PZQ, survey)	10/15

(a) Maximum score possible varied according to study design: qualitative (10), quantitative–cross-sectional (9), quantitative–cohort (9), randomised controlled trial (14), mixed-methods (15).

(b) Number of participating individuals not reported.

(c) Information not reported, details obtained from Ministry of Health’s guidelines for MDAs.

(d) Calculated by authors based on data reported in publication.

(e) Study declared having used qualitative methods as well. However, their contribution to results and discussion is negligible or unrelated to study subject. The paper is hence classified as of quantitative nature.

(f) Targeted population unspecified.

(g) Study declared having used quantitative methods. However, their contribution to results and discussion is negligible or unrelated to study subject. The paper is hence classified as of qualitative nature.

(h) Type of drugs undefined.

**Table 3:** Number of publications reporting on determinants of treatment uptake, according to target populations and delivery-strategies (Complete references: S3 Table).

Themes	Categories	Target Populations		Type of MDA		
		SAC (n = 13)	SAC & Adults (n = 17)	SBT (n = 8)	CWT (n = 13)	MIXED (n = 9)
**INDIVIDUAL LEVEL**						
**Demographic Characteristics**	Age	3	3	1	2	3
	Sex	1	1	1	1	-
**Material wellbeing**	Access to food	5	5	5	4	1
	Livelihoods	-	10	-	9	1
	School enrolment	2	1	-	-	3
**Drug-related factors**	Fear of side effects	6	12	4	9	5
	Side effects indicate PZQ works	1	3	-	2	2
	Size, smell and taste of tablets	1	5	1	2	3
**Knowledge of SCH**	Biomedical knowledge of SCH	4	7	3	5	3
	Awareness of being at risk	3	2	2	1	2
**Beliefs and attitudes regarding the nature of SCH**	SCH not a major health concern	1	3	1	2	1
	Traditional explanations of SCH	2	3	1	2	2
**Knowledge of MDAs**	Access to operational information	3	6	1	5	3
	Understanding of MDAs’ rationale	3	8	2	6	2
	Unclear drug-administration procedure	1	3	1	2	1
**Beliefs and attitudes regarding MDAs’ effectiveness**	Perceived health benefits	1	8	-	7	2
	Perceived competence of distributors	2	4	1	3	2
	Religious beliefs	-	2	-	1	1
**INTERPERSONAL LEVEL**						
**Negative rumours of MDAs**	Deaths and severe health consequences	1	6	1	4	2
	Mistrust towards government	1	4	1	3	1
**Social influence**	Adults’ influence on SAC	1	2	1	1	1
	Peer-pressure	1	1	1	1	-
	Local social networks	-	2	-	1	1
**ORGANISATIONAL LEVEL**						
**Health systems’ support**		-	4	-	2	2
**COMMUNITY LEVEL**						
**Community engagement**	Community engagement	3	3		3	3
	Leaders’ perceptions of health benefits	2	-	-	-	2
**Socio-cultural trajectories**	Social cohesion	-	4	-	4	-
	Gender values	-	4	-	3	1
	Past public health campaigns	-	2	-	2	-
**Geographical features**	Setting’s size	3	4	1	3	3
	Migration patterns	-	2	-	2	-
**POLICY LEVEL**						
**Sensitisation**	Content	5	5	3	4	3
	Training for distributors	1	4	1	4	-
	Variety of dissemination sources	2	4	1	2	3
	Length of time	1	1	1	-	1
**Incentives for distributors**	Material incentives / compensation for distributors	3	8	1	6	4
	Immaterial rewards for distributors	3	4	-	4	3
**Design of MDA operations**	Distribution strategies	5	2	-	2	5
	Organisational structure	-	2	-	1	1

## Results

### State of the literature

Selected publications provided a solid combination of complementary evidence. A comparable number of qualitative and quantitative studies (fourteen and thirteen, respectively), in addition to three mixed-methods studies, were included. Two thirds (n = 20) comprised cross-sectional or short-term qualitative assessments (≤1 year). The remaining ten publications reported on six long-term assessments of MDAs (≥3 years) [16,19,20,44,50–53,56,59]. Concerning the type of MDA examined, eight publications studied school-based MDAs, thirteen reviewed community-wide interventions, and nine contrasted mixed distribution approaches.

The quality assessment exercise revealed that selected publications yielded a rather sound body of evidence. Most publications (n = 23) obtained a 60% quality score or higher, according to the maximum points attainable for their respective design. Only two publications obtained a score of less than 50%.

**Fig 2:**
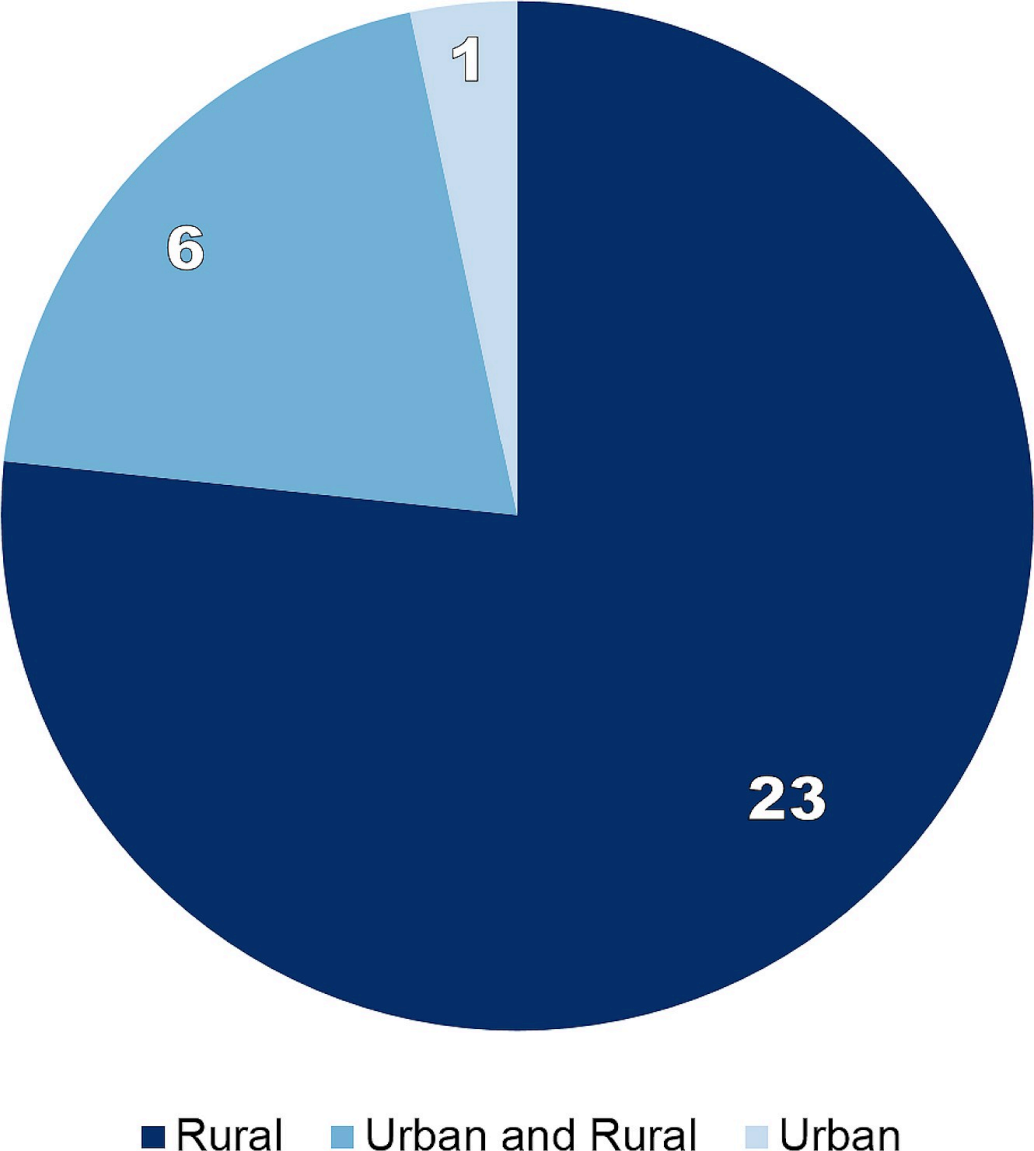
Number of studies by type of setting examined.

**Fig 3:**
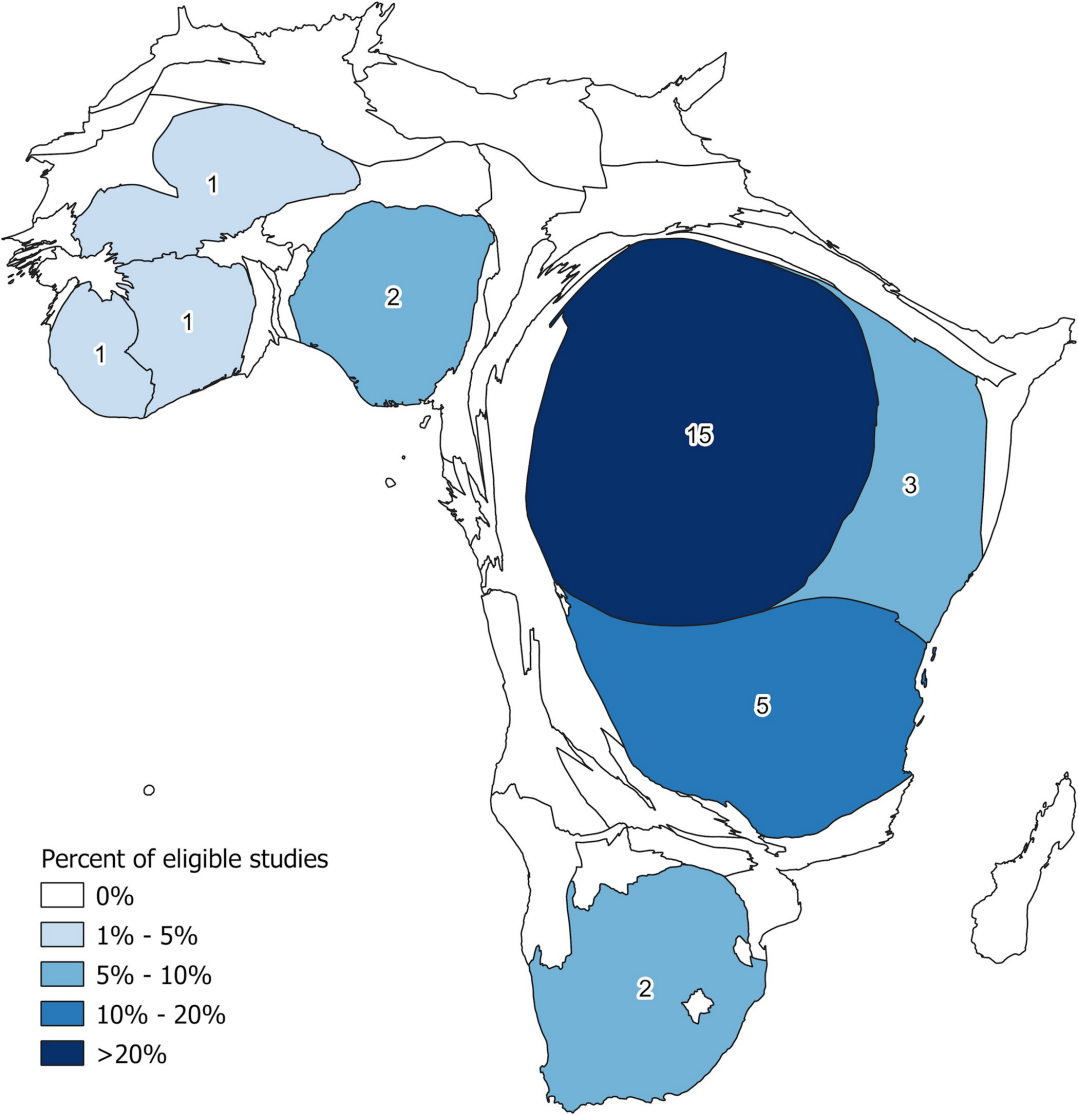
Density equalising cartogram of Africa, with countries’ size re-scaled proportionally to the number of publications contributed to the review (Elaboration: authors, spatial data source: Database of Global Administrative Areas).

Two forms of geographical bias were observed. First, selected studies were mostly based in rural areas: Twenty two exclusively focused on such settings, seven contrasted urban and rural MDAs, whilst only one solely studied urban populations (Fig 2). Second, between Uganda (n = 15), Tanzania (n = 5), and Kenya (n = 3), 76.7% of all selected publications were based in East Africa. Studies from West Africa totalled five publications: Nigeria (n = 2), alongside Liberia, Mali, and Cote d’Ivoire (one each). Two studies were based in South Africa and none in the Central African region (Fig 3).

**Fig 4:**
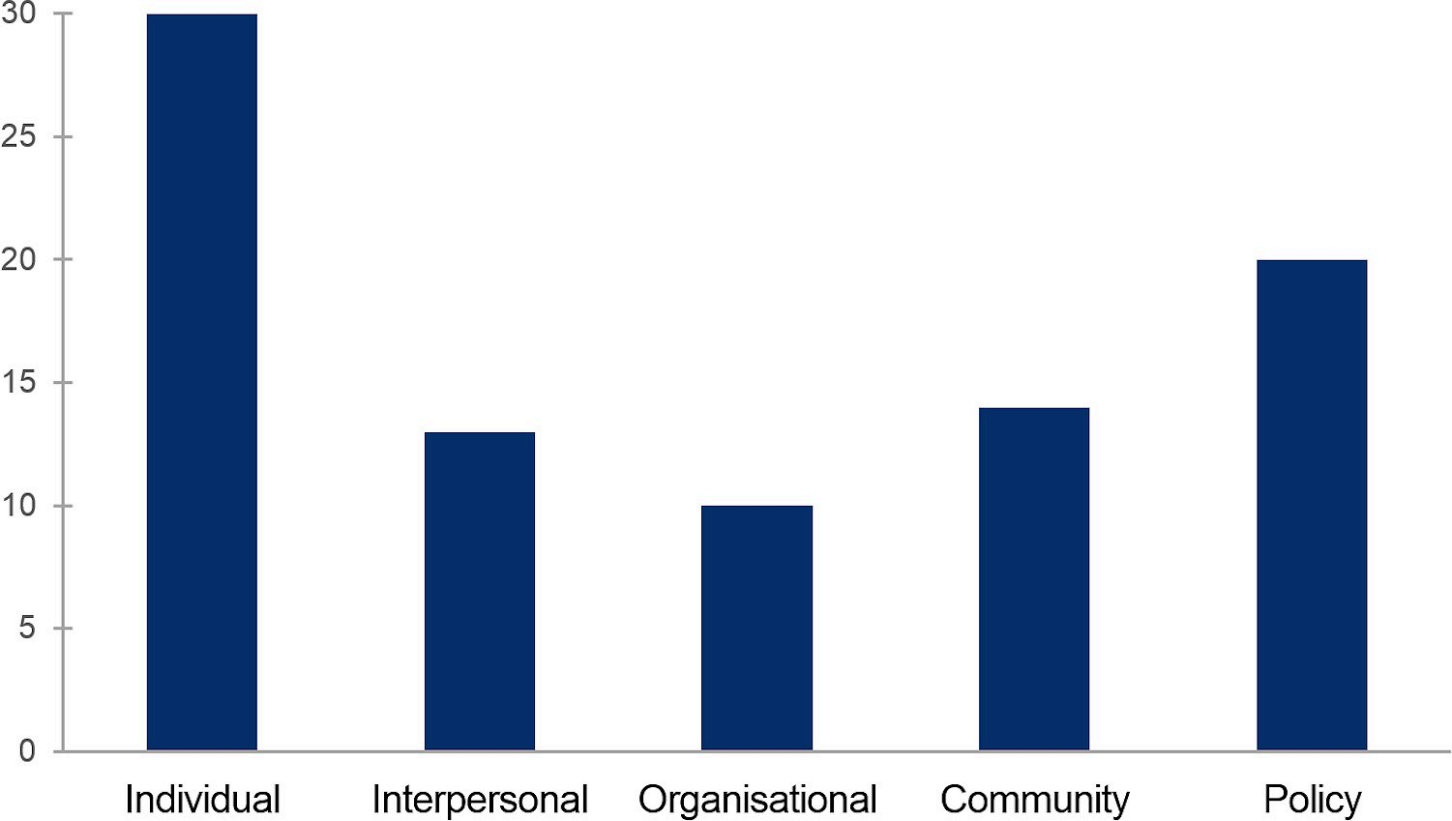
Number of publications reporting evidence for each level of analysis, as per the socio-ecological framework of health behaviour.

A review of the evidence reported in selected studies indicated that not all of the levels of analysis proposed were studied to the same extent (Fig 4). All thirty articles discussed conditioning factors associated to individuals (e.g., socio-demographic characteristics, knowledge and attitudes), whilst twenty discussed programme-level (policy) issues (e.g., drug-distribution or sensitisation approaches). In contrast, examinations of intermediate (mediating) factors were less common, particularly organisational issues (ten publications).

### Individual level of analysis

#### Demographic characteristics

Examinations of the relationship between age and treatment coverage during MDAs rendered unclear results. Thirteen studies examined this association [37,39–41,43,47,49,51,52,54,55,60,64], of which six reported a significant effect. Direct comparisons between SAC and adults [37,43,47,60] found that the latter were less likely to partake of MDAs. The reasoning provided was that parents and teachers were more able to control children’s behaviour as compared to grown-ups, who enjoyed greater independent decision-making capacity. Chami et al. [41], however, found that the older the resident, the more likely they would be offered treatment during a CWT campaign in Uganda. Comparisons between different SAC age-groups, in turn, reported no clear trends either. Whilst studies of a SBT intervention in South Africa [60] and one CWT in Nigeria [47] observed greater participation among young children (<10) rather than elder SAC, examinations of SBT activities in Nigeria and Uganda found the opposite trend [47,55].

Concerning sex, from twelve studies that examined its direct effects on uptake [39–41,43,46,47,51,52,54,60,61,64], only two found a significant association: one in South Africa [60], examining SAC, and another in Uganda [61], comprising SAC and adults. In both cases female beneficiaries appeared to be more likely to participate of MDAs. The main explanation provided was that men tended to spend more time outdoors and to travel, which limited their access to information and presence in town during MDAs. Rilkoff et al [61], in Uganda, also noted that boys and men were more likely to disregard distributors’ advice. We must emphasise, however, that gender was frequently mentioned as an indirect influencing factor (See S3 Table).

#### Material wellbeing

Coverage assessments based on assets-based indicators of wealth provided inconclusive evidence of a relationship. A study in Uganda found that those who owned a well-built home [40] were more likely to receive treatment. However, a second survey in the same district a few months later [41] found no association between this indicator and being offered treatment or ingesting the drugs. Similarly, whilst latrine ownership was a non-significant predictor in the first study, it was later found to be associated with being offered treatment (but not with treatment compliance). Apart from these two studies conducted, other coverage assessments using comparable predictors, such as dwellings’ sanitation infrastructure [50,64] or construction materials [37], failed to identify a significant association.

Wellbeing indicators related to access to food, livelihoods, and school-enrolment provided clearer trends. Food concerns were mentioned by a third of all studies [19,39,44,51–53,56,57,61]. The consensual explanation was that, since consuming Praziquantel on an empty stomach increases the chances of experiencing side effects, such as vomiting, dizziness or stomach cramps [65], some rejected treatment unless they managed to eat satisfactorily in advance. Noticeably, a study in Uganda [53] reported that distributors themselves chose not to treat children on empty stomachs to avoid dealing with health complications. The importance of food access was somehow measured by a randomised controlled trial with SAC in Uganda [51]. It was then reported that coverage in those schools that provided a snack prior during MDAs reached 93.9% coverage (95%CI: 91.7% - 95.7%), significantly higher than in the control group: 78.7% (95% CI: 75.4%-81.7%). Moreover, a one-year follow-up study showed that that once food support was stopped, coverage was similar for both the intervention (78.0%, 95%CI: 74.1–81.6%) and the control groups (70.4%, 95%CI: 66.7% - 73.9%) [52].

Beneficiaries’ livelihoods, in turn, were found to condition adults' participation mainly in community-based MDAs [19,41–43,56,57,59,61,62,64]. Fishing was the most singled out occupation to put people at risk of missing out on treatment [19,41,56,59,62,64]. This happened because fisherfolk regularly spent long hours either fishing or travelling to remote catchment areas or markets, hence remaining uninformed of MDA activities or being absent during treatment days. Other mobile occupations had similar effects, such as trading [19,59,61], truck driving [61], and herding [43]. Studies in Kenya [57] and Cote d’Ivoire [42], in addition, highlighted that absenteeism could equally affect settled farmers during labour-intensive periods of the agricultural calendar, such as the planting season.

The impact of school-enrolment was examined by studies in Tanzania and Uganda. In the first country, Massa et al. [48,49] found that school-enrolled SAC were more likely to partake of MDAs than non-enrolled SAC during two rounds of SBT (Round 1: 82.1% against 59.2%; Round 2: 83.0% against 56.6%) but no differences were found in settings using CWT approaches (Round 1: 80.3% vs 80.0%; round 2: 81.9% vs 82.9%). In Uganda, Adriko et al. [37] found that non-enrolled SAC were less likely to receive treatment even when SBT and CWT strategies were used simultaneously.

#### Drug-related factors

Fear of side effects was the most salient barrier to treatment uptake identified, mentioned by two thirds of all selected publications [16,19,20,36,41,42,44–46,48,53,54,56–59,62]. The extent and manner in which it affected participation, however, varied according to context. Hastings, for instance, reported that such fears escalated rapidly in Tanzania, so much that riots arose once they were accompanied by rumours of deaths among treated children [44] and, in Uganda, Muhumuza and colleagues found that in an MDA with low coverage among SAC (28%), 72% of those who refused treatment said it was due to fears of side effects [50]. In contrast, examinations of CWT with poor coverage results (<50%) in Cote d’Ivoire [42] and Uganda [64] found that fears of side effects only played a minor role. In the first one, only 10.6% of non-compliers mentioned it as their reason to avoid treatment, whilst the Uganda study found no association between awareness of side effects or fears of dying after treatment with the odds of swallowing Praziquantel tablets. Strikingly, four studies based in Uganda [16,19,59,62] and one in Nigeria [36] reported that side effects generated favourable views of MDAs since SAC and adults interpreted them as evidence that people were indeed infected with schistosomiasis and that the drugs were killing the parasites.

Studies led by Muhumuza et al. [53] and Fleming et al. [16] in Uganda, in addition, reported that SAC disliked Praziquantel because of its pungent smell and large size. Noticeably, studies in Cote d’Ivoire [42], Kenya [56], Tanzania [45], and Uganda [64] observed similar dislikes among adults.

#### Knowledge of schistosomiasis

A third of the selected studies reported that having good knowledge of symptoms, transmission cycle, or preventive measures for schistosomiasis enhanced participation in MDAs [16,20,36,42,46,53,54,56,58,64]. The mechanisms through which knowledge shaped participation, however, were not similarly construed across those publications. A first set of studies [16,36,50,56,64] suggested that people who were aware of the disease’s properties became motivated to participate in MDAs to treat their symptoms and improve their health. Others emphasised that knowledge of sources of transmission increased local awareness of being at risk of contracting schistosomiasis and so prompted residents to ingest Praziquantel to prevent contagion [36,46,52,62]. In-depth qualitative examinations offered a more nuanced view. Studies led by Muhumuza [53] and Parker [20] in Uganda, for instance, described that access to information on the disease could lead to negative responses. There, beneficiaries were aware of the disease and its health impacts but, since their understandings of schistosomiasis were mixed with those of intestinal parasites, stomach infections, and consumption of untreated water, local expectations of treatment were more akin to that for intestinal worms, which do not generate side effects. Once the latter were observed, local rejection of treatment increased.

#### Beliefs about schistosomiasis

The effects of local beliefs on treatment coverage for schistosomiasis were examined mainly by qualitative studies. One factor observed in South Africa [62] and Uganda [16,46,59] was that those who did not consider schistosomiasis a severe health condition, either because it did not kill or it was ‘part of life’, were less likely to participate in MDAs. Studies led by Fleming [16] and Sanya [63], in Uganda, reported that such views could be an unintended consequence of successful MDAs. Then, prevalence of heavy intensity infections decreased significantly after various rounds of treatment, making some to consider the disease as manageable.

In turn, studies in Nigeria [36], South Africa [46] and Uganda [16,20,59] noted that beneficiaries who defined schistosomiasis in terms of witchcraft or other traditional beliefs were more inclined to rely on healers rather than on PC. The nature of these beliefs varied across settings. In Nigeria, Adeneye et al. [36] reported that some believed that urinating in a T-junction of a road was the origin of the infection; Lothe and colleaguess work with Zulu communities in South Africa [46] found that some considered schistosomiasis an African-specific disease that could only be cured only through ‘African remedies’; whilst Parker et al. [20] reported that some considered people’s envy as the cause of infection, which should be treated by witchdoctors. Studies in Nigeria [36] and Uganda [59], however, noted that these traditional beliefs could co-exist with positive views of PC treatment. They found that some considered healers a secondary treatment alternatives in case they could not access ‘modern’ medicines.

#### Knowledge of MDAs

Adequate access to information containing the basic operational details of MDAs (i.e., name of disease, dates, and location) enabled participation [38,39,41,47,55–58,64]. For example, in Kenya, Omedo and colleagues [57] described that parents could not ensure children’s school attendance or household members’ presence at home during MDAs unless they received this kind of information. Moreover, even if people were present, some rejected treatment because they considered they could not make an informed decision. Limited access to operational information, in turn, sometimes resulted in people confounding PC for schistosomiasis with other campaigns, such as those for sexually-transmitted diseases [56,57], malaria [56], or Ebola [38]. Barriers afflicting the latter could then be projected onto MDAs for schistosomiasis. In Kenya, for instance, some requested mosquito nets to partake in MDAs [56] whilst in Liberia fears of MDAs were linked to fears of Ebola vaccination activities [38].

Other types of knowledge of MDA that affected participation concerned people’s understanding of the rationale of MDA as well as of the drug-administration procedure used. Ten studies [19,20,41,44,48,53,56,57,59,61] described that residents customarily questioned why MDA campaigns treated entire populations without prior medical examination or why continuous treatment was required. Qualitative examinations in Uganda [19,20,59] showed these concerns emerged from beneficiaries’ first-hand interactions with health services, in which people typically received treatment only after showing symptoms and being examined by a health professional. Residents hence questioned the clinical soundness of MDAs since most children showed no symptoms and none were tested. Likewise, reports from Uganda [16,19,20] and Tanzania [44] showed that parents sometimes doubted that height was a sufficient indicator to estimate treatment dosage. These doubts equally emerged from parent’s experiences of medical services, which commonly enquired about children’s weight and age. Many thus believed that side effects were the consequence of overdoses, since distributors failed to gather complete anthropometric information.

#### Beliefs and attitudes regarding PC

Different types of perceptions of MDAs were found to condition participation. A first observation was that if informants perceived that other people’s health improved after the intake of Praziquantel, they would be inclined to participate [16,19,20,36,38,57,59,62,64]. For instance, Omedo and colleagues, in Kenya [57], noted that many residents adopted a ‘wait and see’ attitude, so that demand for PC grew over time, as people observed that others indeed got cured. None of the studies examining SBT activities reported this issue affected coverage in this more controlled environment.

A second factor concerned people’s perception of the competence of drug-distributors. Support for MDAs was found to be limited when residents considered that distributors were not fully-trained health professionals and hence lacked knowledge about the disease, medicines, or side effects [41,44,48,56,57,61]. Rilkoff and colleagues in Uganda [61], for instance, reported that beneficiaries requested that ‘experts’ should be brought over since distributors were not able to provide adequate explanations about the nature of the disease or preventive measures, whilst, in Kenya, a study led by Omedo [57] reported that some refused treatment because distributors lacked formal accreditation, like a diploma or certificate.

A final point on the matter, observed in Kenya [56,57], was that rejection of MDAs sometimes resulted from a disbelief in modern medicine by religious groups, which considered praying as the sole way of healing.

### Interpersonal level of analysis

At this level, the most commonly mentioned conditioning factor concerned rumours that spread through residents’ social networks, which mostly comprised stories of fatalities or severe health consequences following MDAs [16,19,20,44,56,57,61]. In Kenya, some believed that Praziquantel produced cancer [57]; in Uganda, Fleming described that fears of treatment increased after beneficiaries shared the story of a woman who supposedly died from severe diarrhoea after treatment [16]; whilst parents violently interrupted MDAs in local schools in Tanzania when unconfirmed news of deaths among children became widespread [44]. These rumours were commonly embedded in a broader context of mistrust towards national governments. Most studies describing these events equally mentioned that residents found rumours credible because they considered that their governments were willing to conspire with foreign agencies to reduce Africa’s population [19,20,44,56,57].

A second body of work discussed social influence issues. Adults’ authority over SAC was highlighted by three studies based in Uganda: Muhumuza and colleagues [50] found that SAC were more likely to receive treatment if they believed that their teachers supported MDAs, whilst Fleming [16] and Rilkoff [61] reported, respectively, that parent’s fears of treatment were reproduced among children and that boys were likely to be influenced by male adults’ negative attitudes, irrespective of kinship connection, since they sought their acceptance when socialising (e.g., during fishing). Peer-pressure was mentioned by a couple of studies. Qualitative studies in South Africa [46] and Uganda [61] found that children were more likely to participate if they perceived that their peers did the same. Lothe et al.’s work in South Africa also highlighted that children sometimes mocked those who participated in MDAs because of the association of schistosomiasis with sexually-transmitted diseases and extreme poverty [46].

Residents’ access to extended and well-established community relations also appeared to enable access to treatment. In Uganda, Adriko and colleagues [37] found that those who resided in their villages for less than five years were less likely to be treated, whilst Chami et al. [41] found that those who were at the centre of numerous social connections were most likely to be offered Praziquantel during CWT campaigns.

### Organisational level of analysis

A single organisational issue was salient in the literature. It concerned the support available to drug-distributors when they were somehow integrated into public health systems. In Kenya [56,57], the existing primary health care infrastructure was used to recruit community health workers to act as drug-distributors. As a result, those based in urban areas had a well-developed supervisory structure, with community health extension officers conducting supervision activities for free, whilst those in urban as well as rural settings were able to access direct support from health centres to manage severe cases of side effects. Fleming and colleagues [16], in turn, described how district health officers in Uganda gradually decided to rely more on health workers since they constituted a more stable and reliable workforce than community volunteers, whose dedication decreased due to the lack of incentives. The importance of accessing health systems’ support was further illustrated a study in Mali [43], which found that the odds of receiving treatment increased by 1.4 (95%CI: 1.16–1.66) when health workers visited targeted communities alongside community drug-distributors.

### Community level of analysis

#### Community engagement

Diverse forms of community participation in MDA activities reportedly facilitated treatment uptake [19,36,43,48,55,58]. Various benefits were noted. First, authorities’ involvement projected their own prestige towards MDAs. In Kenya, for instance, chiefs’ participation in sensitisation activities enhanced local trust [58] whilst, in Uganda, Parker and Allen [19] reported that the support from the catholic church boosted participation in districts where it was influential. Second, engagement from local leaders widened opportunities for dissemination like in Kenya, where preachers supporting PC activities shared information with their followers [58]. Third, community support sometimes allowed distributors to access additional resources for their work, such as bicycles in Tanzania [48] and notebooks for registration as well as helpers to control children and fetch water in Mali [43]. Studies in Nigeria [36] and Tanzania [48] reported that authorities’ perception that Praziquantel was effective in curing schistosomiasis was key to obtain their support.

#### Socio-cultural trajectories

Community-level socio-cultural factors were found to affect the implementation of MDAs, mainly CWT activities. First, studies led by Chami [40,41] and Parker [20] in Uganda identified that social cohesion and exclusion issues impacted on coverage. The first one, observed that the likelihood of having PC was significantly higher among those who belonged to the village majority tribe or the majority religion, whilst the latter reported that conflicts between long-term residents and recent migrants in a frontier district affected the programme’s capacity to effectively mobilise the community for MDAs. Likewise, Dabo and colleagues reported that, in Mali [43], those who belonged to a minority ethnic group were less likely to partake of CWT campaigns given their residing in remote areas and mobile livelihoods (herding).

Second, gender values affected drug-distributors’ performance during CWT. Noticeably, these effect were pervasive. Odhiambo et al. in Kenya [56] mentioned that female drug-distributors could feel insecure when working in the evenings, particularly when visiting remote areas. Also in Kenya, Omedo and colleagues reported that female distributors [57] faced problems at home, even abuse, since their long working hours affected their domestic duties and husbands perceived these activities as non-profitable. In turn, Parker and colleagues described how in Uganda [19] female distributors struggled to convince older men to accept treatment given their disparity in social status. Dabo et al. in Mali [43], furthermore, observed that residents were not keen to select women as distributors.

Two studies in Uganda [19,59], in turn, showed that communities’ past experiences with public health campaigns affected uptake. Specifically, they described how previous successful experiences of interventions against sleeping sickness as well as humanitarian aid enabled a positive reception towards MDAs.

#### Geographical features

Population size and distribution constituted a first geographical factor conditioning MDA’s reach [37,41,43,47,48,60,61]. Mafe and colleagues in Nigeria [47] found that large dispersed populations (n>2000) were characterised by low coverage rates despite using different drug-distribution platforms (central distribution: 39.5% and school-based: 26.3%). Likewise, a study in Mali [43] reported that the odds of receiving treatment increased by 2.27 (95%CI: 1.74–2.97) in villages where the number of residents a distributor needed to treat was 150 or lower, independent of whether central-distribution or house-by-house strategies were used. In the context of SBT, a study in South Africa [60] found that, compared to schools with fewer than 350 students, the odds of taking Praziquantel were lower for those with 350 to 700 (AOR: 0.48, 95% CI: 0.40–0.58) and over 700 pupils (AOR: 0.47 AOR, 95% CI 0.39–0.56). These challenges were sometimes exacerbated by the terrain. Odhiambo et al. [56], for instance, described that distributors in Kenya were unable to reach households in areas vulnerable to natural hazards.

CWT strategies, in addition, appeared sensitive to migration issues. Descriptions of frontier settlements in Uganda [19,59] showed that distributors struggled to identify the population eligible for treatment due to the constant presence of transient visitors, resulting in many non-residents receiving tablets.

### Policy level

#### Sensitisation

Programme-level decisions regarding sensitisation were found to affect treatment coverage through different means. First, the content of those campaigns sometimes left unaddressed topics of interest to beneficiaries [19,20,36,44,48,51,53,56–58]. In the context of MDAs that failed to reach 75% coverage in Uganda, Muhumuza [53] and Parker and colleagues [19,20] reported that whilst beneficiaries had received general information about the disease and operational details of MDAs, little information had been provided on why people needed treatment despite not showing symptoms or why annual treatment was required. Hastings’ study of riots in Tanzania [44] showed that rejection of PC was partly explained by a teachers-led sensitisation campaign that did not explain the reasons for distributing Praziquantel without a diagnosis, side effects, or the dosage-estimation process using SAC’s weight.

Second, various studies noted that drug-distributors’ capacity to address beneficiaries’ doubts was limited due to information gaps in their training [19,20,53,61,64]. Uganda-based studies [19,20,53] reported that distributors were mostly taught how to administer drugs, receiving little information on the aetiology of the disease, the rationale behind MDAs, or side effects. Likewise, a study about integrated MDAs in the same country [61] found that distributors were unable to conduct sensitisation activities or explain treatment guidelines to pregnant women due to incomplete training, resulting in substantive confusion among resident about eligibility as well as poor coverage outcomes. Noticeably, a different study in Uganda [64] reported that the absence of trained staff was sometimes widespread, with seven of the fifteen villages examined lacking trained distributors.

The operational features of sensitisation campaigns, third, also affected treatment uptake. One aspect was the use of multiple means of communication [16,44,56–58,64]. In the failed MDA studied by Hastings in Tanzania [44], the author described how schools were singled out as the sole responsible for informing communities, whilst leaving aside community-level associations and district-level public organisations. In contrast, depictions of successful campaigns reported the use of multiple means of information, including community gatherings, funerals, radio campaigns, and road shows to reach urban populations in Kenya [56] or radio campaigns alongside leaflets and road-shows as well as communications with district-level public officials alongside community associations (e.g., women’s groups) to reach rural residents from the same country [58]. Another issue was the length of time allocated to sensitisation activities. In Tanzania, Hastings [44] related that school meetings were held just one or two days before the MDAs, resulting in poor attendance, whilst Fleming noted that, in Uganda [16], health education was often provided just on the day of treatment.

#### Incentives for distributors

The provision of material incentives or compensation to distributors was a central programmatic challenge raised by the literature, given its potential effects on performance and attrition [16,19,20,36,43,48,53,56,57,61,64]. The time-labour demands associated to MDAs played a central role. Parker and colleagues in Uganda [20] described that drug-distributors’ expressed their displeasure at the lack of remuneration for their work in CWT campaigns because they had to walk for long hours for several days, which affected their income earning and food production activities. Even though programmes sometimes provided incentives, time-labour demands were often perceived as more costly. In the context of a CWT in Nigeria, which provided lunch allowances and T-shirts, a study [56] found that distributors complained that they were insufficient because distributors had to skip more than one meal a day and needed to change clothes regularly. Likewise, a study in Uganda [53] noted that, whilst teachers received $2USD and a T-shirt, many considered that these did not account for all the hours dedicated to MDAs, which apart from training, sensitisation, and drug-distribution included the tedious task of filling in registration forms.

Some studies emphasised that distributors’ focus on immaterial benefits, like public recognition for their work, an enhanced social status for being a health-practitioner, and their personal satisfaction for helping their communities [19,20,36,41,48,55,57], could counteract the lack of material incentives. Whether such benefits outweighed the needs for material compensation in the long-term, however, appeared uncertain. Studies in Uganda [19,20] and Kenya [57] noted that although distributors acknowledged the importance of such rewards, demands for material compensation tended to increase over time.

#### Design of MDA operations

Multiple publications noted that programme’s selection of drug-delivery strategies conditioned coverage outcomes [36,42,43,47–49,55]. Five studies conducted comparisons between CWT and SBT approaches in comparable settings [36,47–49,55], with all suggesting that door-to-door visits tended to render better outcomes. In Nigeria, Adeneye [36] and Mafe [47] and their colleagues contrasted coverage rates and beneficiaries’ satisfaction between central distribution strategies, door-to-door distribution and SBT, with the latter obtaining the lowest coverage (28.5% and 49.2%, respectively) and door-to-door distribution the highest (72.2% and 77.2%, respectively). Qualitative studies, in turn, indicated that beneficiaries found receiving the drugs at home more convenient, given their work obligations and SAC’s irregular school attendance. Likewise, in Tanzania, Massa et al. [48,49] reported that whilst beneficiaries were satisfied with school-based and door-to-door strategies alike, they preferred the latter to ensure MDAs reached non-enrolled SAC. Registers confirmed that home visits yielded higher coverage among non-enrolled SAC (Round 1: 80% (CWT) vs 59.2% (SBT); Round 2: 82.9% (CWT) vs 56.6% (SBT)). Comparisons between door-to-door and central distribution strategies in Mali [43] and Cote d’Ivoire [42], in addition, observed that the first one attained higher coverage.

Unclear staff structures were also found to affect MDA operations. A study in Uganda [16] described how sub-district health officials were not involved in other intervention activities beyond storing drugs and managing patients, which limited the support health services provided for distribution activities. In turn, a study in Kenya [57] described how the use of two different staff to deal with beneficiaries (enumerators to register eligible residents and health workers to distribute drugs), resulted in confusion and conflict, since they believed they were usurping each other’s roles.

## Discussion

**Fig 5:**
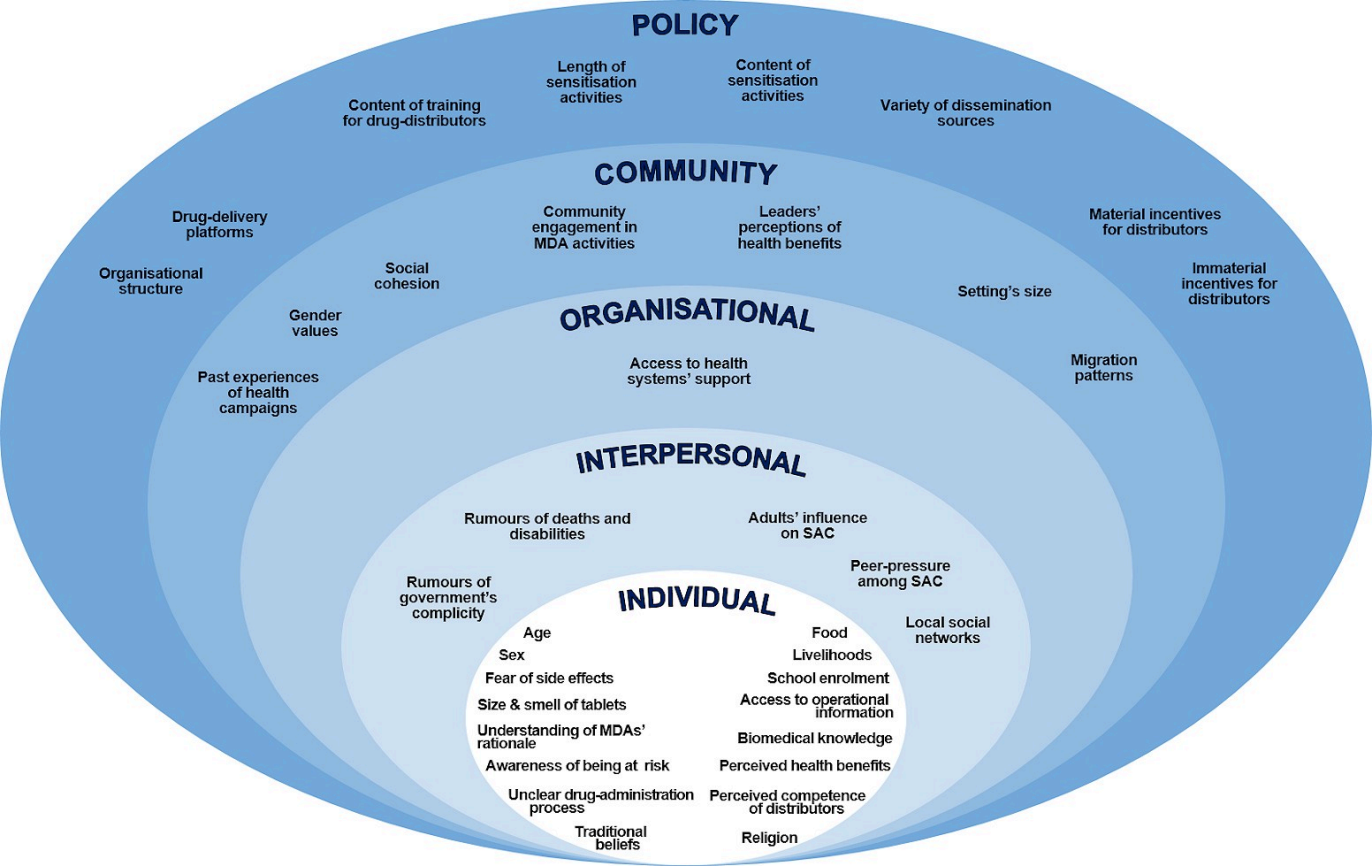
Conditioning factors shaping treatment coverage in SSA according to the socio-ecological model of health behaviour.

A good understanding of the factors that condition the uptake of PC for schistosomiasis in SSA can help achieving sustained high coverage outcomes in the future. To inform relevant programmatic recommendations, this review has provided a comprehensive examination of empirical evidence on the subject from peer-reviewed publications published between January 2002 and 2019, drawing on the analytical underpinnings of the socio-ecological framework of health behaviour (Fig 5) [24,25].

Emerging recommendations, however, should be presented with caveats. One limitation is the geographical bias observed in selected publications. Three quarters of them were based in Uganda, Tanzania, and Kenya and a similar proportion solely examined rural settlements. Previous systematic reviews on social research for schistosomiasis control [17,18,21,23] showed a similar over-representation of the East African region and a limited focus on urban–rural comparisons, indicating that our search results mostly reflected the current state of academic production on the subject.

Another limitation concerns the preference in the selected literature for examining individual- and policy-level determinants rather than those operating at intermediate levels (i.e., interpersonal, organisational, or community). Comparable reviews on treatment uptake for NTDs [21,66] reported similar trends, indicating that such gaps are common in the NTDs literature as well.

An additional limitation relates to this study’s use of a thematic approach to systematise the evidence from studies differing in size, location, and methodology. To ensure a coherent systematisation process, the team adopted quality control measures for the data extraction, coding, and thematic integration stages, including working in pairs and organising multiple review rounds of the coding framework. We are aware, however, that much of this decision-making process was subjective and that other data-integration strategies may have been used.

### Addressing individual-level determinants

Four discussion points emerge from this review’s findings on individual-level determinants. One concerns equity in MDAs, a major consideration given current commitments to ensure universal access to NTD services, independent of age, gender or socio-economic condition [26]. Our results rendered a mixed picture. A minority of publications that examined differences in coverage according to age (six out of thirteen) [37,41,43,47,55,60], sex [60,61] (two out of twelve), and assets ownership (two out of five) [40,41] reported significant results. Other wellbeing indicators, however, provided more direct evidence of hurdles limiting access to PC among the worse-off, including food availability [19,39,44,51–53,56,57,61], school enrolment [37,48,49], and economic obligations [19,41–43,56,57,59,61,62,64]. These results highlight the importance of developing strong monitoring and evaluation systems for MDAs, that routinely gather disaggregated equity data using validated indicators. Presently, apart from age, sex and school-enrolment, the collection of treatment data for other categories remains limited, including poverty or disability [15]. Further efforts are needed to develop cost-effective tools that facilitate large scale data gathering of this kind, with sufficient depth to identify and explain the barriers and contextual enablers that affect marginalised populations. Emerging frameworks indicate that targeted collection of qualitative data, alongside routine surveys, are suited for such ‘leave no one behind’ assessments [67].

A second concern relates to individuals’ negative response to the properties of Praziquantel tablets, expressed in fears of side effects [16,19,20,36,41,42,44–46,48,53,54,56–59,62] and dislike of the drug’s size and smell [16,42,45,53,56,64]. The saliency of the first barrier is of interest since it can be explained by two interacting factors. One the one hand, side effects are common after Praziquantel intake. A meta-analysis on tolerability among SAC showed an incidence rate of 56% (95%CI: 45.2%-66.4%) for any side effect and 31.1% for abdominal pain (95%CI: 22.0–39.0) [68]. On the other, programme’s recommendation that beneficiaries eat prior treatment as a preventive measure clashes with the reality of SSA, where many households regularly endure food shortages [20,44]. Addressing those barriers may require a combination of strategies. Provision of snacks or porridge during MDAs were found to encourage participation [48,51]. However, this strategy has significant financial implications for national campaigns. Other studies found that information campaigns that explained side effects, highlighting their temporary nature, and provided medical support for complications minimised treatment rejection [56,66]. Programmes would hence benefit from planning MDAs along those lines, particularly in their early years, since residual apprehension may persist if side effects are mismanaged then [19,20]. The best-case scenario, however, would be that the drug’s formulation is improved so that its properties no longer generate negative responses. The Paediatric Praziquantel Consortium, which is currently trialling a small, orally dispersible tablet with an acceptable taste, can be a first step in that direction [69].

A third element concerns knowledge issues. Our review found that awareness of the symptoms, consequences, and transmission cycle of schistosomiasis prompted some to receive treatment to improve their health or prevent contagion [16,20,36,42,46,53,54,56,58,64]. Likewise, it was reported that limited understanding of the rationale of MDAs [19,20,41,44,48,53,56,57,59,61] and drug-administration procedures [16,19,20,44] could generate doubts and fears. As proposed elsewhere [21,23], these findings imply that programmes should expand and intensify their health education efforts. Our findings, however, also showed that access to ‘correct’ information not always led to positive outcomes. Instead, ‘correct’ and ‘incorrect’ knowledge commonly coexisted, were mixed and re-interpreted. For example, awareness that schistosomiasis was a parasite led some to believe that treatment was similar to that used for helminthiasis [20,53], knowledge of side effects made some to believe they indicated that tablets worked [16,19,36,59], whilst first-hand experiences with medical services made some parents to question the medical soundness of treating children without a diagnosis [19,20,41,44,48,53,56–59,61] or assessing their weight and age [16,19,20,44]. It is thus be advisable that, when designing sensitisation campaigns, programmes move away from narrow interpretations of knowledge that mostly concern with whether informants can replicate official information, such as Knowledge-Attitudes-and-Practice frameworks [23]. A more nuanced, broader, examination of knowledge that pays due attention to the local socio-cultural context and beneficiaries’ views, without dismissing them as mere ‘misconceptions’, is essential. Otherwise, programmes may conduct campaigns that disempower beneficiaries, who are told to be in the wrong, whilst distributing messages that are not tailored to local audiences, potentially sowing confusion [70,71].

This review also evidenced the need for distinguishing between knowledge and attitudinal barriers to inform health education campaigns. As established in the health behaviour literature, knowledge effects on behaviour are customarily mediated by attitudes [25,72]. Indeed, as reported, people’s awareness of the disease did not imply that they would necessarily assume that PC was required. Different decisions were made depending on beneficiaries’ perceptions of the seriousness of the disease [16,46,59,62] or its nature as a traditional illness [16,20,36,46,59]. Perceptions about the effectiveness of drugs [16,19,20,36,38,57,59,62,64], the value of praying as treatment [56,57], or distributors’ competence [41,44,48,56,57,61] further conditioned people’s actions. These observations question the presumption that intensifying the provision of bio-medical information may be sufficient to improve treatment uptake [21,23]. Studies on anti-vaccination attitudes and climate-change denial have shown that people with such views are not characterised by their lack of access to information but by their biased selection of evidence that reinforces their worldviews, even if weak [73,74]. It is hence recommended that sensitisation activities are informed by health behaviour frameworks to device strategies to deal with the primary socio-cultural factors promoting treatment rejection, like partially acknowledging them (e.g., reaching out to healers) or establishing workarounds to minimise their impact (e.g., emphasising affective messages rather than facts-based arguments) [72,73]. Further research is needed on behaviour change for schistosomiasis control to provide more specific recommendations on the subject.

### Addressing interpersonal-level determinants

It is possible to assert that underneath factors identified at this level, including negative rumours [16,19,20,44,56,57,61] and adults’ capacity to direct SAC’s participation [16,50,61], lies the issue of trust. As described, the credibility of rumours of fatalities after MDAs were often linked to mistrust of national governments [19,20,44,56,57], whilst adults’ position as figures of authority and esteem enabled them to shape SAC’s behaviour. Engaging local influential actors in MDA activities can be essential given their observed capacity to influence the flow of information and resources circulating through local social networks during MDAs [37,41]. This can take different shapes. The selection of distributors from among community members with good reputation or credentials, for instance, can enhance treatment uptake, if they are found trustable by beneficiaries [66]. Ensuring that they are well-trained and so able to absolve people’s doubts could be key to provide a counter-narrative to local rumours. Likewise, gathered evidence showed that further credibility can be attained when local leaders play an active role during MDAs [19,36,58] given their local prestige.

An additional element concerns the effects of peer pressure on treatment uptake among SAC, who sometimes avoided treatment to gain social acceptance [46,61]. These observations may indicate the usefulness of reaching out towards young beneficiaries themselves to promote, through their networks, a more receptive environment for MDAs. Recent children-oriented interventions have attempted to achieve this through learning-by-playing activities [75,76]. Further evidence, however, is needed to confirm their effectiveness.

### Addressing organisational-level determinants

Some studies highlighted that when health workers were responsible for MDAs, rather than volunteers, they benefitted from greater clinical supervision and access to resources to deal with health complications, which enhanced local trust [16,43,56,57]. These findings, alongside those reported in reviews assessing drug-distributors’ motivating factors and support systems [17,18,22], indicate the value of promoting a greater degree of integration of front-line staff into health systems to secure greater and more stable material support. Deciding on which integration framework is best suited to NTD programmes, however, remains unclear. A review of experiences of integration of community health workers into health systems showed that no single framework is preferable [77]. Instead, different levels of integration across programme components may be required to better adapt to existing policy frameworks and available resources. Further research is thus needed. Nevertheless, it is recommended that frontline staff preserve community connectedness despite their new roles as public officials, once integrated, to ensure local trust, public accountability, and community support [17,77].

### Addressing community-level determinants

Evidence on community-level determinants showcased the need for programmes to invest in community engagement activities to enhance treatment outcomes. Noticeably, evidence of benefits from such an approach was found across different MDA activities. As mentioned earlier, involving local leaders when setting up MDAs in its first years could help build a trustable reputation [19,36,58]; their engagement in drug-delivery activities helped some distributors to access additional material and human resources, improving their performance [43,48]; and mobilising community leaders and organisations helped to widen the reach of sensitisation campaigns [36,58]. Furthermore, there is indicative evidence from community-based behaviour change interventions for schistosomiasis control that engaging residents in monitoring and evaluation activities can contribute towards better outcomes [78,79].

This review’s findings that communities’ social cohesion [20,40,41,43], gender values [43,56,57], history [19,59], and geography [37,41,43,47,48,56,60,61] could affect coverage, particularly for CWT approaches, further indicates the importance of embracing community-based approaches to programming. Social researchers have previously highlighted the need for anthropological accounts to tailor MDAs to the local context [19,20,44]. However, most programmes lack the time, money, and expertise to carry them out. The adoption of participatory planning approaches may provide a viable alternative [80]. Currently, control programmes are expected to engage with local authorities and civic organisations to gather population data as well as identify available resources, risk factors, and sensitisation opportunities [10]. The development of participatory protocols that widen the array of organisations and leaders included, to give representation to marginalised groups like women, ethnic minorities, and people with disabilities, as well as expand the subjects discussed, to include identifying and proposing responses to community-level determinants, would be of useful to adapt MDAs to end-beneficiaries’ needs.

### Addressing policy-level determinants

Three programmatic considerations emerge from policy-level evidence. One pertains sensitisation and training activities. This review found that information campaigns frequently prioritised disseminating operational details of PC campaigns (e.g., dates) rather than discussing complex issues, like side effects or MDA’s rationale [19,20,36,44,48,51,53,56–58]. Whilst individual-level information showed that access to operational information enhances participation [38,39,41,47,55–58,64], it also showed that those more complex topics constitute key decision-making considerations for beneficiaries [19,20,41,44,48,53,56,57,59,61]. The effects of information gaps during sensitisation could be further compounded by similar gaps during drug-distributors’ training, which was sometimes found to be mostly concerned with teaching drug-administration procedures [19,20,53]. The issues surrounding training are noteworthy given distributors’ critical role in ensuring the success of MDAs. A recent systematic review of WHO’s policy, operations, and training guidelines for drug-distributors confirmed that training programmes commonly overlook health education topics [22]. Furthermore, to the best of authors’ knowledge, there is a noticeable paucity of publications discussing evaluations of training programmes for distributors in the NTDs literature. It is thus recommended that programmes adopt a more comprehensive agenda for their sensitisation and training activities and that resources are allocated to evaluate their impact.

A second consideration relates to incentives for drug-distributors, given their impact on performance and attrition [16,19,20,36,43,48,53,56,57,61,64]. Reported findings indicate the need for a twin-pronged approach to this challenge. On the one hand, there is a need to enhance the material support available. One strategy, already discussed, is pursuing greater integration of front line staff into health systems to secure a stable access to resources [17,18]. Another is that provision of financial incentives should consider the opportunity costs and out of pocket expenses that distributors incur, to reduce the gap between distributor’s needs and actual rewards [17]. Ensuring that distributors’ roles are well-defined to avoid uncertainty over responsibilities may also contribute to reduce perceptions of high opportunity costs [22,57]. On the other hand, programmes may promote the intrinsic incentives that distributors value [19,20,36,41,48,55,57]. Strategies may include connecting distributors with local authorities through community engagement activities, to enhance their perception of an improved social status; making known to residents that they are unpaid, to increase local support and public gratitude; or providing some form of accreditation or public recognition by health services.

A final issue concerns drug-distribution platforms. Evidence gathered from studies comparing sites of analogous socioeconomic and epidemiological condition in this review found that door-to-door CWT approaches rendered better outcomes than school-based MDAs [36,47–49,55]. This differs from Burnim et al.’s review [21], which compared treatment outcomes for divergent strategies implemented in different countries to conclude that the combination of CWT and SBT may be more effective. The authors of this review, however, consider that making direct inferences from outcome measures to inform programme-level decision-making is problematic. MDAs are complex interventions, with multiple mediating factors operating at the community, organisational, and intrapersonal levels. Further information is thus needed to ascertain the pathways through which decisions on drug-delivery platforms generated observed results. It might be beneficial that programmes conduct a structured examination of trade-offs between distribution strategies to make an informed decision, in which treatment coverage constitutes another, albeit key, consideration. Trade-offs identified in this review included contrasting time-labour demands, vulnerability to community-level determinants (i.e., socio-cultural, governance, and geographic), and distributors’ social status and influence (teachers compared to volunteers).

## Conclusion

This review has comprehensively mapped out the barriers and facilitators conditioning the uptake of PC for schistosomiasis in SSA. Gathered evidence showcased the presence of multiple determinants operating simultaneously across all levels of analysis, from the individual- to the policy-level. Moreover, given the limited information obtained concerning organisational-level factors as well as urban and Central and Southern African settings, the reported list of determinants may yet be completed. In addition, the study demonstrated the interdependent nature of determinants operating within similar levels of influence, such as the effect of attitudinal barriers on knowledge at the individual-level, and across different ones, as in the case of individual-level knowledge issues vis-à-vis programme-level decisions on sensitisation and training activities.

The need to understand MDAs as complex interventions thus becomes apparent [81]. Programme-level decisions are being constantly reshaped by mediating (individual and collective) actors and their socio-cultural environment, which interact with each other, further impacting on outcomes. The provision of easy-to-implement solutions, correspondingly, remains elusive. The dynamics of treatment uptake is likely to vary from country to country, so that one-size-fits-all solutions are unlikely to be attainable; key components of potential solutions, such as behaviour change, community engagement, and health systems strengthening strategies, constitute long-term complex processes themselves; whilst, the implementation of remedial measures to address specific barriers require careful planning to avoid generating negative knock-on effects. Ensuring support for context-based transdisciplinary examinations of the pathways through which determinants impact on beneficiaries’ uptake behaviour appears central. System-based approaches, which examine linkages, interactions, and feedback mechanisms between system’s components and their environment may provide opportunities for moving this research agenda forward [82].

## Supporting information

S1 File Prisma checklist. (DOC)

S1 Table Quality assessment criteria used to assess selected publications. (DOCX)

S2 Table Complete data extraction sheet. (DOCX)

S3 Table Complete list of studies’ contributions to thematic synthesis according to target populations and types of MDA. (DOCX)
