# Supplementary material for: Factors affecting the uptake of preventive chemotherapy treatment for schistosomiasis in Sub-Saharan Africa: A systematic review
Source: PLoS Negl Trop Dis. 2021 Jan 19;15(1):e0009017. doi: 10.1371/journal.pntd.0009017 (PMC7846123; doi:10.1371/journal.pntd.0009017)
Supplement: S1 Table — (DOCX) [file pntd.0009017.s002.docx]

**S1 Table. Quality Assessment Criteria**

| QUALITATIVE | CROSS-SECTIONAL | RANDOMISED CONTROL TRIAL | MIXED – METHODS |
| --- | --- | --- | --- |
| **1.** Is there congruity between the stated philosophical perspective (i.e., theory) and the research methodology?  **2.** Is there congruity between the research methodology and the research question or objectives?  **3.** Is there congruity between the research methodology and the methods used to collect data?  **4.** Is there congruity between the research methodology and the representation and analysis of data?  **5.** Is there congruity between the research methodology and the interpretation of results?  **6.** Is there a statement locating the researcher culturally or theoretically?  **7.** Is the influence of the researcher on the research, and vice-­‐ versa, addressed?  **8.** Are participants, and their voices, adequately represented?  **9.** Is the research ethical according to current criteria or, for recent studies, and is there evidence of ethical approval by an appropriate body?  **10.** Do the conclusions drawn in the research report flow from the analysis, or interpretation, of the data? | **1** Is there congruity between the research methodology and the research question or objectives?  **2.** Were the criteria for inclusion in the sample clearly defined?  **3.** Were the study subjects and the setting described in detail?  **4.** Were objective, standard criteria used for measurement of explanatory factors?  **5.** Were confounding factors identified?  **6.** Were strategies to deal with confounding factors stated?  **7.** Were the outcomes measured in a valid and reliable way?  **8.** Was appropriate statistical analysis used?  **9.*** Do the conclusions drawn in the research report flow from the analysis, or interpretation, of the data? | **1** Is there congruity between the research methodology and the research question or objectives?  **2.** Was true randomization used for assignment of participants to treatment groups?  **3.** Was allocation to treatment groups concealed?  **4.** Were treatment groups similar at the baseline?  **5.** Were participants blind to treatment assignment?  **6.** Were those delivering treatment blind to treatment assignment?  **7.** Were outcomes assessors blind to treatment assignment?  **8.** Were treatments groups treated identically other than the intervention of interest?  **9.** Was follow-up complete, and if not, were strategies to address incomplete follow-up utilized?  **10.** Were participants analysed in the groups to which they were randomized?  **11.** Were outcomes measured in the same way for treatment groups?  **12.** Were outcomes measured in a reliable way?  **13.** Was appropriate statistical analysis used?  **14.** Was the trial design appropriate, and any deviations from the standard RCT design accounted for in the conduct and analysis of the trial? | 1. Is there an adequate rationale for using a mixed-methods design to address the research question?  2. Are the different components of the study effectively integrated to answer the research question?  3. Are the outputs of the integration of qualitative and quantitative components adequately interpreted?  4. Are divergences and inconsistencies between quantitative and qualitative results adequately addressed?  5. Do the conclusions drawn in the research report flow from the analysis, or interpretation, of the data?  **FOR QUALITATIVE COMPONENT:**  **6.** Is there congruity between the research methodology and the representation and analysis of data?  **7.** Is there congruity between the research methodology and the interpretation of results?  **8.** Is there a statement locating the researcher culturally or theoretically?  **9.** Is the influence of the researcher on the research, and vice-­‐ versa, addressed?  **10.** Are participants, and their voices, adequately represented?  **FOR QUANTITATIVE COMPONENTS:**  **11.** Were the criteria for inclusion in the sample clearly defined?  **12.** Were objective, standard criteria used for measurement of explanatory factors?  **13.** Were confounding factors identified?  **14.** Were the outcomes measured in a valid and reliable way?  **15.** Was appropriate statistical analysis used? |
