# Supplementary material for: Factors affecting the uptake of preventive chemotherapy treatment for schistosomiasis in Sub-Saharan Africa: A systematic review
Source: PLoS Negl Trop Dis. 2021 Jan 19;15(1):e0009017. doi: 10.1371/journal.pntd.0009017 (PMC7846123; doi:10.1371/journal.pntd.0009017)
Supplement: S2 Table — (DOCX) [file pntd.0009017.s003.docx]

**S2 Table. Complete data extraction sheet**

| **Ref.** | **Type of data** | **Level of Analysis** | | | | |
| --- | --- | --- | --- | --- | --- | --- |
|  |  | **Individual** | **Interpersonal** | **Organisational** | **Community** | **Institutional** |
| Adeneye et al., 2007 [1] | **Qualitative** | **Fear of side effects:**   - A number of children experienced mild-side effects (e.g., stomach pain). However, parents were informed beforehand of their potential occurrence. They were not surprised, and many chose not to report them since they knew effects would be temporary.   **Side effects indicate PZQ works:**   - Some considered that side effects signalled that the drug was effective.   **Biomedical knowledge of SCH:**   - There was a high level of knowledge of the presence of SCH in the area, as well as of the source of transmission of SCH (infected water), and key symptoms. This resulted from previous MDA campaigns, eventually improving the attitude of parents and the children to taking preventive measures to curb the disease’s incidence.   **Awareness of being at risk:**   - There is high level of knowledge that rivers and streams constitute sources of transmission. This prompted parents to recommend preventive measures.   **Traditional beliefs:**   - There is still constant use of herbal medicines. However, they are complementary to modern medicines. People rely on them due to cost. If possible, many prefer modern treatments. - Some residents explain the infection in terms of superstition (e.g., urinating in T-junction of road) and opt for treatment through herbal medicines   **Perceived health benefits:**   - Children and parents alike considered PZQ tablets effective. Residents noticed a reduction in cases of children with blood in their urine. |  |  | **Community support**   - Leaders assisted with mobilisation activities to create awareness on the availability of drugs. - Some parents collaborated with the MDA campaign, keeping children in order or providing water. This was perceived by distributors to facilitated distribution.   **Leaders’ perceived health benefits**   - Village leaders support MDAs as they perceive that the drugs are effective and the number of SCH cases has decreased in their communities.   **Seasonality**   - Some preferred MDAs to take place during periods of abundance (harvest season of when fish sales improve). Some preferred the wet season because they considered SCH could be more prevalent at the time. | **Sensitisation: content**   - Health education campaign emphasising sources of transmission increased biomedical knowledge of SCH among locals as well as the adoption of preventive measures among residents.   **Incentives / compensation for distributors**   - A few distributors unwilling to carry on with their work cited that they were too busy and the lack of incentives for their work as reasons for leaving the programme.   **Intrinsic rewards for distributors**   - Distributors perceived their role as a way of serving their communities and increasing their knowledge of the disease, whilst improving their social status   **Distribution Strategy**   - Residents considered that home-by-home distribution was more convenient than PHC due to obligations / occupation and irregular school attendance. - In practice, implementation of strategies varied from village to village. PHC villages visited children at school and home in addition to waiting for visits at health centre. Among the two CWT villages, one used home by home visits whilst the other used a central location first and then visited who missed treatment at home. One SBT village visited children at home after distribution at school. |
| Adriko et al., 2018 [2] | **Quantitative: significant** | **Age (**reference: SAC)**:**   - Pre-SAC : AOR=0.02, 95%CI (0.01-0.05), p<0.05 - 15-19: AOR=0.51, 95%CI (0.32-0.83), p<0.05 - 20-29: AOR=0.42, 95%CI (0.23-0.77), p<0.05 - 30-39: AOR=0.53, 95%CI (0.28-1.03), p>0.05 - 40-49, AOR=0.50, 95%CI (0.21-1.01), p>0.05 - 50+ years: AOR=0.36, 95%CI (0.17-0.78), p<0.05   **School enrolment** (reference: not-enrolled):   - Enrolled (any grade): AOR=6.17, 95% CI (3.58-10.64), p<0.05   **Preventive behaviour:**   - Mosquito-net ownership (Yes / No): AOR=1.44, 95%CI(0.96-2.15), p=0.05 | **Length of residence** (reference: 5 to 9 years)   - Short-term (<5 years): AOR=0.58, 95%CI(0.37-0.91), p<0.05 - Long-term (10+): AOR=0.93, 95%CI(0.64-1.35), p>0.05 |  | **Setting’s size** (reference: Bugoto A (n>1000))   - Bugoto B (<1000): AOR=3.39, 95% CI(2.15-5.35), p<0.05 |  |
|  | **Quantitative: non-significant** | **NOTE:** Predictors excluded from final multivariate model through stepwise regression, statistical tests associated to each specific variable unreported. | | | | |
|  |  | **Sex** (p>0.05)  **Livelihoods** (p>0.05)  **Religion** (p>0.05)  **Household’s assets / infrastructure**   - Bed ownership (p>0.05) - House structure (materials of floor, walls and roof) (p>0.05) - Water sources (for drinking, bathing and washing) - Latrine structure (materials of floor, walls and roof) (p>0.05) |  |  |  |  |
| Bogus et al, 2016 [3] | **Quantitative** ^(a), (b), (c)^ | **NOTE:** Descriptive statistics from non-random sample. Only frequencies over 10% considered. | | | | |
|  |  | **Access to operational information:**   - 17% of respondents considered it was necessary to reassure residents MDA is not for Ebola. - 24% of interviewed leaders considered necessary to reassure residents MDA was the same as before. - 80% of informants recommended that communities should be provided more information on the MDAs prior treatment, emphasising it is not connected to Ebola vaccination.   **Perceived health benefits:**   - 90.1% of respondents agreed “the medicine was good, it improved health”. |  |  |  |  |
| Bukindu et al., 2016 [4] | **Quantitative** (Significant) | **Access to food** (reference: No)   - Food provision on treatment day (Yes): AOR=25.25, 95% CI (5.28–120.49), p=0.001   **Access to operational information** (reference: No):   - Received information on MDA prior treatment (Yes): AOR=14.24, 95% CI (3.23–62.72), p=0.001**.** |  |  |  |  |
|  | Quantitative:  Non-significant | **Age** (Reference: 8-12)   - 13-18: AOR=0.63, 95% CI (0.15-2.56), p= 0.51.   **Sex** (reference: Female)   - Male: AOR=1.01, 95%CI (0.22-4.69), p=0.99.   **Biomedical knowledge** (Reference: No):   - Knowledge of parasites (Yes): AOR=20.84, 95%CI (0.11-6.23), p=0.86.   **Fear of side effects** (reference: not reported)   - Reported side effects: AOR=5.56, 95%CI (0.55-56.66), p=0.15. |  |  |  |  |
| Chami et al., 2016 [5] | Quantitative (Significant) | **Household’s assets / infrastructure**   - Home quality score: OR=1.18, 95% CI (1.07-1.31), p=0.002   **Preventive behaviour:**   - HH purifies drinking water (treat, filter, boil); OR=2.12, 95% CI (1.05–4.29), p=0.04 |  |  | **Community cohesion:**   - Household head in village majority tribe OR=2.11 (95%CI: 1.11-4.02), p=0.02 - Muslim head (Yes/No): OR=0.48, 95%CI (0.23-1.01), p=0.054 |  |
|  | Quantitative:  (Non-significant) | **Age**   - Age in years: OR=1.00, 95%CI (0.98-1.02), p=0.93   **Sex** (reference: Male)   - Female: OR=0.78, 95%CI (0.44-1.36), p=0.38.   **Education**   - Years of education: OR=0.93, 95%CI (0.84-1.02), p=0.12   **Occupation** (reference: No income occupation)**:**   - Fisherman or fishmonger: OR=1.46, 95%CI (0.37-5.79), p=0.59 - Business owner: OR=0.82, 95%CI (0.17-3.84), p=0.80 - Rice farmer OR=0.19, 95%CI (0.04-1.07), p=0.06 - Other farmer: OR=1.69, 95%CI (0.41-6.93), p=0.24 - School teacher: OR=1.15, 95%CI (0.10-13.33), p=0.91. - Other occupation: OR=1.69, 95%CI (0.41-6.93), p=0.47   **Social status:**   - Former or current chairman in household: OR=4.12 95%CI (0.85-19.98), p=0.08. - Other former or current village government member in household: OR=1.58, 95%CI (0.49-5.07), p=0.44.   **Household’s assets / infrastructure**   - Latrine ownership (No/Yes): OR=0.36, 95%CI (0.09-1.46), p=0.15. - Household seeks medical care from private clinics: OR=0.88 95%CI (0.46-1.69), p=0.70 | **Social networks:**   - Total years HH in village: OR=1, 95%CI (0.97-1.03), p=1.03. |  | **Setting’s size**   - Total HHs in village: OR=1.0, 95%CI (0.99-1.01), p=0.41.   **Epidemiological characteristics:**   - Village SCH prevalence: OR=1.02 (0.97-1.06), p=0.47 |  |
| Chami et al., 2017 [6] | **Qualitative** |  |  |  |  | **Intrinsic rewards for distributors**   - CDDs from three villages indicated they volunteered due to their wish to help others. - CDDs indicated they became drug distributors to gain preferential health treatment at government health centres and social status within their village. Some became village chairmen whilst many noted that being a CDD was a high-status position in their communities and they were even called doctors. Most noted that CDDs were able to interact with high-status people, such as health workers and authorities. |
|  | **Quantitative**^(a)^  (descriptive) | **NOTE:** Descriptive results for reasons for not taking drugs among non-compliers. Reasons with frequencies over 10% considered. | | | | |
|  |  | **Lack of food:**   - Lack of food or drink (10.77%)   **Fear of side effects**(*):   - Bad side effects (25.95%)   **Access to operational information**   - Did not know the purpose and benefits of the drugs (71.32%)   **Unclear rationale of treatment**(*):   - Had no symptoms (25.95%)   (*) Percentages calculated by authors using reported data. |  |  |  |  |
|  | **Quantitative** (Significant) | **NOTE:** Statistical models ran on two different outcome measures: coverage (defined in the study as the percentage of people offered treatment) and compliance (percentage of people who were offered treatment and ingested them). | | | | |
|  |  | **Age**   - Coverage: Age in years (degree model): AME=0.001, 95%CI (<0.001-0.002), p=0.03 - Coverage: Age in years (closeness model): AME=0.001, 95%CI (<0.001-0.002) p=0.03   **Perceived competence of distributors**   - Coverage: Trusts CMD for health advice (degree model); AME=0.070, 95%CI (0.040-0.104), p<0.001 - Coverage: Trusts CMD for health advice (closeness model); AME=0.048, 95%CI (0.038-0.102), p<0.001. - Compliance: Trusts CMD for health advice (degree model); AME: 0.048, 95% CI (0.019-0.077), p<0.001 - Compliance: Trusts CMD for health advice (closeness model); AME: 0.048, 95% CI (0.019 - 0.077), p<0.001   **Social status:**   - Coverage - religious, tribe, or clan leader or on the village council (degree model): AME=0.074, 95%CI (-0.022-0.125), p=0.01 - Coverage - religious, tribe, or clan leader or on the village council (closeness model): AME=0.070, 95%CI (-0.019-0.122), p=0.01   **Household’s assets / infrastructure**   - Coverage- Latrine ownership (No/Yes): AME=-0.081, 95%CI (-0.136-0.026), p<0.01. - Coverage- Latrine ownership (No/Yes): AME=-0.079, 95%CI (-0.133-0.024), p=0.01.   **Education:**   - Coverage - Highest level of education (degree model): AME=-0.009, 95% CI (-0.012 - -0.006), p<0.001 - Coverage - Highest level of education (closeness model): AME= -0.009, 95% CI(-0.012 - -0.006), p<0.001 - Compliance - Highest level of education (degree model): AME=-0.006, 95% CI (-0.009 - -0.003), p<0.001 - Compliance - Highest level of education (closeness model): AME= -0.006, 95% CI(-0.009 - -0.003), p<0.001   **Livelihoods (**reference: Not-economically active):   - Compliance - Other occupation (degree model): AME 0.027, 95% CI: 0.003-0.052, p=0.029 - Compliance - Other occupation (closeness model): AME 0.027, 95% CI: 0.003-0.052, p=0.030 | **Social networks:**   - Coverage- LN (Friendship degree + 1) [0-3.526]: AME=0.067, 95%CI (0.036-0.099), p<0.001 - Coverage - LN (Friendship degree + 1) [>3.526]: AME=0.303, 95%CI: (0.153-0.788), p=0.193 - Coverage – Friendship closeness centrality: AME=1.098, 95%CI (0.775 – 1.421), p<0.001 |  | **Community cohesion**   1. **Tribe**  - Compliance - Household head belongs to majority tribe (degree model): AME: 0.039, 95% CI (0.010-0.066), p=0.01 - Compliance - Household head belongs to majority tribe (closeness model): AME: 0.039, 95% CI (0.011-0.067), p=0.01  1. **Religion**  - Coverage - Muslim household head (degree model): AME=-0.049, 95%CI(-0.082- -0.015), p=0.01 - Coverage- - Muslim household head (closeness model): AME=-0.043, 95%CI(-0.077- -0.010), p=0.01   **Setting’s size**   - Coverage - Total homes in village (degree model): AME=-0.001, 95%CI (-0.001-<-0.001), p<0.001 - Coverage - Total homes in village (closeness model): AME=-0.001, 95%CI (-0.001-<-0.001), p<0.001 - Coverage – Village centre >50Km from Lake Victoria (degree model): AME=-0.160, 95%CI (-0.193- -0.127), p<0.001 - Coverage – Village centre >50Km from Lake Victoria (closeness model): AME=-0.001, 95%CI (-0.203- -0.138), p<0.001 |  |
|  | Quantitative:  (Non-significant) | **Age**  - Compliance: Age in years (degree model): AME<0.001, 95%CI (-0.001-0.001), p=0.55  - Compliance: Age in years (closeness model): AME<0.001, 95%CI (-0.001-0.001) p=0.54  **Sex** (reference: Male)   - Coverage - Female (degree model): AME=0.011, 95%CI (-0.005-0.027), p=0.18 - Coverage - Female (closeness model): AME=0.010, 95%CI (-0.005-0.026), p=0.20 - Compliance - Female (degree model): AME=0.011, 95%CI (-0.004-0.026), p=0.14 - Compliance -Female (closeness model): AME=0.011, 95%CI (-0.004-0.026), p=0.15   **Household’s assets / infrastructure**   - Compliance - No home latrine (degree): AME=0.001, 95%CI(-0.047-0.049), p=0.97 - Compliance - No home latrine (closeness): AME=0.001, 95%CI(-0.047-0.049), p=0.96 - Coverage - Home owned, not rented (degree model): AME=0.008, 95%C I(-0.039-0.055), p=0.74 - Coverage - Home owned, not rented (closeness model): AME=0.013, 95%C I(-0.033-0.060), p=0.58 - Compliance - Home owned, not rented (degree): AME=0.002, 95%CI(-0.043-0.047), p=0.93 - Compliance - Home owned, not rented (closeness): AME=0.002, 95%CI(-0.042-0.048), p=0.90 - Coverage - Home quality score (degree): AME=0.001, 95%CI(-0.003-0.006), p=0.56 - Coverage - Home quality score (closeness): AME=0.002, 95%CI(-0.003-0.006), p=0.44 - Compliance - Home quality score (degree): AME=-0.001, 95%CI(-0.005-0.004), p=.71 - Compliance - Home quality score (closeness): AME=-0.001, 95%CI(-0.005-0.004), p=0.72   **Livelihoods (**reference: Not-economically active):   - Coverage - Fisherman or fishmonger (degree model): AME-0.018, 95% CI (-0.055 – 0.019), p=0.34 - Coverage - Fisherman or fishmonger (closeness model): AME-0.015, 95% CI (-0.051 – 0.022), p=0.44 - Compliance - Fisherman or fishmonger (degree model): AME-0.002, 95% CI: -0.034 – 0.030, p=0.91 - Compliance - Fisherman or fishmonger (closeness model): AME-0.001, 95% CI: -0.033 – 0.030, p=0.93 - Coverage - Other occupation (degree model): AME=0.019, 95%CI(-0.007-0.045), p=0.159 - Coverage - Other occupation (closeness model): AME=0.017, 95% CI (-0.009-0.043), p=0.020   **Social status:**   - Compliance - Religious, tribe, or clan leader or on the village council (degree model): AME=0.018, 95%CI (-0.034-0.066), p=0.47 - Compliance - Religious, tribe, or clan leader or on the village council (closeness model): AME=0.018, 95%CI(-0.031-0.067), p=0.48 | **Connection with health staff:**   - Coverage: Close friends with CMD (degree model): AME=-0.0004, 95%CI (-0.04-0.04), p=0.84 - Coverage: Close friends with CMD (closeness model): AME=-0.017, 95%CI (-0.07-0.01), p=0.16 - Compliance: Close friends with CMD (degree model): AME=0.021, 95%CI (-0.01-0.04), p=0.22 - Compliance: Close friends with CMD (closeness model): AME=0.016, 95%CI (-0.02-0.05), p=0.36   **Social networks:**   - Friendship betweenness centrality (degree model): AME=-1.513, 95%CI (-3.616-0.590), p=0.158 - Friendship betweenness centrality (closeness model): AME=-1.328, 95%CI (-3.205-0.548), p=0.165 - Health betweenness centrality [0.0.268] (degree model): AME=0.420, 95%CI (-0.267-1.107), p=0.231 - Health betweenness centrality [0.0.268] (closeness model): AME=0.286, 95%CI (-0.390-0.963), p=0.407 - Health betweenness centrality [>0.268] (degree model): AME=0.520, 95%CI(-0.988-2.028), p=0.499 - Health betweenness centrality [>0.268] (degree model): AME=0.676, 95%CI(-0.831-2.182), p=379 |  | **Community cohesion**   1. **Tribe**  - Coverage - Household head belongs to majority tribe (degree model): AME=0.029, 95% CI (-0.002-0.060), p=0.07 - Coverage - Household head belongs to majority tribe (closeness model): AME=0.039, 95%CI (-0.002-0.060), p=0.06  1. **Religion**  - Compliance - Muslim household head (degree): AME=-0.021, 95%CI(-0.050-0.008), p=0.15 - Compliance - Muslim household head (closeness): AME=-0.020, 95%CI (-0.049-0.009), p=0.17 |  |
| Coulibaly et al., 2018 [7] | **Quantitative**  (descriptive)^(a), (c)^ | **NOTE:** Results come from cross-tabulations concerning reasons for not taking or disliking drugs by district. Reasons with frequencies over 10% are considered. | | | | |
|  |  | **Livelihoods**   - Busy with field activities as reasons for not taking treatment: 82.3% of non-compliers. - Dry season recommended as appropriate for treatment by 97.8% of all respondents.   **Fear of side effects**   - Adverse effects of previous treatment mentioned as reason for not taking treatment by 10.6% of non-compliers. - 45.0% of compliers disliked adverse effects of PZQ tablets.   **Size, smell and taste of tablets**   - Taste of PZQ tablets mentioned as reason for disliking treatment by 29.3% of compliers. - Size of tablets mentioned as reason for disliking treatment by 11.4% of compliers.   **Biomedical knowledge of SCH**   - Accepted treatment because of their knowledge of SCH / disease (56.4% of compliers). |  |  | **Setting’s size**   - Coverage in Moronou village (5000+ habs.): 52.3%, compared to coverage in Bigouin (cierca 1000 habs): 27.7% | **Distribution strategy:**   - House to house distribution preferred by 47.4% residents (n=405), as compared to central distribution in village (23.2%) or via health centres (29.4%). |
| Dabo et al., 2013 [8] | **Qualitative** | **Fear of side-effects**   - Some villagers said that they refused to take the drugs after hearing about their side effects: ‘It causes stomach aches, vomiting and dizziness for those who use it. Some were concerned they couldn’t work. |  |  | **Community support**   - Residents supported health education, community mobilisation and registration activities. Some also provided material support (e.g., notebooks and water)   **Gender values**   - Most CDDs elected by the community were men because most locals did not accept women in this position, particularly if young. | **Incentives / compensation for distributors:**   - CDDs invested substantive time to distribution. When residents were absent, they returned twice or three times or waited for the family’s return - Many had low morale because of their poor remuneration. - Some CDDs refused to carry out drug distribution because they prefer to be compensated for their services, as in other programmes (e.g., HIV). |
|  | **Quantitative** (Significant) | **Age (**reference: Adults)   - SAC: OR=1.13, 95%CI(1.01-1.26), p=0.03   **Livelihoods (**Reference: Fulani / Moorish, migrant cattle breeders)   - Bambara / Sarakolle, settled farmers: OR 1.34, 95% CI1.16-1.55, p<0.01. |  | **Access to health system’s support (**Reference: No health worker)   - Presence of health workers: OR 1.38, 95%CI (1.16-1.66), p<0.01 - Only 40% of the population reported that district directors of services visited communities and 60% that health workers only visited during a different specific campaign (e.g., immunisation and cholera). - CDDs propose and selected by communities. CHW were selected for supervision activities. | **Setting’s size** (reference: >=150 people per CDD)   - <150 hab. per CDD: OR=2.27, 95%CI(1.74-2.97), p<0.01.   **Social cohesion (**Reference: Fulani / Moorish, minority groups)   - Bambara / Sarakolle: OR 1.34, 95% CI1.16-1.55, p<0.01. | **Distribution Strategy**:   - Reference: central distribution: - House to house: OR=1.45, 95%CI (1.25-1.68), p<0.01. |
|  | **Quantitative** (Not significant) | **Sex** (reference: Male)  - Female: OR=0.97, 95%CI (0.87-1.08), p=0.67 |  |  |  |  |
| Fleming et al., 2009 [9] | **Qualitative** | **Fear of side effects:**   - Children and adults become absent from school / villages during MDAs to avoid experiencing side effects.   **Side effects indicate PZQ works:**   - Some beneficiaries thought that side effects showed that PZQ was working against SCH within the body, whist their absence indicated that individual was cured.   **Size, smell and taste of tablets:**   - Some children are afraid of size and smell of PZQ tablets.   **Biomedical knowledge of SCH:**   - Knowledge of symptoms and forms of transmission are linked to negative health and economic consequences. People are familiar with the subject due to various MDA campaigns and can associate their knowledge of the disease with the need to receive treatment.   **Awareness of being at risk:**   - People’s awareness of their exposure to (re)infection facilitated reception to treatment. - Many are aware that fishermen, women who collect water and children playing in streams and lake are exposed to disease.   **Traditional beliefs:**   - Some considered witchcraft as a source of SCH, which could not be treated with drugs.   **SCH not a major concern:**   - SCH was not considered a leading public concern compared to Malaria, HIV or stomach infections. Some community members felt this resulted from the reduction of severe cases thanks to the MDA. One group, however, still considered SCH a major health problem.   **Unclear drug-administration procedure:**   - Relying solely on height measures was considered to potentially lead to overdoses.   **Perceived health benefits:**   - Adults and children who considered feeling better or notice absence of symptoms have positive views of MDA - Most informants perceived that PZQ indeed cured SCH. | **Rumours of deaths or severe health consequences**   - Rumours that a woman died as a result of severe diarrhoea fomented fears of side effects and treatment avoidance / rejection.   **Adults’ influence:**   - Parent’s refusal to receive treatment and their fear of side effects reinforced children’s avoidance. - Many children who participated of the MDA reported that their parents encouraged them to attend school on treatment days. | **Access to health system’s support**   - In a given district, CDDs were part of village health teams and led MDA activities during 2004. Later, they played a secondary role compared to local health workers. The reason was that CDDs commitment decreased due to the lack of incentives. - In another district, CDDs were attached to Parish Development Committees, which were set up by the government alongside UNICEF. CDDs were considered a valuable resource in this context, since they enjoyed a trustful relationship with residents. They participated of MDAs as well as of other health-related campaigns (involved in home-based management of fever, malaria, sanitation and immunisation). |  | **Sensitisation: Means of sensitisation**   - MDAs were expected to provide health education through health workers, leaders, CDDS and teachers. - In certain regions, beneficiaries complained of mixed messages concerning SCH prevention (no examples provided). - There was limited evidence that SCH health education was integrated into routine health education plans for outreach services at the sub-county and village levels.   **Sensitisation: Length of time**   - Sensitisation activities often took place on date of treatment and could not reach many people.   **Incentives / compensation for distributors:**   - Distributors were mostly satisfied with their training and reported being willing to serve as distributors in subsequent years. - Distributors commonly reported lack of transport and incentives to carry out their activities. - In a study district, the lack of material incentives appears to have led to de-motivation and poor performance. Community health workers acquired a more central role progressively, whilst CDDs a supporting one.   **Budgeting issues**   - Constant issues regarding resource allocation from national as compared to district programmes were observed. In 2004, less than 50% of districts reported having supplemented the programme budget by allocating personnel and logistics. In 2005, 70.8% of district supplemented budget. - In 2004, only a third (36.7%) of the sub-county programme officials made extra budgetary provisions to the programme. - Most common complaint was the lack of funding to support activities and the expectation that the national programme would support MDAs in full.   **Organisational structure:**   - District vector control officers lacked established procedures or authority to coordinate with sub-district health managers and supervise all frontline activities (CDDs and teachers). - The managers of the health sub-district (HSD) were interviewed and they reported a minimal involvement in the programme other than storing drugs and the routine management of schistosomiasis cases. |
| Hastings et al., 2016 [10] | Qualitative | **Access to food:**   - Informing parents that children needed to eat prior MDA did not match a local a reality where children on many occasions only have one meal a day. - Requests for food in relation to side effects gave parents the impression that drugs were very strong.   **Fear of side effects:**   - Children feared dying after experiencing side effects, exacerbating parent’s rejection of MDA.   **Unclear rationale of treatment:**   - Parents questioned why MDA’s needed to target all SAC. Lack of screening pre-treatment was seen as bad practice given past experiences with other interventions (e.g., malaria). - Parents question why children need to be treated since it was likely they would be re-infected in the near future anyway.   **Unclear drug-administration procedure:**   - Parents questioned why drugs are provided based on height only. They considered there was a high-risk of overdoses.   **Perceived competence of distributors**   - Parents considered that teachers had no adequate training to administer PZQ tablets or knowledge to treat the disease**.** | **Rumours of deaths or severe health consequences**   - Rumours of fatalities after taking medicines for SCH and STH spread by radio and local networks of parents. These later turned into riots and public protests**.** - Rumours of fatalities and observation of side effects were attributed to teachers’ lack of training and health education. - In the study areas there was a public health campaign aimed at family planning, coupled with messages concerning the over-population of Africa. - In all government health care facilities there were posters encouraging the use of contraception.   **Rumours of a conspiracy by governments / foreign agencies**   - Subsequent rumours emerged that due to corruption, the programme had used expired, counterfeit or experimental drugs. - Other believed that it was a clandestine attempt sterilise new generations with support from Western countries. - Rumours could be linked to the presence of a public health campaign in the region aimed at family planning. Government health facilities showed posters encouraging the use of contraception and dissemination messages alluded to the over-population of Africa. |  |  | **Sensitisation: content**   - The health education that provided during these meetings appears to have been minimal. Teachers informed people that treatment would take place, but not necessarily the rationale for the targeted MDA or the method in which the drug dose would be calculated.   **Sensitisation: means of sensitisation**   - Normally, information passes ‘down’ from central to municipal/district to ward and communities. This approach was by-passed on this occasion, since schools were named responsible for informing the wider community. Moreover, the majority of chairpersons and other community-based organisations were not informed.   **Sensitisation: length of time**   - Teachers were given insufficient time to adequately inform parents. In most cases, meetings were held at the schools one or two days before MDAs. Even when parents were aware of these meetings, many complained they had been given insufficient notice to attend. |
| Knopp et al, 2016 [11] | **Qualitative** | **Fear of side effects:**   - Adults and children expressed concerns about adverse events, including abdominal discomfort, dizziness, and vomiting.   **Size, smell, and taste of tablets:**   - Beneficiaries had difficulties with large size of tablets and the number of tablets they need to consume. |  |  |  |  |
|  | **Quantitative**^(a)^  **^(descriptive)^** | **NOTE:** Results come from frequency tables reporting reasons for not taking drugs among non-compliers. Reasons with frequencies over 10% considered | | | | |
|  |  | **Absenteeism**:   - Absent <5 days: 18.7% - Absent >5 days: 21.6%   **Pregnancy:**   - Pregnant: 16.7% |  |  |  |  |
| Lothe et al., 2018 [12] | Qualitative | **Sex:**   - No evidence of specific trends. There were contradictory views of who participates more depending on sex of pupil. Boys considered participation was high among them since they commonly play in rivers and hence were more exposed. Girls, in turn, considered that peer pressure affected participation more among boys. Moreover, it was believed that SCH is more serious for girls due to their anatomy.   **Fear of side effects:**   - Some pupils report having heard of children from a nearby village who had headaches and stomach-ache after treatment and so were reluctant to receive treatment.   **Biomedical knowledge of SCH**   - Participants believed that SCH could be sexually transmitted (“it’s the blood that is dirty inside you”). This affects disclosure and treatment-seeking behaviour. - Some informants reported that people believed that girls tried to disguise having an STD by saying they have SCH.   **Awareness of being at risk**   - All participants knew that rivers and dams were places where one might get SCH. Parents on occasions forbade children from swimming and playing in the rivers.   **SCH not a major concern**   - Most participants considered that SCH is a disease that usually heals on its own. - Most informants did not consider SCH a chronic or severe condition. - Some do not consider SCH a major public health problem since they are not aware of deaths related to the disease.   **Traditional beliefs**   - In Zulu tradition, illnesses can be specific to African people, (“ukufa kwabantu”) or generic (“umkhuhlane”). The first need African remedies. SCH was categorized as the latter by some. Young people were less likely to believe those explanations. However, others followed the advice of parents or elderly relatives and visited healers and herbalists. | **Peer pressure:**   - Some pupils said that those who accept treatment are likely to be teased by fellow-pupils as it indicates they have the disease. - Some children reported that SCH was perceived as a rural disease, so that having SCH could produce embarrassment as children would appear unsophisticated. - SAC on occasions use the term ‘dog-pills’ to identify PZQ tablets. The term was used to make fun of treatment recipients. - Sexual association with SCH also generates a sense of embarrassment among girls who seek treatment publicly.   **Adults influence**   - Several of the adult participants (teachers and community participants) said that the transfer of knowledge between parents and their children was more common 40 years ago than currently. - Many pupils confirmed that they do not speak to either of their parents about everyday challenges - In some families, it was considered the elder’s responsibility to educate the grandchildren. However, most participants did not have grandparents, as they had passed away. | **Curricula restrictions for health education**   - Pupils highlighted that SCH had not been addressed in any of the school subjects. Teachers said it could be incorporated into the Life Orientation syllabus but felt that the curriculum is already cluttered with topics. |  |  |
| Mafe et al., 2005 [13] | Quantitative^(a),(b,^  (significant) | **NOTE:** Results come from descriptive statistics concerning reasons for not receiving treatment as well as cross-tabulations of coverage results for sub- groups according to age, sex and distribution approach (hospital facility, school-based or community-wide). No additional multivariate analysis provided in text. | | | | |
|  |  | **Age** (p<0,05)**:**   - 5-9 (HF 32.2%; SBT: 24.3%; CWT: 51.2%); - 10-14 (HF: 44.5%, SBT: 45.8%, CWT 33.8%); - 15-19 (HF: 23.3%, SBT 29.9%, CWT: 15.1%).   **Basic operational information:**   - 64.7% of untreated SAC reported it was due to ignorance of treatment activity.   **Absenteeism**:   - 20.6% of those missing treatment said it was due to their absence from home on treatment day. |  |  | **Setting’s size**^(a)^**:**  - The least coverage was obtained in settings with larger and more dispersed populations: Ilewo Orile (pop=2422, coverage= 39.5%, Imala (pop=4895, coverage=26.3%). One used central distribution and the other SBT | **Distribution strategy**^(a)^:   - CWT overall coverage 72.2% (range: 69.0%. – 73.0% - HF overall coverage: 44.3% (range: 39.5% - 62.0%) - SBT overall coverage 28.5% (range: 26.3% - 74.5%). |
|  | Quantitative (non-significant) | **Sex by age** (p>0.05):   - Male – Female (5–9): HF: 31.6% vs 32.8; SBT: 20.6 vs 28.2; CWT: 49.4 vs 53.1%) - Male – Female (10–14): HF: 41.1% vs 48.5%; SBT: 49.0% vs 41.5%; CWT: 29.2 vs 38.6% - Male – Female (15–19): HF: 27.3% vs 18.7%; SBT 29.5% vs 30.3%; CWT: 21.4% vs 8.3%) |  |  |  |  |
| Massa et al., 2009a [14] |  | **School enrolment:**   - Many non-enrolled children lived far away; it was difficult for parents to encourage them to go to school for MDAs.   **Fear of side effects:**   - Parents complained about side effects, likely to have resulted from not eating prior treatment (CWT). Teachers faced similar problems.   **Unclear rationale for treatment:**   - Some parents considered that children may not need treatment given the lack of any symptoms   **Perceived competence of distributors:**   - For CWTs, CDDs were selected by residents if they believed they would be able to distribute the drugs. Residents were familiar with selected CDDs and teachers, fomenting trust in the MDAs. |  |  | **Setting’s size:**   - In CWT, it became difficult to reach sub-villages located at long distance from main living areas. Residents were less likely to receive information or participate of sensitisation or mobilisation activities. - Many non-enrolled children did not come to schools for treatment because they lived in areas located very far from the school.   **Community support:**   - Leaders actively participated in selecting suitable drug distribution strategies for their villages. - In two villages, the community hired bicycles to support distribution. - For CWT, villagers considered that village leaders’ involvement in mobilisation and sensitisation was important.   **Leaders’ perceived health benefits**   - Leaders’ commitment strengthened after perceiving that children no longer had haematuria after treatment. | **Sensitisation: content**   - The intervention conducted village meetings that discussed SCH transmission, infection control through mass chemotherapy, presence and management of side-effects. There was, generally speaking, good awareness of activities. However, for CWT, parents requested more education on the drugs and side effects.   **Sensitisation: means of sensitisation**   - CWT and SBT approaches used their routine village meetings to inform about MDAs. In addition, there is information that villages held special meetings to further sensitise residents. This facilitated dissemination of key information.   **Incentives / compensation for distributors:**   - CDDs and teachers mentioned they would like to receive some incentives for their work. However, they did not consider it essential to their work.   **Intrinsic rewards for distributors**   - Despite the lack of incentives, some CDDs considered their duty to help their communities. Others reported that public recognition as ‘doctors’ was prestigious.   **Distribution strategy:**   - Beneficiaries expressed satisfaction with both CWT and SBT. However, residents considered SBT is less able to reach children not enrolled in school. Leaders were concerned about reaching non-enrolled SAC. |
| Massa et al., 2009b [15] | Quantitative^(a)^  (significant) | **School enrolment:**  ROUND 1   - School enrolled vs non-enrolled (CWT): 80.3% vs 80.0% - School enrolled vs. non-enrolled (SBT): 82.1% vs 59.2%   ROUND 2   - School enrolled vs non-enrolled (CWT): 81.9% vs 82.9% - School-enrolled vs non-enrolled (SBT): 83.0 vs 56.6%. |  |  |  | **Distribution strategy:**  ROUND 1   - CWT vs SBT among school enrolled SAC: 80.3% vs 82.1%, p=0.07 - CWT vs SBT among non-enrolled SAC: 80% vs SBT (59.2%), p<0.001   ROUND 2   - CWT vs SBT among school enrolled SAC: 81.9% vs 83.0, p=0.24. - CWT vs SBT among non-enrolled SAC: 82.9% vs SBT 56.6%, p<0.001 |
|  | Non-significant | **NOTE:** Results reported below are only for enrolled SAC, since the number of cases for non-enrolled children are too few for meaningful interpretation (n<12 in both rounds for age groups 9+) | | | | |
|  |  | **Age** (enrolled SAC):  a. ROUND 1   - 6 to 8: 79.6% (CWT) vs 81.5% (SBT), p=0.27 - 9 to 12: 80.5% (CWT) vs 82.2% (SBT), p=0.20 - 13 to 15: 80.7% (CWT) vs 83.1% (SBT), p=0.43   B. ROUND 2   - 6 to 8: 81.8% (CWT) vs 83.4% (SBT), p=0.24 - 9 to 12: 82.4% (CWT) vs 83.1% (SBT), p=0.65 - 13 to 15: 80.5% (CWT) vs 81.6% (SBT), p=0.87 |  |  |  | **Distribution strategy** (overall)**:**  ROUND 1   - CWT vs SBT total among SAC: 80.2% vs 80.7%, p=0.66   ROUND 2   - CWT vs SBT among SAC: 81.9% vs. 81.4%, p=0.58 |
| Muhumuza et al., 2013 [16] | Quantitative  (descriptive) | **NOTE:** Results below come from descriptive statistics concerning reasons for not receiving treatment reported by SAC who did not consume tablets. | | | | |
|  |  | **Fear of side effects:**   - 72% of those who did not took PZQ, reported fears of side effects. |  |  |  |  |
|  | **Quantitative** (significant) | **Biomedical knowledge:**   - Can name at least one valid form of transmission and one form of prevention. AOR=2.04, 95%CI (1.23-3.45), p=0.01 | **Adults’ influence**:   - Children considered teacher supported PC treatment. AOR=2.63 95%CI (1.25 – 5.55), p=0.01 |  |  |  |
|  | Quantitative  (not-significant) | **NOTE:** Variables listed in this section were eliminated in stepwise regression. No statistical tests reported | | | | |
|  |  | - **Age** (p>0.05) - **Sex** (p>0.05) - **Education** (grade) (p>0.05)   **Household’s assets / infrastructure**   - Sanitation facilities at home (p>0.05) - Water sources at home (p>0.05)   **Biomedical knowledge of SCH**   - Knowledge of SCH (p>0.05) - Lake water main source for domestic use (p>0.05)   **Awareness of being at risk**   - Think SCH is a problem in their area (p>0.05) - Think they are at risk of acquiring SCH (p>0.05)   **Preventive behaviour:**   - Think it is important to take preventive treatment (p>0.05) - Likely to take PZQ at subsequent MDAs (p>0.05) | **Adults’ influence**:   - Children considered having family support (p>0.05) - Children considered having peer support (p>0.05) | **School’s infrastructure**   - Lake is water source: school (p>0.05) - Sanitation facilities school (p>0.05)   **Access to health system’s support**   - Children considered having health worker support (p>0.05) | **Location:**   - Distance from area of residence to Lake Victoria (p>0.05) |  |
| Muhumuza et al., 2014 [17] | **Quantitative**  (significant) | **Access to food:**   - Student received mango juice and doughnuts prior treatment. AOR: 4.61, 95%CI(2.48 – 8.58), p<0.01 |  |  |  | **Sensitisation: content**   - Received sensitisation (*) about SCH prevention: AOR=13.1, 95% CI(8.29 – 20.90), p<0.001   (*) Twice a week 30m health education sessions on dangers of SCH, transmission and prevention, as well as taking PZQ to avoid getting this serious disease and importance of food consumption to avoid side effects. |
|  | **Quantitative**  (not-significant) | **NOTE:** Forward step-wise elimination process followed. Statistical results for demographic variables, fear of side effects and infection status were not reported since they were not considered for final model. | | | | |
|  |  | - **Age** (p>0.05) - **Sex** (p>0.05) - **Education** (grade) (p>0.05)   **Fear of side effects**   - Side effects attributable to PZQ (Yes / No) (p>0.05)   **Infection status**   - S. mansoni infection (Positive / Negative) (p>0.05) - Intensity (GMI epg) (p>0.05).   **Biomedical knowledge:**   - Children could mention at least one method of transmission and two methods of prevention: AOR= 1.39, 95%CI (0.68–2.94), p=0.39   **Preventive behaviour**   - Child goes to the lake: AOR=1.52, 95%CI(0.87-2.68), p=0.14 |  |  | **Location:**   - Distance from area of residence to Lake Victoria (=<5Km/>5Km): AOR=1.00, 95%CI (0.59–1.70), p=0.99 |  |
| Muhumuza et al., 2015a [18] | **Quantitative**  (significant) | **Access to food:**   - Ate food prior treatment. ARR 1.01, 95%CI: 1.00 – 1.02, p=0.02 |  |  |  |  |
|  | **Quantitative**  (not-significant) | **NOTE:** Backward elimination method used. Statistical results for age, sex, education, fear of side effects, and infection status not reported since they were not considered for final model. | | | | |
|  |  | - **Age** (p>0.05) - **Sex** (p>0.05) - Education (grade) (p>0.05)   **Fear of side effects**   - Side effects attributable to PZQ (Yes / No) (p>0.05)   **Infection status**   - S. mansoni infection (Positive / Negative) (p>0.05) - Intensity (GMI epg) (p>0.05).   **Biomedical knowledge:**   - Children could mention at least one method of transmission and two methods of prevention: ARR= 1.02, 95%CI (0.99–1.04), p=0.174. |  |  | **Location:**   - Distance from residence to Lake Victoria =<5Km ARR=1.00, 95%CI (0.99–1.00), p= 0.343 | **Sensitisation campaign: sources**   - Listened to information about MDAs via radio and spot messages (Yes/No): ARR=1.01, 95%CI (0.01–1.03), p=0.247 |
| Muhumuza et al., 2015b [19] |  | **Access to food:**   - Schools recommend food / snacks but do not provide any support. Lack of food affected willingness to participate. - Some teachers are afraid of providing treatment to children who have no food.   **Fear of side effects:**   - Some children avoided school on MDA days for fear of experiencing side effects.   **Size, smell, and taste of tablets:**   - Children expressed concern regarding the taste, smell and size of PZQ tablets.   **Biomedical knowledge of SCH:**   - Children were aware of SCH as an illness. However, it was mostly defined in general terms, as swollen stomach and an illness related to contact with water. - Children, lacked detailed knowledge on forms of transmission and prevention. Many, particularly those living far from the lake, confused it sanitation issues (e.g., can prevent boiling water or washing hands). - Lack of knowledge was found to be linked to SAC’s limited willingness to receive treatment.   **Awareness of being at risk:**   - Children from villages closer to Lake Victoria expressed greater awareness of exposure to SCH and risk of contagion.   **Unclear rationale of treatment:**   - Children were unsure why they needed treated if didn’t feel ill or showed no symptoms. |  |  |  | **Sensitisation: content**   - Informants emphasised that health education was insufficient and that the distribution of drugs takes precedence with minimal, if any, communication about the rationale of treatment. - Health education campaigns by MDAs focused on areas suspected to be endemic (islands and places close to lake). Children attending schools closer to lake were more aware of SCH and transmission / prevention than those away from the lake.   **Sensitisation: training for distributors**   - Teachers reported that they themselves are not sufficiently trained to educate children on the MDAs’ rationale. - 2 teachers are trained per school. Inadequate funding to facilitate training of more teachers was reported by the District Vector Control Officer - Lack of trained teachers and resources limit schools’ capacity for sensitisation activities.   **Material incentives / compensation for distributors**   - Teachers receive a small allowance of 2USA$ and a t-shirt. - However, teachers do not consider MDAs as part of their routine tasks but as extra work. Most considered incentives insufficient so that their contribution to the programme was not fully acknowledged nor their time properly compensated. - Filling in registers add tedious work to drug-distribution activities. - Teachers felt that the registers were an additional burden that demanded additional work. Most experienced difficulties filling out the current registers because they involve filling in many other drugs for different children at different times, as expected with the integrated Neglected Tropical Diseases (NTD) treatment registers. Some gave out drugs without completing the registers. |
| Ndyomugyenyi and Kabatereine, 2003 [20] | **Qualitative** |  |  |  | **Community support:**   - Many CDDs reported having received some kind of support from their communities (meals) for CWT. A minority (<20%) received some kind of financial incentive from their communities.   **Unexpected local demand**   - Villagers from a neighbouring village came to targeted areas to request treatment, which reduced the stock of PZQ and MBD. | **Intrinsic rewards**   - The programmed had not allocated incentives to CDDs, however, most were willing to continue supporting MDAs as part of their responsibility towards their communities. A minority received meals (30-45%) or small financial stipends (<20%) from the communities themselves. Teachers received no incentives but were willing to continue support in order to treat children. |
|  | **Quantitative (significant)** | **Age (SBT** - Treatment coverage: PZQ & MBD), p < 0.002:   - <7 years: 61.8% - 7 – 10 years: 81.5% - 10+ years: 83.3%.   **Access to operational information**   - 22.8% of SAC who did not participate of MDA had not received information of the availability of drugs and activity. |  |  |  | **Distribution strategy**   - PZQ & MDB SAC coverage CWT: 85% vs SBT: 79% (p=0.03). |
|  | **Quantitative (not-significant)** | **Age (**CWT - Treatment coverage: PZQ & MBD). p=0.2:   - <7 years: 79 % - 7 – 10 years: 85% - 10+ years: 86% |  |  |  |  |
| Odhiambo et al., 2016 [21] |  | **Access to food:**   - Some residents could not secure enough resources to get a meal prior treatment, demanding some allowances to follow MDA instructions.   **Livelihoods:**   - Fishermen and those who work outside town are not able to participate of MDA due to absenteeism.   **Fear of Side effects**:   - First-hand experiences of fatigue, dizziness, and vomiting deterred people from participate. - Witnessing friends and neighbours in that condition had similar effects. - One CHW was chased out from a compound whose residents experienced side effects.   **Size, smell and taste of tablets:**   - Some people complained that the tablets were too big to swallow.   **Biomedical knowledge of SCH:**   - Reception of MDA was facilitated by the fact that some people knew of the symptoms and health complications from SCH, so that they wanted an opportunity to get treatment.   **Access to operational information**:   - Beneficiaries knew of the activity beforehand due to sensitisation via radio and roadshows and so were receptive to the MDA. - MDA was confused with a STD campaign. Some did not consider MDA relevant as they reported being faithful to their partners.   **Unclear rationale of treatment:**   - Some said they did not need treatment since they had no symptoms - Some refused to receive treatment since they had not provided samples for testing, and so had no diagnosis.   **Perceived competence of distributors**:   - Community health workers were selected in village meetings as distributors based on local perceptions of their commitment and hard work. - CHW’s had conducted previous health campaigns and so had familiarity with residents and the area. This facilitated access to beneficiaries   **Religious beliefs:**   - Some churches reject modern medicine and opt to pray to heal.   **Perception of MDAs as a scarce benefit:**   - Beneficiaries were prompted to attend MDAs in the last year of the intervention, since drugs would no longer be distributed for free. | **Rumours of deaths or severe health consequences**   - A parallel deworming campaign resulted in a fatality, which was covered in local media. This negative publicity was associated to the MDA, generating fear.   **Rumours of a conspiracy by governments / foreign agencies**   - Some thought since drugs were offered for free, they were not genuine, had expired or that were distributed as part of a medical experiment. | **Access to health system’s support**   - CHW infrastructure was pre-existent to MDAs in urban areas. They have a well-developed supervisory structure by the MoH. Community health extension officers hence offered supervision to CHW for free, as part of their routine work. - The local health centre in the Area helped in the management of adverse side effects, which helped to reassure the community all was well | **Gender-values**   - A female CHW reported feeling insecure when working late in the evening trying to find household members that were absent in the morning.   **Terrain:**   - Large territories, with difficult terrain or areas prone to natural hazards (e.g., floods), were less likely to be reached during MDAs. - Some areas were too filthy and bushy, whilst some neighbourhoods were insecure. | **Sensitisation**: **means of sensitisation**   - Roadshows helped to spread information about MDAs and facilitated reception across the area. Residents were aware of the objectives of the intervention and of operational details. - Support for sensitisation through health facilities helped in sensitisation by informing residents who went to the hospital understand the benefits of treatment.   **Sensitisation**: **content**   - CHWs recommended that health education messages should entail not only the importance of treatment but also encourage people to construct and use latrines for waste disposal.   **Material incentives / compensation for distributors:**   - CHWs were provided with treatment booklets, bags, T-shirts, respective dose poles and the drugs for distribution. - CHWs welcomed daily lunch allowance (provided by the study) but requested additional lunch support since they had to walk the whole day and had no time to go back home to eat. - CHWs requested more than one T shirt so that they could change clothes. - Lack of material support in travelling gear (mud boots / umbrellas) limited CHWs capacity to work all day / reach distant sites. - Direct Observed treatment was recommended to be carried out to ensure proper dosage is received. However, residents preferred to take the drugs at certain times of the day, forcing CHWs to go back and forth in order to observe. - Some residents believed distributors received money according to the number of people they treated, prompting them to request incentives in exchange for their participation.   **Timing of MDA:**   - Time allocated to complete MDA is perceived as too short to reach the entire population, people demanded to extend MDA to two weeks (as compared to one). |
| Omedo et al., 2012 [22] |  | **Access to food:**   - Because the drug could not be taken on an empty stomach, the issue of hunger was also raised as a major challenge to MDA compliance - MDA took place during the planting season, so people complained. Then, residents usually work until late and only eat at night. During the mornings, they usually consume a light meal (‘tea’). Locals requested MDA should occur after harvesting to ensure food access.   **Livelihoods:**   - MDA took place in a time when many residents were tending their farms and hence were absent from town. Sometimes CDDs have to visit residents at their gardens to provide treatment. Sometimes they are unavailable or cannot eat until late, hence preferring to avoid treatment.   **Fear of side effects:**   - Residents refuse to take drugs for fear of experiencing side effects, like vomits and diarrhoea. - Some waited to see what would happen to others before taking the medicines.   **Access to operational information:**   - Some residents rejected the drugs because they had no information about the MDA. They said they had no news from the newspapers or the radio.   **Confusion with other health campaigns:**   - Some thought that the drugs were for HIV. - Others confused MDA campaigns with interventions against malaria and demanded mosquito nets prior to taking PZQ tablets.   **Unclear rationale of treatment:**   - Some did not understand why they needed to take the drugs without any prior testing.   **Perception of health benefits:**   - After noticing that those who received treatment improved in their health, demand for PZQ drugs increased. However, this on occasions happened after distribution finished. - Some beneficiaries were thankful to the programme after perceiving rates of prevalence decreased in the region.   **Perception of distributors’ competence:**   - Some beneficiaries refused treatment since they considered CDDs were not health professionals or lacked some form of certification.   **Religious beliefs:**   - Some churches reject modern medicine and opt to pray to heal. However, whilst adults rejected treatment, they allowed children to receive PZQ. | **Rumours of deaths or severe health consequences**   - Some believed that the drugs could produce cancer.   **Rumours of a conspiracy by governments / foreign agencies**   - Others considered that the MDA it was a government’s conspiracy to decimate certain ethnic communities. | **Access to health system’s support**   - CHWs had the capacity to transfer cases of severe side effects to local health facilities. - - The most common method of side effect management identified by the CHWs was referral of the participants to the nearest health facility | **Gender values**   - Almost half of the CHWs were women and had problems balancing their family duties and those of the MDA. Some endured abuse from their husbands for coming home late (e.g., evening distribution). Several indicated that they had t justify their work was profitable to avoid abuse (i.e., eventually they would be paid). | **Sensitisation:** **content**   - Sensitisation should address key issues, such as side effects, drug-administration procedure and rationale behind treating all, even those asymptomatic.   **Incentives / compensation for distributors:**   - CWT: distributors spent much time away from home, sometime in the evenings**.** In some instances, the CHWs followed the persons wherever they were, for example in their farming plots. - Distributors were volunteers but many had the expectation of material incentives, such as allowances, gum boots, umbrellas or bicycles (to protect against elements whilst visiting homes). They were influenced by the fact that other health campaigns frequently involve such rewards.   **Intrinsic rewards for distributors:**   - However, Immaterial benefits could be a powerful driver to promote distributors’ engagement, such as community recognition, satisfaction of making a contribution to the community, pride in providing a service, knowledge gained, and positive feedback from individual community members - Many were driven they the desire to serve their communities, including their own children.   **Staff coordination:**   - Enumerators in charge of registering beneficiaries sabotaged distributors since they consider they would be the ones distributing the PZQ tablets. This delayed or increased distributors’ workload. Some enumerators did not surrender registers and CDDs had to census beneficiaries whilst implementing the MDA. |
| Omedo et al., 2014 [23] |  | **Fear of side effects**   - People reported cases of side effects. Despite some concern, sensitisation campaign reduced anxiety so that fear (and avoidance) was reduced.   **Biomedical knowledge of SCH**   - Sensitisation campaign improved knowledge of SCH in terms of sources of transmission and preventive measures. - Increased awareness of the disease was found to facilitate reception of treatment by CHWs.   **Access to operational information**   - Residents were well informed of the campaign. They were expecting the CHWs and knew of the intended uses of the drugs. |  |  | **Community support**   - Local authorities (e.g., provincial administrators, church leaders, BMU leaders, women’s groups, etc) participated of sensitisation and mobilisation. This provided social recognition to CHWs’ work and enhanced local trust. - Broad engagement of authorities brought further opportunities for dissemination (e.g., through preachers). They also provided security to the CHWs during the exercise. | **Sensitisation: means of sensitisation**   - Multipronged community sensitisation and mobilisation campaign improved uptake by reducing anxiety of side effect and increasing awareness of MDA efforts, generating demand among residents. This was achieved through radio messages, leaflets, roadshows, personal communication with leaders, and village meetings. - Radio messages helped to reach a wider audience than targeted populations. Response from listeners facilitated dialogue around side effects and associated rumours’   **Sensitisation: content**   - In addition to providing information about SCH and operational details of the MDA campaign, CHWs found that people were aware of the side effects and reduced anxiety among beneficiaries. |
| Parker et al., 2008 [24] |  | **Livelihoods**   - The worst off were particularly concerned about the risk of side effects as they could compromise their capacity to work and so to support their precarious living conditions.   **Fear of side effects:**   - Not all informants had experienced side-effects, but all had experiences within their immediate or extended families. Concerns about the severity of side effects generated fear and antipathy towards the MDA. - Perceptions of the strength of the drug prompted reticence among those who are ill or have a long-term condition (e.g., HIV), as well as among the poorest, given the potential impact on livelihoods. - Pregnant women fear miscarrying.   **Biomedical knowledge of SCH:**   - Local understandings of SCH are ambiguous. Residents generally identify it with ‘worms”, but as such they associate SCH with sanitation and drinking water. Some link representations of PZQ parasites with earth worms, finding difficult to understand how they get into people’s bodies. - Likewise, symptoms are generally understood in terms of stomach and food issues – diarrhoea and swollen stomach. Connection with a disease is not clear, some refer to a general condition linked to various issues such as hunger or sanitation.   **Traditional beliefs:**   - Symptoms of SCH are on occasions associated with ‘awola’, an illness that is inflicted on an individual by a witch due to envy. Treatment is provided by witchdoctors and imply various rites. - There is the belief that modern medicines can be dangerous if consumed whilst enduring ‘awola’. - On occasions, however, people may consult herbalists at the same time as getting modern treatment.   **Unclear rationale for treatment**   - Some questioned why treatment was needed if they felt healthy.   **Unclear drug-administration procedure:**   - Local politicians and other villagers, in particular, expressed a concern that incorrect quantities of drugs were being administered as a consequence of allocating drugs according to height, rather than weight or age. | **Rumours of deaths or severe health consequences**   - Rumours that drugs are very strong & potentially dangerous. Risk of death due to overdose (stories of children dying). - Some married men expressed were concerned with the idea that PZQ produces infertility.   **Rumours of a conspiracy by governments / foreign agencies**   - There was some discussion as to whether the Ugandan government was colluding with the USA to reduce the birth rate in the country. | **Programme officials’ attitudes**   - Distribution typically relies on a small group of staff who are based in the district capital or the country’s capital. Officials leading the intervention may not be familiar to villagers’ lifestyles or customs whilst others, who know them, tend to disparage of them. Some may not speak local dialect, may show some kind of prejudice (labelling residents as ‘drunkards, promiscuous, stubborn, etc.) or are unwilling to spend time and socialise locally (e.g. not leaving cars or not eating local food). | **Community cohesion:**   - A study area had a heterogenous population and there’s be a history of conflict between long-term residents and more recent migrants, including foreigners (from Congo). There is substantive distrust between neighbours then. | **Sensitisation: training for distributors**   - Most distributors and health workers were familiar with schistosomiasis but had no information on the rationale of MDAs (regular treatment without a diagnosis) or the role of regular MDAs as a preventive mechanism for epidemic conditions and health complications.   **Sensitisation: content**   - Informative events and materials use a biomedical perspective that are not easy appropriation by locals (e.g., views on worms and disease). - Most printed material are not in a local language   Materials discuss SCH but do not address MDAs’ rationale  **Incentives / compensation for distributors:**   - Community-based intervention relies on volunteers. - Lack of material support or compensation generate annoyance and limited disposition to do the work, particularly since the second year. CDDs persistently expressed irritation at how much they were being asked to do without remuneration, which involve significant opportunity costs. In some cases, they retained registers until receiving some form of compensation. - Several informants, including health staff, were open about the fact that taking on responsibility for distributing drugs was a calculation based on how much might be given in the form of food and other commodities (such as bicycles), how much might be earned by selling drugs in the open market, and/or whether or not it was worth accumulating a personal store of the drugs in case a member of their own family should fall ill in the future.   **Intrinsic rewards:**   - In some settings, local council members and others have been willing to distribute drugs, in part because it lends them status and because they see themselves as assisting their neighbours and relatives, in other settings volunteer work generated problems. |
| Parker & Allen, 2011 [25] |  | **Access to food:**   - Few people are willing to swallow the tablets if they feel they have insufficient food to mitigate the side effects. - In a study area, there was secured access to food due to a WFP programme that provided regular access to food. Once the programme stopped, however, uptake dropped. Teachers were concerned of distributing drugs among children with empty stomachs.   **Livelihoods**   - Kin relations between residents in Uganda and Congo are strong, with substantial trading networks. People are constantly travelling to buy and sell fish, grain and other produce.   **Fear of side effects:**   - Many informants expressed fear of treatment due to side effects, including diarrhoea, stomach pain and vomit. - Reasons for presence of side effects remain unclear to residents.   **Side effects indicate PZQ works:**   - Residents noticed a decline in the number of cases of side effects. Some interpreted this as an indication that they are not working any more.   **Traditional beliefs:**   - Over time, 3 years later, residents no longer linked ‘awola’ to SCH, so that this did not affect uptake   **Unclear MDA rationale**   - Residents are still unclear about why drugs are needed despite absence of symptoms.   **Unclear procedure**   - It remains unclear to residents why height is the main indicators to estimate dosage.   **Perceptions of health benefits**   - Despite the presence of side effects, residents could observe that people improved their health over time. | **Rumours of deaths or severe health consequences**   - Presence of side effects lead to rumours of severe consequences from treatment, including fatalities. |  | **Community engagement:**   - The Catholic Church was very influential in the Mojo and Adjumani districts (2 out of 4 study areas). It actively supports public health interventions, such as immunization programmes, child health days, and endeavours to control NTDs.   **Gender-related issues**   - Female drug distributors revealed that it was impossible for a woman to insist that older men took PZQ tablets under her gaze.   **Past public health interventions**   - Past public health campaigns aimed at disease control (e.g., sleeping sickness) took place in areas receptive to MDAs.   **Migration patterns:**   - Community is located near to the Congo frontier, that implies that hundreds of people are constantly on the move due to trading and fishing activities. It is hence extremely difficult to plan ahead for treatment activities to account for stable and mobile residents. Many non-residents end up being treated. This limits the availability of drugs for regular residents.   **Political factors:**   - Security concerns affect local economy in frontier areas (Sudan conflict and government’s fight w/ Lords’ Resistance Army) generated mistrust. Associated violence resulted in displacement of populations. - Some internal displacement camps, however, “benefited” from having concentrated populations in small areas that, in addition, were used to the presence of international organisations. International assistance is thus well valued among such groups. Moreover, food assistance from WFP ensured locals had regular access to food. This facilitated the MDA request of swallowing PZQ tablets after eating. After refugees returned and food assistance stopped, there was renewed resistance to MDAs. | **Sensitisation: content**   - Informative events and materials use a biomedical perspective that are not easy appropriation by locals (e.g., views on worms and disease). - School textbooks only speak of S. haematobium, not other species. - Materials discuss SCH but do not address MDAs’ rationale   **Sensitisation: Training for distributors**   - Teachers and distributors are expected to understand the MDA’s rationale and the cycle of transmission of SCH but that’s rarely the case. Proper training about the aetiology of NTDs and rationale for MDAs is restricted to district-level staff. - Most commonly, training provision for distributors centre on the distribution process. - The rationale of the provision of MDA has not been adequately communicated to drug distributors. They were unable to answer residents’ queries.   **Incentives / compensation for distributors**   - The majority of drug distributors said they felt too poor to work without remuneration due to their domestic / economic obligations against the amount of labour involved. (“There is no incentive to work .. there are no uniforms, no bicycles for transport.”)   **Intrinsic rewards for distributors**   - These competing needs, in turn, are considered to affect their performance. During Year 1, community volunteers supported MDAs in part because of the social recognition they obtain from their activities. However, next year many consider the labour demands too high. |
| Pearson, 2016 [26] |  | **Livelihoods**   - Many were occupied fishing, cultivating, taking produce to markets, or visiting relatives.   **Fear of side effects**   - Some were reluctant due to concerns over side effects.   **Side effects indicate PZQ works**   - Some, however, considered their presence indicated that the medicine was working (e.g., ‘it means you have worms’).   **Awareness of being at risk:**   - **Al**though most were not fully aware of transmission cycle, they were aware of being at risk, since the disease was related to water contact. - Effect is conditioned on perception of seriousness (see below).   **SCH as a major health concern**   - Some questioned the need to take drugs since their parents and grandparents lived to an old age despite constant contact with river water.   **Traditional beliefs**:   - Local use of medicinal herbs can affect participation but is also compatible with modern medicine. People see them as second-best, use them if no drugs are not accessible due to cost.   **Unclear rationale of treatment:**   - Residents were reluctant to take pills since they felt healthy, showed no symptoms, and had not been tested for SCH.   **Perceived health benefits:**   - There was a general perception that the MDAs reduced the rate of the disease   **Alcohol consumption:**   - Some missed treatment since they were drinking on day prior to MDA and were unable to participate. |  |  | **Past public health interventions**   - Local acceptance may be linked to past experiences of health programmes (e.g. sleep sickness), in addition to recent humanitarian and relief campaigns, with support of the local Catholic Church.   **Migration patterns:**   - Villages’ location next to international borders result in constant travelling and complex seasonal migration patterns, affecting the programme’s capacity to reach and treat all ‘residents. It becomes extremely difficult to estimate the size of the target population. |  |
| Randjelovic et al., 2015 [27] |  | **Age:**   - Reference: 3-9 years of age - 10–14: AOR=0.70, 95%CI (0.63–0.78), p<0.001 - 15–19: AOR=0.39, 95%CI (0.35–0.43), p<0.001 - 20–24: AOR=0.28, 95%CI (0.23–0.33), p<0.001 - 25–35: AOR=0.10, 95%CI (0.03–0.35), p<0.001   **Sex (**Reference: Female)   - Male: AOR=0.91, 95%CI (0.85–0.99), p=0.02 |  | **(*) Size belongs to organisational considerations but for consistency, it is assigned to community level factors. -🡪** | **Setting’s size** (Reference: School <350 learners)   - 350-700 learners**:** AOR=0.48, 95%CI (0.40–0.58), p< 0.001 - 700 learners: AOR=0.47 AOR, 95%CI (0.39–0.56), p<0.001 |  |
| Rilkoff et al., 2015 [28] |  | **Sex:**   - Women in general were found to be more accepting of the programme and more willing to accept the advice provided by CMDs.   **Access to food:**   - Many women had not eaten before the treatment was brought and so was were reluctant to take the treatment on an empty stomach for fear of side effects.   **Livelihoods:**   - Due to their domestic roles, women were more likely to be adequately informed of MDA activities and present at home during CDDs’ visits. - Economic activities such as farming, trading or truck driving resulted in absenteeism during distribution hours. Men were the most affected.   **Unclear rationale of treatment:**   - Few residents were adequately informed of the intended purpose of MDAs. - Men were more likely to challenge the need for treatment since they felt healthy and showed no symptoms.   **Perceived competence of distributors:**   - Community leaders and men considered that CDDs were no informed enough about the medicines and the programme and requested that “experts” be brought to their villages to explain the reasons to take PZQ as well as SCH’s symptoms, consequences and transmission. - In a specific community, women were reluctant to accept treatment since they didn’t trust their village health team due to a fatality in a past vaccination campaign.   **Pregnancy:**   - Since different criteria applied according to drug in question, it was confusing for pregnant women to understand when and why should they be given treatment. Most were unaware of the type of treatment they should receive. | **Rumours of deaths or severe health consequences**   - Male networks spread more rumours about harmful effects or the lack of need of treatment in 2 villages. However, in one village it was women who rejected treatment due to stories of deaths following a past vaccination campaign.   **Peer pressure**:   - Boys were considered to be more likely to be influenced by their peers.   **Adults’ influence:**   - Boys were perceived to be more likely to be negatively influenced by adults males, whether their relatives or not with whom they shared leisure activities (e.g., fishing). - Girls were perceived to be more likely to accept treatment if their parents supported the MDA as well as the advice provided by CMDs. |  |  | **Sensitisation: Training for distributors**   - Very few CDDs reported having received training on causes and transmission of NTDs. - CDDs considered not being able to absolve questions from beneficiaries, particularly pregnant women and men, negative messages on side effects or the rationale behind MDAs. - In 7 of 8 communities studied, CMDs expressed confusion or presented information that was contrary to that provided by the programme.   **Material incentives / compensation for distributors**   - House-house distribution placed significant burden to CDDs. Sometimes they needed to walk significant time to visit each HH and then revisit them to locate those absent during first visit. This was further problematic when people were absent, since CMDs had to revisit those places. |
| Sanya et al., 2017 [29] |  | **Livelihoods:**   - Residents who work reported being too busy with work. Fishermen were less likely to receive treatment. - Men (fishers) were considered more likely to dodge treatment.   **Fear of side effects:**   - Beneficiaries were concerned about the presence of dizziness, vomiting, fatigue and diarrhoea and so avoided treatment.   **Side effects indicate PZQ works:**   - However, most considered that side-effects showed that treatment was effective, a consequence of getting rid of SCH.   **Biomedical knowledge of SCH**   - Despite having heard about SCH, there is confusion about the sources of infection. Most correctly stated that contact with infested lake water and open defecation were sources of infection but some alluded to eating half-cooked food or food that has been contaminated by flies.   **Awareness of being at risk:**   - All informants considered that those who live and work in study villages were at risk; particularly fishermen, women doing the laundry and children who play in the lake. - Effect on participation appears conditioned on perception of seriousness (see below).   **SCH not a major public health concern:**   - Many participants did not consider SCH a major health problem. It used to be in the past, but no more thanks to MDAs. It is considered to be controllable   .**Perceived health benefits**   - 90/94 participants considered that MDAs of PZQ were beneficial. |  |  |  |  |
| Tuhebwe et al., 2015 [30] | Qualitative results | **Size, smell and taste of tablets**   - Some informants rejected the drugs since they are big and too many need to be swallowed. |  |  |  | **Sensitisation: means of sensitisation**   - Distributors lacked necessary materials for sensitisation, such as information education and communication materials on SCH control. - Sometimes the use of mass media (i.e., radios and films) was not suitable to island populations.   **Sensitisation: training for ditributors**   - Some distributors received no training. - Of the 15 villages studied, only 8 had distributors who received training and only 4 received support supervision.   **Material incentives / compensation for distributors**   - Lack of material support to CDDs results in high attrition among them and lack of incentives to motivate them   **Budgeting issues:**   - The programme has been successful in attracting additional partners to provide funding and involving district level agencies into planning for drug procurement and operational costs. They perceived there’s been an increase in treatment uptake as a result. - However, drugs are not always readily available partly because of limited government’s funding. The contribution from the Uganda government is perceived as low.   **Logistics:**   - Uneven distribution across sites. Some receive drugs late or not enough (run out). The islands are not easy to reach due to their location. - Lack of dose poles result in long queues, so that beneficiaries get tired or bored of waiting. |
|  | Quantitative results: significant | **Livelihoods** (reference: Non-fishing)   - Fishers: AOR=1.73, 95%CI (1.13-2.63), p=0.01   **PZQ tablet characteristics** (reference: disagree)**:**   - Drugs taste very bad (Agree): AOR=8.17, 95%CI (5.18 – 12.87), p<0.01   **Biomedical knowledge of SCH**   - Received health education (Yes/No): AOR=5.95, 95%CI (3.67-9.65), p=0.001. - Score of 4-6 over 6 regarding knowledge of drug treatment, sanitation and control of SCH, and protective behaviour (4-6 / =<3), AOR: 1.85, 95%Ci: 1.22-2.81, p=0.04.   **Access to operational information**   - Heard of MDAs (Yes / No); AOR: 1.85 (95% CI: 1.22-2.81) - Saw a poster on MDAs (Yes / No): AOR 2.00 (95% CI: 1.28–3.14)   **Perceived health benefits** (reference: Disagree)   - Treatment improves one’s health (Agree): AOR=13.96, 95%CI (1.62 – 120.41), p=0.017 |  |  |  | **Sensitisation: means of sensitisation**   - Ever saw a poster (Yes/No): AOR= 2.00, 95%CI(1.28–3.14),p=0.002 |
|  | Quantitative  (not-significant) | **NOTE:** Variables listed in this section were eliminated through a step-wise regression process. Specific statistics were not reported since they were not included in final model. | | | | |
|  |  | **Age** (p>0.05)  **Sex**, p>0.05  **Marital status** (p>0.05)  **Education** (reference: None)   - Primary / Secondary / Tertiary (p>0.05)   **Household’s assets / infrastructure**   - Sanitation facility (No / Yes) (p>0.05)   **Side effects:**   - SCH drugs can cause side effects (Agree / disagree) (p>0.05) - Treatment by MDA can cause death or bad effects / (Agree / disagree) (p>0.05)   **Awareness of risk**   - - High / Low (p>0.05)   **SCH as a major health concern**   - SCH can cause death (Agree / disagree) (p>0.05)   **Perceived need of MDA:**   - everybody should swallow PZQ (Agree / disagree) (p>0.05)   **Perceived benefits**   - MDA is effective in controlling SCH (Agree / disagree) (p>0.05) - MDA improves one’s ability to work more in the long-term (Agree / disagree) (p>0.05) | **Length of residence in village** (Ref: 1-2 years)   - 3-4 years or 5+ years (p>0.05) |  |  |  |
| **(a) No 95% CI information provided**  **(b) No statistical test details provided**  **(c) No multivariate analysis provided** | | | | | | |

**References**

1. Adeneye AK, Akinwale OP, Idowu ET, Adewale B, Manafa OU, Sulyman MA, et al. Sociocultural aspects of mass delivery of praziquantel in schistosomiasis control: The Abeokuta experience. Res Soc Adm Pharm. 2007 Jun 1;3(2):183–98.

2. Adriko M, Faust CL, Carruthers LV, Moses A, Tukahebwa EM, Lamberton PHL. Low Praziquantel Treatment Coverage for Schistosoma mansoni in Mayuge District, Uganda, Due to the Absence of Treatment Opportunities, Rather Than Systematic Non-Compliance. Trop Med Infect Dis. 2018 Oct 8;3(4).

3. Bogus J, Gankpala L, Fischer K, Krentel A, Weil GJ, Fischer PU, et al. Community Attitudes toward Mass Drug Administration for Control and Elimination of Neglected Tropical Diseases after the 2014 Outbreak of Ebola Virus Disease in Lofa County, Liberia. Am J Trop Med Hyg. 2016 Mar 2;94(3):497–503.

4. Bukindu F, Morona D, Mazigo HD. Prevalence of Schistosoma mansoni and soil transmitted helminths and factors associated with uptake of preventive chemotherapy among school children in Sengerema District in north-western Tanzania. Tanzan J Health Res [Internet]. 2016 Jan 1 [cited 2018 Feb 14];18(1). Available from: https://www.ajol.info/index.php/thrb/article/view/122544

5. Chami GF, Kontoleon AA, Bulte E, Fenwick A, Kabatereine NB, Tukahebwa EM, et al. Profiling Nonrecipients of Mass Drug Administration for Schistosomiasis and Hookworm Infections: A Comprehensive Analysis of Praziquantel and Albendazole Coverage in Community-Directed Treatment in Uganda. Clin Infect Dis. 2016 Jan 15;62(2):200–7.

6. Chami GF, Kontoleon AA, Bulte E, Fenwick A, Kabatereine NB, Tukahebwa EM, et al. Community-directed mass drug administration is undermined by status seeking in friendship networks and inadequate trust in health advice networks. Soc Sci Med. 2017 Jun 1;183:37–47.

7. Coulibaly JT, Ouattara M, Barda B, Utzinger J, N’Goran EK, Keiser J. A Rapid Appraisal of Factors Influencing Praziquantel Treatment Compliance in Two Communities Endemic for Schistosomiasis in Côte d’Ivoire. Trop Med Infect Dis. 2018 Jun 19;3(2).

8. Dabo A, Bary B, Kouriba B, Sankare O, Doumbo O. Factors associated with coverage of praziquantel for schistosomiasis control in the community-direct intervention (CDI) approach in Mali (West Africa). Infect Dis Poverty. 2013;2:11.

9. Fleming FM, Fenwick A, Tukahebwa EM, Lubanga RGN, Namwangye H, Zaramba S, et al. Process evaluation of schistosomiasis control in Uganda, 2003 to 2006: perceptions, attitudes and constraints of a national programme. Parasitology. 2009 Nov;136(13):1759–69.

10. Hastings J. Rumours, Riots and the Rejection of Mass Drug Administration for the Treatment of Schisotosomiasis in Morogoro, Tanzania. J Biosoc Sci. 2016 Sep;48(S1):S16–39.

11. Knopp S, Person B, Ame SM, Ali SM, Muhsin J, Juma S, et al. Praziquantel coverage in schools and communities targeted for the elimination of urogenital schistosomiasis in Zanzibar: a cross-sectional survey. Parasit Vectors. 2016 Jan 4;9(1):5.

12. Lothe A, Zulu N, Øyhus AO, Kjetland EF, Taylor M. Treating schistosomiasis among South African high school pupils in an endemic area, a qualitative study. BMC Infect Dis [Internet]. 2018 May 25 [cited 2019 Feb 13];18. Available from: https://www.ncbi.nlm.nih.gov/pmc/articles/PMC5970489/

13. Mafe MA, Appelt B, Adewale B, Idowu ET, Akinwale OP, Adeneye AK, et al. Effectiveness of different approaches to mass delivery of praziquantel among school-aged children in rural communities in Nigeria. Acta Trop. 2005 Feb;93(2):181–90.

14. Massa K, Magnussen P, Sheshe A, Ntakamulenga R, Ndawi B, Olsen A. Community Perceptions on the Community-Directed Treatment and School-Based Approaches for the Control of Schistomiasis and Soil-Transmitted Helminthiasis Amoong School-Age Children in Lushoto District, Tanzania. J Biosoc Sci. 2009 Jan;41(01):89.

15. Massa K, Olsen A, Sheshe A, Ntakamulenga R, Ndawi B, Magnussen P. Can coverage of schistosomiasis and soil transmitted helminthiasis control programmes targeting school-aged children be improved? New approaches. Parasitology. 2009 Nov;136(13):1781–8.

16. Muhumuza S, Olsen A, Katahoire A, Nuwaha F. Uptake of Preventive Treatment for Intestinal Schistosomiasis among School Children in Jinja District, Uganda: A Cross Sectional Study. PLOS ONE. 2013 May 7;8(5):e63438.

17. Muhumuza S, Olsen A, Katahoire A, Kiragga AN, Nuwaha F. Effectiveness of a Pre-treatment Snack on the Uptake of Mass Treatment for Schistosomiasis in Uganda: A Cluster Randomized Trial. PLOS Med. 2014 May 13;11(5):e1001640.

18. Muhumuza S, Olsen A, Katahoire A, Nuwaha F. Reduced uptake of mass treatment for schistosomiasis control in absence of food: beyond a randomized trial. BMC Infect Dis. 2015;15(1):423.

19. Muhumuza S, Olsen A, Nuwaha F, Katahoire A. Understanding Low Uptake of Mass Treatment for Intestinal Schistosmiasis Among School Children: A Qualitative Study in Jinja District, Uganda. J Biosoc Sci. 2015 Jul;47(4):505–20.

20. Ndyomugyenyi R, Kabatereine N. Integrated community-directed treatment for the control of onchocerciasis, schistosomiasis and intestinal helminths infections in Uganda: advantages and disadvantages. Trop Med Int Health. 2003;8(11):997–1004.

21. Odhiambo GO, Musuva RM, Odiere MR, Mwinzi PN. Experiences and perspectives of community health workers from implementing treatment for schistosomiasis using the community directed intervention strategy in an informal settlement in Kisumu City, western Kenya. BMC Public Health [Internet]. 2016 Sep 15 [cited 2018 Feb 18];16. Available from: https://www.ncbi.nlm.nih.gov/pmc/articles/PMC5025566/

22. Omedo MO, Matey EJ, Awiti A, Ogutu M, Alaii J, Karanja DMS, et al. Community Health Workers’ Experiences and Perspectives on Mass Drug Administration for Schistosomiasis Control in Western Kenya: The SCORE Project. Am J Trop Med Hyg. 2012 Dec 5;87(6):1065–72.

23. Omedo M, Ogutu M, Awiti A, Musuva R, Muchiri G, Montgomery SP, et al. The Effect of a Health Communication Campaign on Compliance with Mass Drug Administration for Schistosomiasis Control in Western Kenya?The SCORE Project. Am J Trop Med Hyg. 2014 Nov 5;91(5):982–8.

24. Parker M, Allen T, Hastings J. Resisting Control of Neglected Tropical Diseases: Dilemmas in the Mass Treatment of Schistosomiasis and Soil-Transmitted Helminghts in North-West Uganda. J Biosoc Sci. 2008 Mar;40(2):161–81.

25. Parker M, Allen T. Does mass drug administration for the integrated treatment of neglected tropical diseases really work? Assessing evidence for the control of schistosomiasis and soil-transmitted helminths in Uganda. Health Res Policy Syst. 2011 Jan 6;9:3.

26. Pearson G. Low Prevalence of Intestinal Schistosomiasis Among Fisherfolk Living Along the River Nile in North-Western Uganda: A Biosocial Investigation. J Biosoc Sci. 2016 Sep;48(S1):S74–91.

27. Randjelovic A, Frønæs SG, Munsami M, Kvalsvig JD, Zulu SG, Gagai S, et al. A study of hurdles in mass treatment of schistosomiasis in KwaZulu-Natal, South Africa. South Afr Fam Pract. 2015 Mar 4;57(2):57–61.

28. Rilkoff H, Tukahebwa EM, Fleming FM, Leslie J, Cole DC. Exploring Gender Dimensions of Treatment Programmes for Neglected Tropical Diseases in Uganda. PLoS Negl Trop Dis. 2013 Jul;7(7):e2312.

29. Sanya RE, Tumwesige E, Elliott AM, Seeley J. Perceptions about interventions to control schistosomiasis among the Lake Victoria island communities of Koome, Uganda. PLoS Negl Trop Dis. 2017 Oct 2;11(10):e0005982.

30. Tuhebwe D, Bagonza J, Kiracho EE, Yeka A, Elliott AM, Nuwaha F. Uptake of Mass Drug Administration Programme for Schistosomiasis Control in Koome Islands, Central Uganda. PLOS ONE. 2015 Apr 1;10(4):e0123673.
