# Supplementary material for: Factors affecting the uptake of preventive chemotherapy treatment for schistosomiasis in Sub-Saharan Africa: A systematic review
Source: PLoS Negl Trop Dis. 2021 Jan 19;15(1):e0009017. doi: 10.1371/journal.pntd.0009017 (PMC7846123; doi:10.1371/journal.pntd.0009017)
Supplement: S3 Table — (DOCX) [file pntd.0009017.s004.docx]

**S3 Table. Complete list of studies’ contributions to thematic synthesis according to target populations and types of MDA**(Categories listed only if mentioned at least twice in the literature)

| **Themes** | **Categories** | Target Populations | | Type of MDA | | |
| --- | --- | --- | --- | --- | --- | --- |
|  |  | SAC  (n=13) | SAC & Adults  (n=17) | SBT  (n=8) | CWT  (n=13) | MIXED (n=9) |
| INDIVIDUAL LEVEL | | | | | | |
| **Demographic Characteristics** | Age | Mafe et al., 2005 [1]  Ndyomugyenyi and Kabatereine, 2003 [2]  Randjelovic et al., 2015 [3] | Adriko et al., 2018 [4]  Dabo et al., 2013 [5]  Chami et al., 2017 [6] | Randjelovic et al., 2015 [3] | Dabo et al., 2013 [5]  Chami et al., 2017 [6] | Adriko et al., 2018 [4]  Mafe et al., 2005 [1] Ndyomugyenyi and Kabatereine, 2003 [2] |
|  | Sex | Randjelovic et al. , 2015 [3] | Rilkoff et al., 2013 [7] | Randjelovic et al., 2015 [3] | Rilkoff et al., 2013 [7] |  |
| **Material wellbeing** | Access to food | Bukindu et al., 2016 [8]^○^  Hastings, 2016 [9]  Muhumuza et al., 2014 [10]^○^  Muhumuza et al., 2015a [11]^○^  Muhumuza et al., 2015b [12] | Chami et al., 2017 [6]  Odhiambo et al., 2016 [13]  Omedo et al., 2012 [14]  Parker and Allen, 2011 [15] Rilkoff et al., 2013 [7] | Bukindu et al., 2016 [8]^○^  Hastings, 2016 [9]  Muhumuza et al., 2014 [10]^○^  Muhumuza et al., 2015a [11]^○^  Muhumuza et al. 2015b [12] | Chami et al., 2017 [6]  Omedo et al., 2012 [14]  Parker and Allen, 2011 [15]  Rilkoff et al., 2013 [7] | Odhiambo et al., 2016 [13] |
|  | Livelihoods |  | Chami et al., 2017 [6] Coulibaly et al 2018 [16]  Dabo et al. 2013 [5]^△^  Odhiambo et al., 2016 [13]  Omedo et al., 2012 [14]  Parker and Allen, 2011 [15]  Pearson, 2016 [17] Rilkoff et al., 2013 [7]^🞎^  Sanya et al., 2017 [18]^🞎^ Tuhebwe et al., 2015 [19] |  | Chami et al., 2017 [6]  Coulibaly et al 2018[16]  Dabo et al. 2013 [5]^△^  Omedo et al., 2012 [14]  Parker and Allen, 2011 [15]  Pearson, 2016 [17]  Rilkoff et al., 2013 [7]^🞎^ Sanya et al. 2017 [18]^🞎^ Tuhebwe et al. 2015 [19] | Odhiambo et al., 2016 [13] |
|  | School enrolment | Massa et al., 2009a [20]  Massa et al., 2009b [21] | Adriko et al., 2018 [4] |  |  | Adriko et al., 2018 [4]  Massa et al., 2009a [20]  Massa et al., 2009b [21] |
| **Drug-related factors** | Fear of side effects | Adeneye et al., 2007 [22]^○^  Hastings, 2016 [9]  Lothe et al., 2018 [23]  Massa et al., 2009a [20]  Muhumuza et al., 2013 [24]  Muhumuza et al., 2015b [12] | Chami et al., 2017 [6]  Coulibaly et al 2018 [16]  Dabo et al, 2013 [5]  Fleming et al., 2009 [25]  Knopp et al., 2016 [26]  Odhiambo et al., 2016 [13]  Omedo et al., 2012 [14]  Omedo et al., 2014 [27]^○^  Parker et al., 2008 [28]  Parker and Allen, 2011 [15]  Pearson, 2016 [17]  Sanya et al., 2017 [18] | Hastings, 2016 [9]  Lothe et al., 2018 [23]  Muhumuza et al., 2013 [24]  Muhumuza et al., 2015b [12] | Chami et al., 2017 [6]  Coulibaly et al 2018 [16]  Dabo et al, 2013 [5]  Omedo et al., 2012 [14]  Omedo et al., 2014 [27]^○^  Parker et al., 2008 [28]  Parker and Allen, 2011 [15]  Pearson, 2016 [17]  Sanya et al., 2017 [18] | Adeneye et al., 2007 [22]^○^  Fleming et al., 2009 [25]  Knopp et al., 2016 [26]  Massa et al., 2009a [20]  Odhiambo et al., 2016 [13] |
|  | Side effects indicate PZQ works | Adeneye et al., 2007 [22] | Fleming et al., 2009 [25]  Parker and Allen, 2011 [15]  Pearson, 2016 [17]  Sanya et al., 2017 [18] |  | Parker and Allen, 2011 [15]  Pearson, 2016 [17]  Sanya et al., 2017 [18] | Adeneye et al., 2007 [22]  Fleming et al., 2009 [25] |
|  | Size, smell and taste of tablets | Muhumuza et al., 2015b [12] | Coulibaly et al 2018 [16]  Fleming et al., 2009 [25]  Knopp et al., 2016 [26]  Odhiambo et al., 2016 [13]  Tuhebwe et al., 2015 [19] | Muhumuza et al., 2015b [12] | Coulibaly et al., 2018 [16]  Tuhebwe et al., 2015 [19] | Fleming et al., 2009 [25]  Knopp et al., 2016 [26]  Odhiambo et al., 2016 [13] |

| **Themes** | **Categories** | Target Populations | | Type of MDA | | |
| --- | --- | --- | --- | --- | --- | --- |
|  |  | SAC  (n=13) | SAC & Adults  (n=17) | SBT  (n=8) | CWT  (n=13) | MIXED (n=9) |
| INDIVIDUAL LEVEL | | | | | | |
| **Knowledge of SCH** | Biomedical knowledge of SCH | Adeneye et al., 2007 [22]  Lothe et al., 2018 [23]  Muhumuza et al., 2013 [24]  Muhumuza et al., 2015b [12] | Coulibaly et al., 2018 [16]  Fleming et al., 2009 [25]  Odhiambo et al., 2016 [13]  Omedo et al, 2014 [27]^○^ Parker et al., 2008 [28]  Sanya et al., 2017 [18]  Tuhebwe et al., 2015 [19] | Lothe et al., 2018 [23]  Muhumuza et al., 2013 [24]  Muhumuza et al., 2015b [12] | Coulibaly et al 2018 [16]  Omedo et al, 2014 [27]^○^  Parker et al., 2008 [28]  Sanya et al., 2017 [18] Tuhebwe et al., 2015 [19] | Adeneye et al., 2007 [22]  Fleming et al., 2009 [25]  Odhiambo et al., 2016 [13] |
|  | Awareness of being at risk | Adeneye et al., 2007 [22] Lothe et al., 2018 [23]  Muhumuza et al., 2015b [12] | Fleming et al., 2009 [25]  Sanya et al., 2017 [18] | Lothe et al., 2018 [23]  Muhumuza et al., 2015b [12] | Sanya et al., 2017 [18] | Adeneye et al., 2007 [22] Fleming et al., 2009 [25] |
| **Beliefs and attitudes regarding the nature of SCH** | SCH not a major health concern | Lothe et al., 2018 [23] | Fleming et al., 2009 [25]  Pearson, 2016 [17]  Sanya et al., 2017 [18] | Lothe et al., 2018 [23] | Pearson, 2016 [17]  Sanya et al., 2017 [18] | Fleming et al., 2009 [25] |
|  | Traditional beliefs | Adeneye et al., 2007 [22]  Lothe et al., 2018 [23] | Fleming et al., 2009 [25] Parker et al., 2008 [28]  Pearson, 2016 [17] | Lothe et al., 2018 [23] | Parker et al., 2008 [28] Pearson, 2016 [17] | Adeneye et al., 2007 [22]  Fleming et al., 2009 [25] |
| **Knowledge of MDAs** | Access to operational information | Bukindu et al., 2016 [8]  Mafe et al., 2005 [1]  Ndyomugyenyi and Kabatereine, 2003 [2] | Bogus et al, 2016 [29] Chami et al., 2017 [6]  Odhiambo et al., 2016 [13]  Omedo et al., 2012 [14]  Omedo et al., 2014 [27]^○^  Tuhebwe et al., 2015 [19] | Bukindu et al., 2016 [8] ^○^ | Bogus et al, 2016 [29]  Chami et al., 2017 [6]  Omedo et al., 2012 [14]  Omedo et al., 2014 [27]^○^  Tuhebwe et al., 2015 [19] | Mafe et al., 2005 [1] Ndyomugyenyi and Kabatereine, 2003 [2]  Odhiambo et al., 2016 [13] |
|  | Unclear rationale of treatment | Hastings, 2016 [9]  Massa et al., 2009a [20]  Muhumuza et al., 2015b [12] | Chami et al., 2017 [6]  Odhiambo et al., 2016 [13]  Omedo et al., 2012 [14]  Parker et al., 2008 [28]  Parker and Allen, 2011 [15]  Pearson, 2016 [17]  Rilkoff et al., 2013 [7]^🞏^ | Hastings, 2016 [9]  Muhumuza et al., 2015b [12] | Chami et al., 2017 [6]  Omedo et al., 2012 [14]  Parker et al., 2008 [28]  Parker and Allen, 2011 [15]  Pearson, 2016 [17]  Rilkoff et al., 2013 [7]^🞏^ | Massa et al., 2009a [20]  Odhiambo et al., 2016 [13] |
|  | Unclear drug-administration procedure | Hastings, 2016 [9] | Fleming et al., 2009 [25]  Parker et al., 2008 [28]  Parker and Allen, 2011 [15] | Hastings, 2016 [9] | Parker et al., 2008 [28]  Parker and Allen, 2011 [15] | Fleming et al., 2009 [25] |
| **Beliefs and attitudes regarding MDAs’ effectiveness** | Perceived health benefits | Adeneye et al., 2007 [22] | Bogus et al., 2016 [29]  Fleming et al., 2009 [25]  Omedo et al., 2012 [14]  Parker et al., 2008 [28]  Parker and Allen, 2011 [15]  Pearson, 2016 [17] Sanya et al., 2017 [18]  Tuhebwe et al., 2015 [19] |  | Bogus et al., 2016 [29]  Omedo et al., 2012 [14]  Parker et al., 2008 [28]  Parker and Allen, 2011 [15]  Pearson, 2016 [17]  Sanya et al., 2017 [18] Tuhebwe et al., 2015 [19] | Adeneye et al., 2007 [22]  Fleming et al., 2009 [25] |
|  | Perceived competence of distributors | Hastings, 2016 [9]  Massa et al., 2009a [20]^○^ | Chami et al., 2017 [6]  Odhiambo et al., 2016 [13]  Omedo et al., 2012 [14]  Rilkoff et al., 2013 [7] | Hastings, 2016 [9] | Chami et al., 2017 [6]  Omedo et al., 2012 [14]  Rilkoff et al., 2013 [7] | Massa et al., 2009a [20]^○^  Odhiambo et al., 2016 [13] |
|  | Religious beliefs |  | Odhiambo et al., 2016 [13]  Omedo et al., 2012 [14] |  | Omedo et al., 2012 [14] | Odhiambo et al.. 2016 [13] |

| **Themes** | **Categories** | Target Populations | | Type of MDA | | |
| --- | --- | --- | --- | --- | --- | --- |
|  |  | SAC  (n=13) | SAC & Adults  (n=17) | SBT  (n=8) | CWT  (n=13) | MIXED (n=9) |
| INTERPERSONAL LEVEL | | | | | | |
| **Negative rumours of MDAs** | **Rumours of deaths or severe health consequences** | Hastings, 2016 [9] | Fleming et al., 2009 [25]  Odhiambo et al., 2016 [13]  Omedo et al., 2012 [14] Parker et al., 2008 [28]  Parker and Allen, 2011 [15]^▽^  Rilkoff et al., 2013 [7]^🞏^ | Hastings, 2016 [9] | Omedo et al., 2012 [14]  Parker et al., 2008 [28]  Parker and Allen, 2011 [15]^▽^  Rilkoff et al., 2013 [7]^🞏^ | Fleming et al., 2009 [25]  Odhiambo et al., 2016 [13] |
|  | **Rumours of a conspiracy by governments and foreign agencies** | Hastings, 2016 [9] | Odhiambo et al., 2016 [13]  Omedo et al., 2012 [14]  Parker et al., 2008 [28]  Parker and Allen, 2011 [15] | Hastings, 2016 [9] | Omedo et al., 2012 [14]  Parker et al., 2008 [28]  Parker and Allen, 2011 [15] | Odhiambo et al., 2016 [13] |
| **Social influence** | **Adults’ influence on SAC** | Muhumuza et al., 2013 [24]  Lothe et al., 2018 [23] | Fleming et al., 2009 [25]  Rilkoff et al., 2013 [7]^🞏^ | Muhumuza et al., 2013 [24]  Lothe et al., 2018 [23] | Rilkoff et al., 2013 [7]^🞏^ | Fleming et al., 2009 [25] |
|  | **Peer-pressure** | Lothe et al., 2018 [23] | Rilkoff et al., 2013 [7] | Lothe et al., 2018 [23] | Rilkoff et al., 2013 [7] |  |
|  | **Local social networks** |  | Adriko et al., 2018 [4]  Chami et al., 2017 [6] |  | Chami et al., 2017 [6] | Adriko et al., 2018 [4] |
| ORGANISATIONAL LEVEL | | | | | | |
| **Health systems’ support** | |  | Dabo et al., 2013 [5]  Fleming et al., 2009 [25]  Odhiambo et al., 2016 [13]  Omedo et al., 2012 [14] |  | Dabo et al, 2013 [5]  Omedo et al., 2012 [14] | Fleming et al., 2009 [25]  Odhiambo et al., 2016 [13] |
| COMMUNITY LEVEL | | | | | | |
| **Community engagement** | **Community support during MDAs** | Adeneye et al., 2007 [22]  Massa et al., 2009a [20]  Ndyomugyenyi and Kabatereine, 2003 (2) | Dabo et al. 2013 [5]  Omedo et al., 2014 [27] Parker and Allen, 2011 [15] |  | Dabo et al, 2013 [5]  Omedo et al., 2014 [27] Parker and Allen, 2011 [15] | Adeneye et al., 2007 [22]  Massa et al., 2009a [20] Ndyomugyenyi and Kabatereine, 2003 [2] |
|  | **Leaders’ perceptions of health benefits** | Adeneye et al., 2007 [22]  Massa et al., 2009a [20] |  |  |  | Adeneye et al., 2007 [22]  Massa et al., 2009a [20] |
| **Socio-cultural trajectories** | **Social cohesion** |  | Chami et al., 2016 [30]  Chami et al., 2017 [6]  Dabo et al. 2013 [5]  Parker et al., 2008 [28] |  | Chami et al., 2016 [30]  Chami et al., 2017 [6]  Dabo et al., 2013 [5]  Parker et al., 2008 [28] |  |
|  | **Gender values** |  | Dabo et al., 2013 [5]  Odhiambo et al., 2016 [13]  Omedo et al., 2012 [14]  Parker and Allen, 2011 [15] |  | Dabo et al., 2013 [5]  Omedo et al., 2012 [14]  Parker and Allen, 2011 [15] | Odhiambo et al., 2016 [13] |
|  | **Past public health campaigns** |  | Parker and Allen, 2011 [15] Pearson, 2016 [17] |  | Parker and Allen, 2011 [15] Pearson, 2016 [17] |  |
| **Geographical features** | **Setting’s size** | Mafe et al., 2005 [1]  Massa et al., 2009a [20] Randjelovic et al., 2015 [3] ^◊^ | Adriko et al., 2018 [4]  Chami et al., 2017 [6]  Coulibaly et al 2018 [16]  Dabo et al., 2013 [5] | Randjelovic et al., 2015 [3] ^◊^ | Chami et al., 2017 [6]  Coulibaly et al 2018 [16]  Dabo et al., 2013 [5] | Adriko et al.2018 [4] Mafe et al., 2005 [1]  Massa et al., 2009a [20] |
|  | **Migration patterns** |  | Parker and Allen, 2011 [15]  Pearson, 2016 [17] |  | Parker and Allen, 2011 [15]  Pearson, 2016 [17] |  |

| **Themes** | **Categories** | Target Populations | | Type of MDA | | |
| --- | --- | --- | --- | --- | --- | --- |
|  |  | SAC  (n=13) | SAC & Adults  (n=17) | SBT  (n=8) | CWT  (n=13) | MIXED (n=9) |
| Policy Level | | | | | | |
| **Sensitisation** | **Content** | Adeneye et al., 2007 [22]  Hastings, 2016 [9]  Massa et al., 2009a [20]  Muhumuza et al., 2014 [10]  Muhumuza et al., 2015b [12] | Odhiambo et al., 2016 [13]  Omedo et al., 2012 [14]  Omedo et al., 2014 [27]^○^  Parker et al., 2008 [28]  Parker and Allen, 2011 [15] | Hastings, 2016 [9]  Muhumuza et al., 2014 [10]  Muhumuza et al., 2015b [12] | Omedo et al., 2012 [14]  Omedo et al., 2014 [27]^○^  Parker et al., 2008 [28]  Parker and Allen, 2011 [15] | Adeneye et al., 2007 [22] Massa et al., 2009a [20]  Odhiambo et al., 2016 [13] |
|  | **Training for distributors** | Muhumuza et al., 2015b [12] | Parker et al., 2008 [28]  Parker and Allen, 2011 [15] Rilkoff et al., 2013 [7]  Tuhebwe et al., 2015 [19] | Muhumuza et al., 2015b [12] | Parker et al., 2008 [28]  Parker and Allen, 2011 [15] Rilkoff et al., 2013 [7] Tuhebwe et al., 2015 [19] |  |
|  | **Means of sensitisation** | Hastings, 2016 [9]  Massa et al., 2009a [20] | Fleming et al., 2009 [25]  Odhiambo et al., 2016 [13]  Omedo et al., 2014 [27]^○^  Tuhebwe et al., 2015 [19] | Hastings, 2016 [9] | Omedo et al., 2014 [27]^○^  Tuhebwe et al., 2015 [19] | Fleming et al., 2009 [25]  Massa et al., 2009a [20]  Odhiambo et al., 2016 [13] |
|  | **Length of time** | Hastings, 2016 [9] | Fleming et al. 2009 [25] | Hastings, 2016 [9] |  | Fleming et al., 2009 [25] |
| **Incentives for distributors** | **Material incentives / compensation for distributors** | Adeneye et al., 2007 [22]  Massa et al., 2009a [20]  Muhumuza et al., 2015b [12] | Dabo et al., 2013 [5] Fleming et al. 2009 [25]  Odhiambo et al., 2016 [13]  Omedo et al., 2012 [14]  Parker et al., 2008 [28] Parker and Allen, 2011 [15]  Rilkoff et al., 2013 [7]  Tuhebwe et al., 2015 [19] | Muhumuza et al., 2015b [12] | Dabo et al., 2013 [5]  Omedo et al., 2012 [14]  Parker et al., 2008 [28] Parker and Allen, 2011 [15]  Rilkoff et al., 2013 [7]  Tuhebwe et al., 2015 [19] | Adeneye et al., 2007 [22] Fleming et al., 2009 [25]  Massa et al., 2009a [20]  Odhiambo et al., 2016 [13] |
|  | **Immaterial rewards for distributors** | Adeneye et al., 2007 [22]  Massa et al., 2009a [20]  Ndyomugyenyi and Kabatereine, 2003 [2] | Chami et al., 2017 [6]  Omedo et al., 2012 [14]  Parker et al., 2008 [28] Parker and Allen, 2011 [15] |  | Chami et al., 2017 [6]  Omedo et al., 2012 [14]  Parker et al., 2008 [28] Parker and Allen, 2011 [15] | Adeneye et al., 2007 [22]  Massa et al., 2009a [20] Ndyomugyenyi and Kabatereine, 2003 [2] |
| **Design of MDA operations** | **Distribution strategies** | Adeneye et al., 2007 [22]  Mafe et al., 2005 [1]  Massa et al., 2009a [20]  Massa et al., 2009b [21]  Ndyomugyenyi and Kabatereine, 2003 [2] | Coulibaly et al 2018 [16]  Dabo et al., 2013 [5] |  | Coulibaly et al 2018 [16]  Dabo et al., 2013 [5] | Adeneye et al., 2007 [22]  Mafe et al., 2005 [1]  Massa et al., 2009a [20]  Massa et al., 2009b [21]  Ndyomugyenyi and Kabatereine, 2003 [2] |
|  | **Organisational structure** |  | Fleming et al., 2009 [25]  Omedo et al., 2012 [14] |  | Omedo et al., 2012 [14] | Fleming et al., 2009 [25] |
| ⭘ Study discusses strategies designed to address this specific barrier.  🞏 Variable mediates the effect of gender.  △ Variable mediates the effect of ethnicity. ▽ Paper reports decreasing effects of barrier over time.  ◊ Item constitutes an organisational (school-based) issue. Reported as a community factor for thematic consistency with other publications. | | | | | | |

**References**

1. Mafe MA, Appelt B, Adewale B, Idowu ET, Akinwale OP, Adeneye AK, et al. Effectiveness of different approaches to mass delivery of praziquantel among school-aged children in rural communities in Nigeria. Acta Trop. 2005 Feb;93(2):181–90.

2. Ndyomugyenyi R, Kabatereine N. Integrated community-directed treatment for the control of onchocerciasis, schistosomiasis and intestinal helminths infections in Uganda: advantages and disadvantages. Trop Med Int Health. 2003;8(11):997–1004.

3. Randjelovic A, Frønæs SG, Munsami M, Kvalsvig JD, Zulu SG, Gagai S, et al. A study of hurdles in mass treatment of schistosomiasis in KwaZulu-Natal, South Africa. South Afr Fam Pract. 2015 Mar 4;57(2):57–61.

4. Adriko M, Faust CL, Carruthers LV, Moses A, Tukahebwa EM, Lamberton PHL. Low Praziquantel Treatment Coverage for Schistosoma mansoni in Mayuge District, Uganda, Due to the Absence of Treatment Opportunities, Rather Than Systematic Non-Compliance. Trop Med Infect Dis. 2018 Oct 8;3(4).

5. Dabo A, Bary B, Kouriba B, Sankare O, Doumbo O. Factors associated with coverage of praziquantel for schistosomiasis control in the community-direct intervention (CDI) approach in Mali (West Africa). Infect Dis Poverty. 2013;2:11.

6. Chami GF, Kontoleon AA, Bulte E, Fenwick A, Kabatereine NB, Tukahebwa EM, et al. Community-directed mass drug administration is undermined by status seeking in friendship networks and inadequate trust in health advice networks. Soc Sci Med. 2017 Jun 1;183:37–47.

7. Rilkoff H, Tukahebwa EM, Fleming FM, Leslie J, Cole DC. Exploring Gender Dimensions of Treatment Programmes for Neglected Tropical Diseases in Uganda. PLoS Negl Trop Dis. 2013 Jul;7(7):e2312.

8. Bukindu F, Morona D, Mazigo HD. Prevalence of Schistosoma mansoni and soil transmitted helminths and factors associated with uptake of preventive chemotherapy among school children in Sengerema District in north-western Tanzania. Tanzan J Health Res [Internet]. 2016 Jan 1 [cited 2018 Feb 14];18(1). Available from: https://www.ajol.info/index.php/thrb/article/view/122544

9. Hastings J. Rumours, Riots and the Rejection of Mass Drug Administration for the Treatment of Schisotosomiasis in Morogoro, Tanzania. J Biosoc Sci. 2016 Sep;48(S1):S16–39.

10. Muhumuza S, Olsen A, Katahoire A, Kiragga AN, Nuwaha F. Effectiveness of a Pre-treatment Snack on the Uptake of Mass Treatment for Schistosomiasis in Uganda: A Cluster Randomized Trial. PLOS Med. 2014 May 13;11(5):e1001640.

11. Muhumuza S, Olsen A, Katahoire A, Nuwaha F. Reduced uptake of mass treatment for schistosomiasis control in absence of food: beyond a randomized trial. BMC Infect Dis. 2015;15(1):423.

12. Muhumuza S, Olsen A, Nuwaha F, Katahoire A. Understanding Low Uptake of Mass Treatment for Intestinal Schistosmiasis Among School Children: A Qualitative Study in Jinja District, Uganda. J Biosoc Sci. 2015 Jul;47(4):505–20.

13. Odhiambo GO, Musuva RM, Odiere MR, Mwinzi PN. Experiences and perspectives of community health workers from implementing treatment for schistosomiasis using the community directed intervention strategy in an informal settlement in Kisumu City, western Kenya. BMC Public Health [Internet]. 2016 Sep 15 [cited 2018 Feb 18];16. Available from: https://www.ncbi.nlm.nih.gov/pmc/articles/PMC5025566/

14. Omedo MO, Matey EJ, Awiti A, Ogutu M, Alaii J, Karanja DMS, et al. Community Health Workers’ Experiences and Perspectives on Mass Drug Administration for Schistosomiasis Control in Western Kenya: The SCORE Project. Am J Trop Med Hyg. 2012 Dec 5;87(6):1065–72.

15. Parker M, Allen T. Does mass drug administration for the integrated treatment of neglected tropical diseases really work? Assessing evidence for the control of schistosomiasis and soil-transmitted helminths in Uganda. Health Res Policy Syst. 2011 Jan 6;9:3.

16. Coulibaly JT, Ouattara M, Barda B, Utzinger J, N’Goran EK, Keiser J. A Rapid Appraisal of Factors Influencing Praziquantel Treatment Compliance in Two Communities Endemic for Schistosomiasis in Côte d’Ivoire. Trop Med Infect Dis. 2018 Jun 19;3(2).

17. Pearson G. Low Prevalence of Intestinal Schistosomiasis Among Fisherfolk Living Along the River Nile in North-Western Uganda: A Biosocial Investigation. J Biosoc Sci. 2016 Sep;48(S1):S74–91.

18. Sanya RE, Tumwesige E, Elliott AM, Seeley J. Perceptions about interventions to control schistosomiasis among the Lake Victoria island communities of Koome, Uganda. PLoS Negl Trop Dis. 2017 Oct 2;11(10):e0005982.

19. Tuhebwe D, Bagonza J, Kiracho EE, Yeka A, Elliott AM, Nuwaha F. Uptake of Mass Drug Administration Programme for Schistosomiasis Control in Koome Islands, Central Uganda. PLOS ONE. 2015 Apr 1;10(4):e0123673.

20. Massa K, Magnussen P, Sheshe A, Ntakamulenga R, Ndawi B, Olsen A. Community Perceptions on the Community-Directed Treatment and School-Based Approaches for the Control of Schistomiasis and Soil-Transmitted Helminthiasis Amoong School-Age Children in Lushoto District, Tanzania. J Biosoc Sci. 2009 Jan;41(01):89.

21. Massa K, Olsen A, Sheshe A, Ntakamulenga R, Ndawi B, Magnussen P. Can coverage of schistosomiasis and soil transmitted helminthiasis control programmes targeting school-aged children be improved? New approaches. Parasitology. 2009 Nov;136(13):1781–8.

22. Adeneye AK, Akinwale OP, Idowu ET, Adewale B, Manafa OU, Sulyman MA, et al. Sociocultural aspects of mass delivery of praziquantel in schistosomiasis control: The Abeokuta experience. Res Soc Adm Pharm. 2007 Jun 1;3(2):183–98.

23. Lothe A, Zulu N, Øyhus AO, Kjetland EF, Taylor M. Treating schistosomiasis among South African high school pupils in an endemic area, a qualitative study. BMC Infect Dis [Internet]. 2018 May 25 [cited 2019 Feb 13];18. Available from: https://www.ncbi.nlm.nih.gov/pmc/articles/PMC5970489/

24. Muhumuza S, Olsen A, Katahoire A, Nuwaha F. Uptake of Preventive Treatment for Intestinal Schistosomiasis among School Children in Jinja District, Uganda: A Cross Sectional Study. PLOS ONE. 2013 May 7;8(5):e63438.

25. Fleming FM, Fenwick A, Tukahebwa EM, Lubanga RGN, Namwangye H, Zaramba S, et al. Process evaluation of schistosomiasis control in Uganda, 2003 to 2006: perceptions, attitudes and constraints of a national programme. Parasitology. 2009 Nov;136(13):1759–69.

26. Knopp S, Person B, Ame SM, Ali SM, Muhsin J, Juma S, et al. Praziquantel coverage in schools and communities targeted for the elimination of urogenital schistosomiasis in Zanzibar: a cross-sectional survey. Parasit Vectors. 2016 Jan 4;9(1):5.

27. Omedo M, Ogutu M, Awiti A, Musuva R, Muchiri G, Montgomery SP, et al. The Effect of a Health Communication Campaign on Compliance with Mass Drug Administration for Schistosomiasis Control in Western Kenya?The SCORE Project. Am J Trop Med Hyg. 2014 Nov 5;91(5):982–8.

28. Parker M, Allen T, Hastings J. Resisting Control of Neglected Tropical Diseases: Dilemmas in the Mass Treatment of Schistosomiasis and Soil-Transmitted Helminghts in North-West Uganda. J Biosoc Sci. 2008 Mar;40(2):161–81.

29. Bogus J, Gankpala L, Fischer K, Krentel A, Weil GJ, Fischer PU, et al. Community Attitudes toward Mass Drug Administration for Control and Elimination of Neglected Tropical Diseases after the 2014 Outbreak of Ebola Virus Disease in Lofa County, Liberia. Am J Trop Med Hyg. 2016 Mar 2;94(3):497–503.

30. Chami GF, Kontoleon AA, Bulte E, Fenwick A, Kabatereine NB, Tukahebwa EM, et al. Profiling Nonrecipients of Mass Drug Administration for Schistosomiasis and Hookworm Infections: A Comprehensive Analysis of Praziquantel and Albendazole Coverage in Community-Directed Treatment in Uganda. Clin Infect Dis. 2016 Jan 15;62(2):200–7.
